# High expression of six-transmembrane epithelial antigen of prostate 3 promotes the migration and invasion and predicts unfavorable prognosis in glioma

**DOI:** 10.7717/peerj.15136

**Published:** 2023-03-28

**Authors:** Langmei Deng, Shuangshuang Zeng, Qiaoli Yi, Liying Song

**Affiliations:** 1 Department of Emergency, The Third Xiangya Hospital, Central South University, Changsha, Hunan, China; 2 Department of Pharmacy, Xiangya Hospital, Central South University, Changsha, Hunan, China; 3 Department of Pharmacy, The Third Xiangya Hospital, Central South University, Changsha, Hunan, China

**Keywords:** Glioma, STEAP3, Metastasis, Immune infiltration, Prognosis

## Abstract

Recent studies have suggested that ferroptosis, a form of iron-dependent regulated cell death, might play essential roles in tumor initiation and progression. Six-transmembrane epithelial antigen of prostate 3 (STEAP3) is a ferrireductase involved in the regulation of intracellular iron homeostasis. However, the clinical significance and biological function of STEAP3 in human cancers remain poorly understood. Through a comprehensive bioinformatics analysis, we found that STEAP3 mRNA and protein expression were up-regulated in GBM, LUAD, and UCEC, and down-regulated in LIHC. Survival analysis indicated that STEAP3 had prognostic significance only in glioma. Multivariate Cox regression analysis revealed that high STEPA3 expression was correlated with poor prognosis. STEAP3 expression was significantly negatively correlated with promoter methylation level, and patients with lower STEAP3 methylation level had worse prognosis than those with higher STEAP3 methylation level. Single-cell functional state atlas showed that STEAP3 regulated epithelial-to-mesenchymal transition (EMT) in GBM. Furthermore, the results of wound healing and transwell invasion assays demonstrated that knocking down STEAP3 inhibited the migration and invasion of T98G and U251 cells. Functional enrichment analysis suggested that genes co-expressed with STEAP3 mainly participated in inflammation and immune-related pathways. Immunological analysis revealed that STEAP3 expression was significantly correlated with immune infiltration cells, including macrophages and neutrophils, especially the M2 macrophages. Individuals with low STEAP3 expression were more likely to respond to immunotherapy than those with high STEAP3 expression. These results suggest that STEAP3 promotes glioma progression and highlight its pivotal role in regulating immune microenvironment.

## Introduction

Glioma is the most common and fatal primary central nervous system tumor, characterized by a poor prognosis, with a 5-year overall survival rate of only 6.8% for high-grade glioma due to limited effectiveness of surgical resection and chemoradiotherapy (Magalhaes et al., 2021; Thorbinson & Kilday, 2021; Zhang et al., 2021b). Isocitrate dehydrogenase (IDH) mutations and chromosome arms 1p and 19q co-deletion have been identified as molecular pathological markers in glioma, indicating a significant survival benefit (Ceccarelli et al., 2016; Louis et al., 2021). However, due to the highly invasive and infiltrative nature of glioma cells, current therapeutic regimes and disease monitoring means have achieved limited clinical success (Magalhaes et al., 2021; Xu et al., 2021). There is an urgent need to identify novel biomarkers for early diagnosis and prognosis prediction in glioma patients.

Six-transmembrane epithelial antigen of prostate 3 (STEAP3) is located on chromosome 2q14.2 and encodes a multi-pass transmembrane protein that acts as an iron transporter. STEAP3 can reduce iron from Fe^3+^ to Fe^2+^ state, and plays an essential role in mediating intracellular iron homeostasis (Ohgami et al., 2005; Ohgami et al., 2006). The dysregulation of iron metabolism is tightly linked with ferroptosis, a form of regulated cell death modality induced by iron-dependent phospholipids peroxidation on cellular membranes (Lei, Zhuang & Gan, 2022). Liu et al. (2021) identified that STEAP3 knockdown blocked erastin or RSL3-induced ferroptosis. Accumulating evidence has implicated that ferroptosis participates in the development of diverse cancer types and affects the response to therapies (Chen et al., 2021b; Qu, Peng & Liu, 2022). Mesenchymal and dedifferentiated tumor cells, associated with resistance to common therapeutics, are susceptible to ferroptosis inducers (Tsoi et al., 2018; Viswanathan et al., 2017). Ferroptosis induction might be a promising strategy for cancer treatment.

In order to explore the role of STEAP3 in glioma, we first comprehensively analyzed STEAP3 expression profiles, methylation, and its clinical implications with datasets acquired from The Cancer Genome Atlas (TCGA) and the Chinese Glioma Genome Atlas (CGGA). Gene Ontology (GO) and Kyoto Encyclopedia of Genes and Genomes (KEGG) pathway analyses were used to explore the potential molecular mechanisms of STEAP3 and its co-expressed genes. In addition, we analyzed the correlation between STEAP3 expression and immune infiltration. In general, our study indicated that STEAP3 might function as a potential prognostic biomarker in gliomas through immune regulation.

## Materials and Methods

### Gene expression and survival analysis

The integrative bioinformatics analysis of STEAP3 in multiple cancer types was achieved with several bioinformatics databases (Table 1). Tumor Immune Estimation Resource (TIMER2.0) is a web portal for systematical analysis of immune infiltration across various cancer types (Li et al., 2020). We used the Gene_DE module of TIMER2.0 to explore the differential expression of STEAP3 gene between tumor samples and normal tissues. Gene expression levels were normalized by log2 (transcripts per million, TPM) prior to analysis. For certain tumor types without adjacent normal tissues, the Xiantao tool (https://www.xiantao.love/products) was further applied to explore the differences in STEAP3 expression between TCGA cancer samples and matched TCGA normal tissues and data from the Genotype-Tissue Expression (GTEx) database. Gene expression levels were normalized by log2 (TPM + 1). Through the Xiantao tool, comprehensive bioinformatics analysis can be performed across diverse cancer types, including differential expression analysis, interaction network, functional enrichment analysis, *etc*. Univariate and multivariate Cox regression analysis were carried out to assess the effects of the independent variables on survival using the Xiantao tool. In addition, we also employed the Xiantao tool to assess the prognostic value of STEAP3 in different cancer types. The main outcomes included overall survival (OS), disease-specific survival (DSS), and progression-free survival (PFS). Median STEAP3 expression served as a cutoff to discriminate high- and low-expression groups.

**Table 1:** Integrative bioinformatics analyzed in the study.

Database	URL	References
TIMER2.0	http://timer.cistrome.org/	Li et al. (2020)
UALCAN	http://ualcan.path.uab.edu/	Chandrashekar et al. (2017)
HPA	https://www.proteinatlas.org/	Uhlen et al. (2017)
CGGA	http://www.cgga.org.cn/	Zhao et al. (2021b)
CancerSEA	http://biocc.hrbmu.edu.cn/CancerSEA/home.jsp	Yuan et al. (2019)
LinkedOmics	http://linkedomics.org/login.php	Vasaikar et al. (2018)
TISIDB	http://cis.hku.hk/TISIDB	Ru et al. (2019)
TIDE	http://tide.dfci.harvard.edu/	Fu et al. (2020)

The University of Alabama at Birmingham cancer data analysis portal (UALCAN) is an open-access online database for analyzing cancer omics data (Chandrashekar et al., 2017). The protein expression of STEAP3 was explored using data from the Clinical Proteomic Tumor Analysis Consortium (CPTAC) dataset (Chen et al., 2019). Immunohistochemistry (IHC) staining was performed to assess the differential expression of STEAP3 at the protein level. The Human Protein Atlas (HPA) (Uhlen et al., 2017) was applied to evaluate STEAP3 protein expression in tumor samples and the corresponding normal tissues.

### DNA methylation analysis

Aberrant DNA methylation is associated with gene expression, and impacts outcomes for patients with cancer (Pogribna & Hammons, 2021). CGGA is a web application that provides correlation and survival analysis in Chinese glioma cohorts based on mRNA expression and DNA methylation data (Zhao et al., 2021b). The demographic distribution of STEAP3 methylation and its prognostic value in glioma were analyzed by CGGA. The Xiantao tool was applied to explore the correlation between the expression level of STEAP3 and its promoter DNA methylation degree. Promoter was defined as the 2.1 kb surrounding the transcription start site (TSS) (−2,000 bp/+100 bp) of RefSeq genes.

### Single cell sequencing data analysis and gene set enrichment analysis (GSEA)

The Cancer Single-cell State Atlas database (CancerSEA) (Yuan et al., 2019) was applied to explore the relevance of STEAP3 across 14 functional states in various cancer types. We downloaded the correlation data and then generated the heatmap. The LinkedOmics database is an open-access portal containing multi-omics data across different cancer types and features three analysis modules: LinkFinder, LinkInterpreter, and LinkCompare (Vasaikar et al., 2018). Heatmaps of the top 50 genes positively or negatively correlated with STEAP3 were generated with the LinkFinder module. Furthermore, the LinkInterpreter module was used to perform the GO-biological process (GO-BP) and KEGG pathway analysis.

### Tumor immune infiltrate analysis and prediction of immunotherapy responses

The interaction between glioma and immune system were performed using the Xiantao tool and the TISIDB database (Ru et al., 2019). First, we used the Xiantao tool to generate the Lollipop diagrams of 24 immune cell types in the TCGA-GBMLGG dataset. Then, the relations between STEAP3 expression and abundance of tumor-infiltrating immune cells were cross-validated using the TISIDB database. In addition, the immune score of each glioma sample was estimated using the “ESTIMATE” R package based on expression data (Yoshihara et al., 2013). The relationship between STEAP3 expression and immune score was visualized with scatterplot. We divided glioma patients into high and low immune score groups based on the median values of immune score, and then assessed the prognostic value of immune score in glioma.

Immune checkpoint proteins, such as programmed cell death protein 1 (PD-1), programmed cell death 1 ligand 1 (PD-L1) and cytotoxic T-lymphocyte associated protein 4 (CTLA4), play a critical role in tumor immune escape. Immune checkpoint inhibitors (ICI)-based immunotherapy could produce potent and durable antitumor response (Kraehenbuehl et al., 2022). The tumor immune dysfunction and exclusion (TIDE) algorithm was applied to predict the immunotherapy response of glioma patients based on pre-treatment expression profiles (Fu et al., 2020).

### Cell cultures, reagents, and small interfering RNAs (siRNAs) transfections

Human GBM cell lines T98G and U251 were gifted from the Cancer Research Institute of the Central South University (Changsha, China), as described in our previous study (Yi et al., 2022). Two GBM cell lines were incubated in DMEM (C11995500; HyClone, Logan, UT, USA) supplemented with 10% fetal bovine serum (11570506; Gibco, Billings, MT, USA) and 1% penicillin and streptomycin (10378016; Gibco, Billings, MT, USA) at 37 °C with 5% CO_2_. For transient transfection, cells were plated in complete serum-containing medium the day before transfection, then transfected with siRNAs of STEAP3 using Lipofectamine™ 3000 Reagent (L3000075, Invitrogen, Waltham, MA, USA) in DMEM. Two STEAP3 siRNAs were purchased form Genechem company (Si1, GTCTGCTTCTATGCCTACA; Si2, CCCTCTACAGCTTCTGCTT). A total of 8 h after transfection, the serum-free medium was replaced with complete serum-containing medium, and cells were collected 24 h after transfection for subsequent experimental studies.

### Western blot

The glioma cells transfected with siNC or STEAP3 siRNAs were lysed with RIPA buffer supplemented with protease inhibitor cocktails (B14012; Bimake, Houston, TX, USA). The BCA protein assay kit (23229; Thermo Fisher Scientific, Waltham, MA, USA) was applied to determine protein concentrations. Protein was transferred into 0.45 µm PVDF membranes (IPVH00010; Millipore, Burlington, MA, USA) after SDS-polyacrylamide gel electrophoresis. Then, the membranes were blocked with 5% nonfat dry milk for 1 h at room temperature. Primary antibodies against STEAP3 (PA5-20406; Thermo Fisher Scientific, Waltham, MA, USA) and β-actin (sc-58673; Santa, Dallas, TX, USA) was incubated overnight at 4 °C. The protein bands were visualized with Immobilon Western chemiluminescent HRP reagents (WBKLS0500; Millipore, Burlington, MA, USA).

### Wound healing and transwell invasion assay

Changes in migration and invasion abilities were examined by wound healing and invasion assays, respectively. T98G and U251 cells were cultured to complete confluence in medium containing 10% FBS. The linear wound was created using a plastic scraper. After washed twice with PBS, the medium was replaced with serum-free medium and cultured at 37 °C for 24 h. Then, the wound was observed under a microscope at 0, 12 and 24 h (Olympus, Tokyo, Japan). In invasion assay, Matrigel was purchased from BD Biosciences and thawed overnight at 4 °C. Take 30 µl of Matrigel diluted in serum-free DMEM and inoculate evenly into the upper chamber at 37 °C. Assays were performed using Transwell chambers (8 µm pore-size; Corning, Corning, NY, USA). The lower chambers were loaded with 600 µl of DMEM with 20% FBS. After 24 h of incubation, invaded cells were fixed with 4% paraformaldehyde and stained with 5% crystal violet. Cells on the lower surface of the membrane were counted under a microscope (Olympus, Tokyo, Japan).

### Statistical analysis

All experiments and assays were conducted and repeated at least three times, and results were presented as mean ± standard deviation (SD). Statistically significant differences were performed using the T-test or Wilcoxon test for pairwise comparisons or ANOVA for multivariate analysis. Kaplan–Meier survival curves were performed by the log-rank test. Correlation was analyzed using Spearman test. Based on the expression data, the immune score for each glioma sample was estimated using the “ESTIMATE” R package. GraphPad Prism 8 software was used for statistical analysis and *P* < 0.05 was considered as significance.

## Results

### Expression level of STEAP3 in pan-cancer

**Figure 1:**
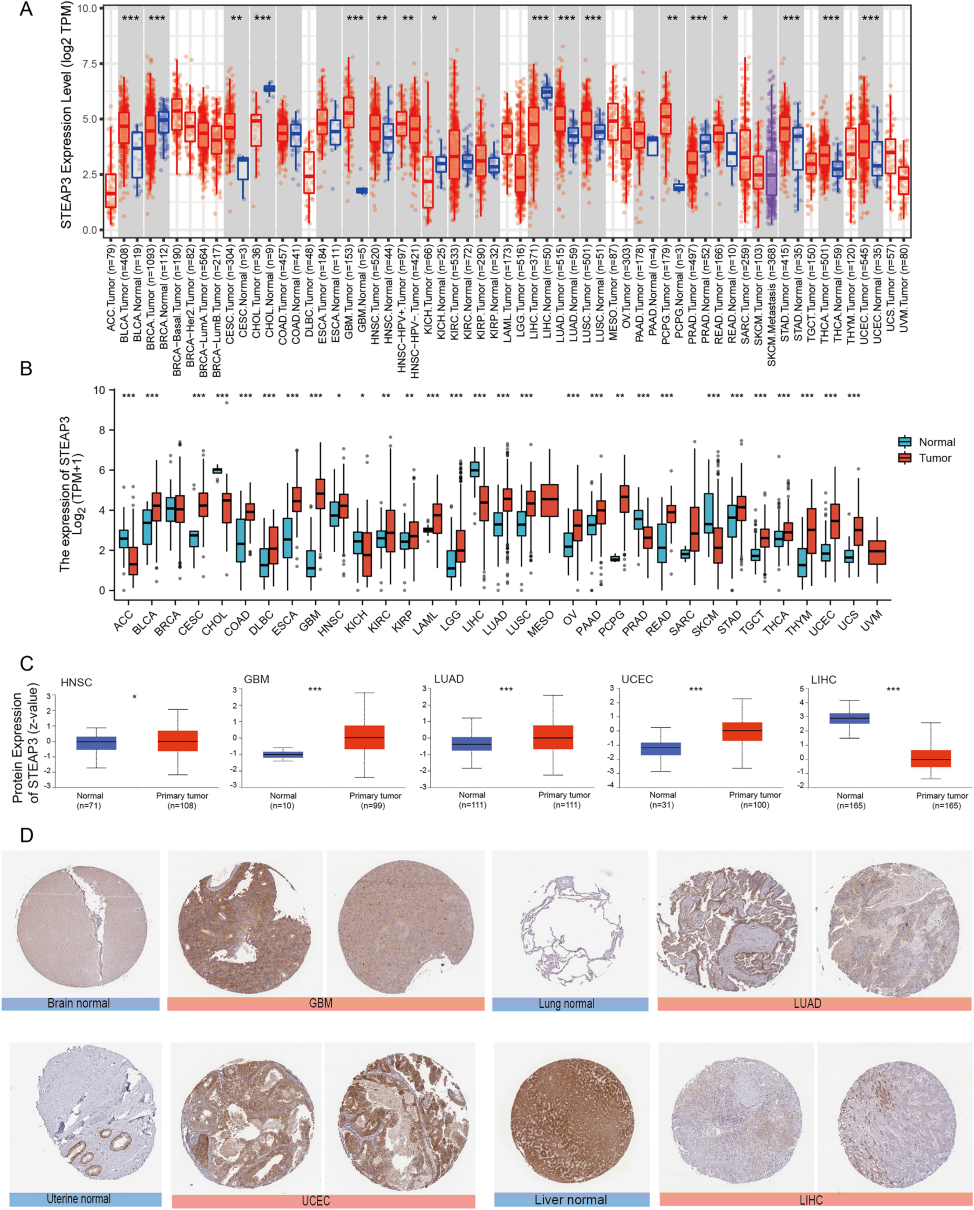
Expression level of STEAP3 in pan-cancer. (A) The mRNA expression levels of STEAP3 in TCGA cancer types from the TIMER2.0 database. Red and blue bar charts represent tumor samples and normal tissues, respectively. (B) Pan-cancer expression landscape of STEAP3 across TCGA and GTEx data from the Xiantao database. (C) The protein expression levels of STEAP3 in HNSC, GBM, LUAD, UCEC, and LIHC, analyzed by CPTAC. (D) The immunohistochemical analysis performed on GBM, LUAD, UCEC, and LIHC and corresponding normal tissues using the HPA database. *P* values: * represents *P* < 0.05, ** represents *P* < 0.01, and *** represents *P* < 0.001.

We used the TIMER2.0 database to explore the differential expression of STEAP3 between tumor samples and adjacent normal tissues. The flowchart was provided in Fig. S1. As shown in Fig. 1A, significantly differential expression of STEAP3 was found in 16 cancer types, with 11 tumor types up-regulated (BLCA, CESC, GBM, HNSC, LUAD, LUSC, PCPG, READ, STAD, THCA, and UCEC), and five tumor types down-regulated (BRCA, CHOL, KICH, LIHC, and PRAD). For certain cancer types without matched normal tissues in the TMIER2.0 database, we further explored the expression profile of STEAP3 using the Xiantao tool. Compared with the TCGA normal tissues and GTEx data, STEAP3 expression was down-regulated in ACC, CHOL, KICH, LIHC, PRAD, and SKCM, up-regulated in BLCA, CESC, COAD, DLBC, ESCA, GBM, HNSC, KIRC, KIRP, LAML, LGG, LUAD, LUSC, OV, PAAD, PCPG, READ, STAD, TGCT, THCA, THYM, UCEC, and UCS (Fig. 1B). In general, the expression of STEAP3 was elevated in the majority of 32 TCGA tumor types.

Besides transcript levels, using the CPTAC dataset, we further verified that the levels of STEAP3 protein were significantly higher in HNSC, GBM, LUAD, and UCEC samples than in normal tissues, while the levels in LIHC were lower than in normal tissues (Fig. 1C). Then we evaluated immunohistochemical (IHC) staining of STEAP3 proteins in tumor samples and normal tissues using the HPA database. Consistent with protein expression levels in the UALCAN data portal, IHC results revealed that liver tissues had strong STEAP3 IHC staining, while LIHC samples had weak staining. Normal brain, lung, and uterine tissues showed weak or moderate STEAP3 IHC staining, while GBM, LUAD, and UCEC samples exhibited moderate or strong staining (Fig. 1D). However, there was no clear difference in staining intensity between normal tonsil tissue and HNSC (Fig. S2).

### The prognostic value of STEAP3 and its correlation with clinicopathological characteristics in glioma

**Figure 2:**
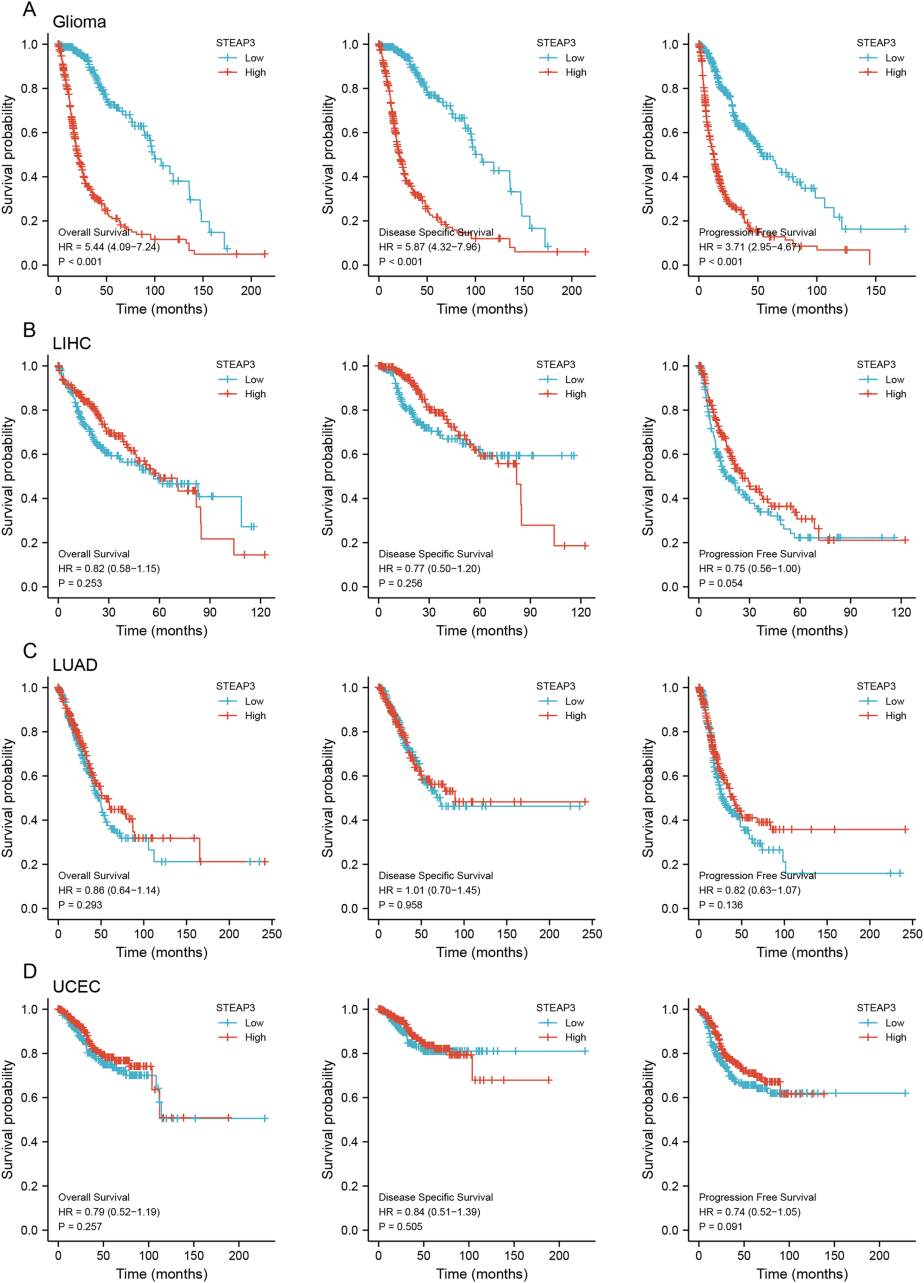
Prognostic value of STEAP3 in glioma, LIHC, LUAD, and UCEC. (A–D) Kaplan-Meier survival curves for overall survival, disease-specific survival, and progression free survival of STEAP3 in glioma (A), LIHC (B), LUAD (C), and UCEC (D).

To assess the clinical significance of STEAP3 expression in GBM, LUAD, UCEC, and LIHC, we performed Kaplan-Meier survival analysis for overall survival (OS), disease-specific survival (DSS), and progression-free survival (PFS) with the Xiantao tool. The results showed that high expression of STEAP3 was associated with poor OS (HR = 1.44; 95% CI [1.03–2.03]; *P* = 0.035) and poor PFS (HR = 1.66; 95% CI [1.18–2.33]; *P* = 0.004) in GBM (Fig. S3A). Given the lack of normal control tissues in the TCGA-LGG cohort, we added GTEx data to identify the high expression of STEAP3 in LGG. Then we explored the prognostic value of STEAP3 in LGG. As shown in Fig.S3B, patients with higher STEAP3 expression levels had significantly poorer OS (HR = 2.52; 95% CI [1.74–3.63]; *P* < 0.001), DSS (HR = 2.90; 95% CI [1.95–4.30]; *P* < 0.001), and PFS (HR = 2.30; 95% CI [1.73–3.06]; *P* < 0.001). Considering the consistent prognostic significance of STEAP3 in GBM and LGG, we further evaluated the prognostic value of STEAP3 in glioma. As presented in Fig. 2A, high expression levels of STEAP3 also correlated with poorer OS (HR = 5.44; 95% CI [4.09–7.24]; *P* < 0.001), DSS (HR = 5.87; 95% CI [4.32–7.96]; *P* < 0.001), and PFS (HR = 3.71; 95% CI [2.95–4.67]; *P* < 0.001). Kaplan-Meier survival analysis showed no statistical significance of STEAP3 in LUAD, UCEC, and LIHC (Figs. 2B–2D). Therefore, we mainly studied the effect of STEAP3 in glioma.

Univariate Cox regression analysis of seven independent variables in TCGA-GBMLGG cohort was carried out. Age, WHO grade, IDH status, 1p/19q co-deletion, and STEAP3 expression level, demonstrated a significant prognostic impact on OS. Multivariate Cox regression analysis exhibited that age (HR = 1.496; 95% CI [1.096–2.042]; *P* = 0.011), WHO grade (HR = 2.622; 95% CI [1.828–3.760]; *P* < 0.001), IDH status (HR = 0.305; 95% CI [0.198–0.470]; *P* < 0.001), and STEAP3 expression level (HR = 1.673; 95% CI [1.110–2.522]; *P* = 0.014) were independent prognostic factors for glioma patients (Table 2). Univariate Cox regression analysis also suggested an association between PFS and age, WHO grade, IDH status, 1p/19q co-deletion, and STEAP3 expression level. Multivariate Cox regression analysis indicated that WHO grade (HR = 1.974; 95% CI [1.435–2.716]; *P* < 0.001), IDH status (HR = 0.290; 95% CI [0.200–0.421]; *P* < 0.001), and STEAP3 expression level (HR = 1.450; 95% CI [1.045–2.013]; *P* = 0.026) were independent prognostic factors for glioma patients (Table 3). These results suggest that higher STEAP3 expression was associated with worse prognosis.

**Table 2:** Univariate and multivariable Cox regression of STEAP3 expression for overall survival in TCGA-GBMLGG cohorts.

Characteristics	Univariate Cox regression		Multivariate Cox regression	
	Hazard ratio (95% CI)	*P* value	Hazard ratio (95% CI)	*P* value
WHO grade: G2 & G3 *vs* G4	9.496 [7.212–12.503]	**<0.001**	2.622 [1.828–3.760]	**<0.001**
IDH status: WT *vs* Mut	0.117 [0.090–0.152]	**<0.001**	0.305 [0.198–0.470]	**<0.001**
1p/19q codeletion: codel *vs* non-codel	4.428 [2.885–6.799]	**<0.001**	1.467 [0.887–2.428]	0.136
Gender: Female *vs* Male	1.262 [0.988–1.610]	0.062	1.223 [0.934–1.602]	0.144
Race: Asian & Black or African American *vs* White	0.821 [0.502–1.344]	0.433		
Age: <=60 *vs* >60	4.668 [3.598–6.056]	**<0.001**	1.496 [1.096–2.042]	**0.011**
STEAP3: Low *vs* High	5.440 [4.088–7.240]	**<0.001**	1.673 [1.110–2.522]	**0.014**

**Note:**

Bold *P* values are statistically significant (*P* < 0.05).

**Table 3:** Univariate and multivariable Cox regression of STEAP3 expression for progression-free survival in TCGA-GBMLGG cohorts.

Characteristics	Univariate Cox regression		Multivariate Cox regression	
	Hazard ratio (95% CI)	*P* value	Hazard ratio (95% CI)	*P* value
WHO grade: G2 & G3 *vs* G4	6.008 [4.726–7.638]	**<0.001**	1.974 [1.435–2.716]	**<0.001**
IDH status: WT *vs* Mut	0.151 [0.119–0.191]	**<0.001**	0.290 [0.200–0.421]	**<0.001**
1p/19q codeletion: codel *vs* non-codel	3.373 [2.438–4.666]	**<0.001**	1.446 [0.993–2.106]	0.055
Gender: Female *vs* Male	1.083 [0.875–1.342]	0.463		
Race: Asian & Black or African American *vs* White	0.787 [0.515–1.202]	0.267		
Age: <=60 *vs* >60	2.873 [2.268–3.640]	**<0.001**	1.044 [0.781–1.395]	0.772
STEAP3: Low *vs* High	3.708 [2.947–4.666]	**<0.001**	1.450 [1.045–2.013]	**0.026**

**Note:**

Bold *P* values are statistically significant (*P* < 0.05).

**Figure 3:**
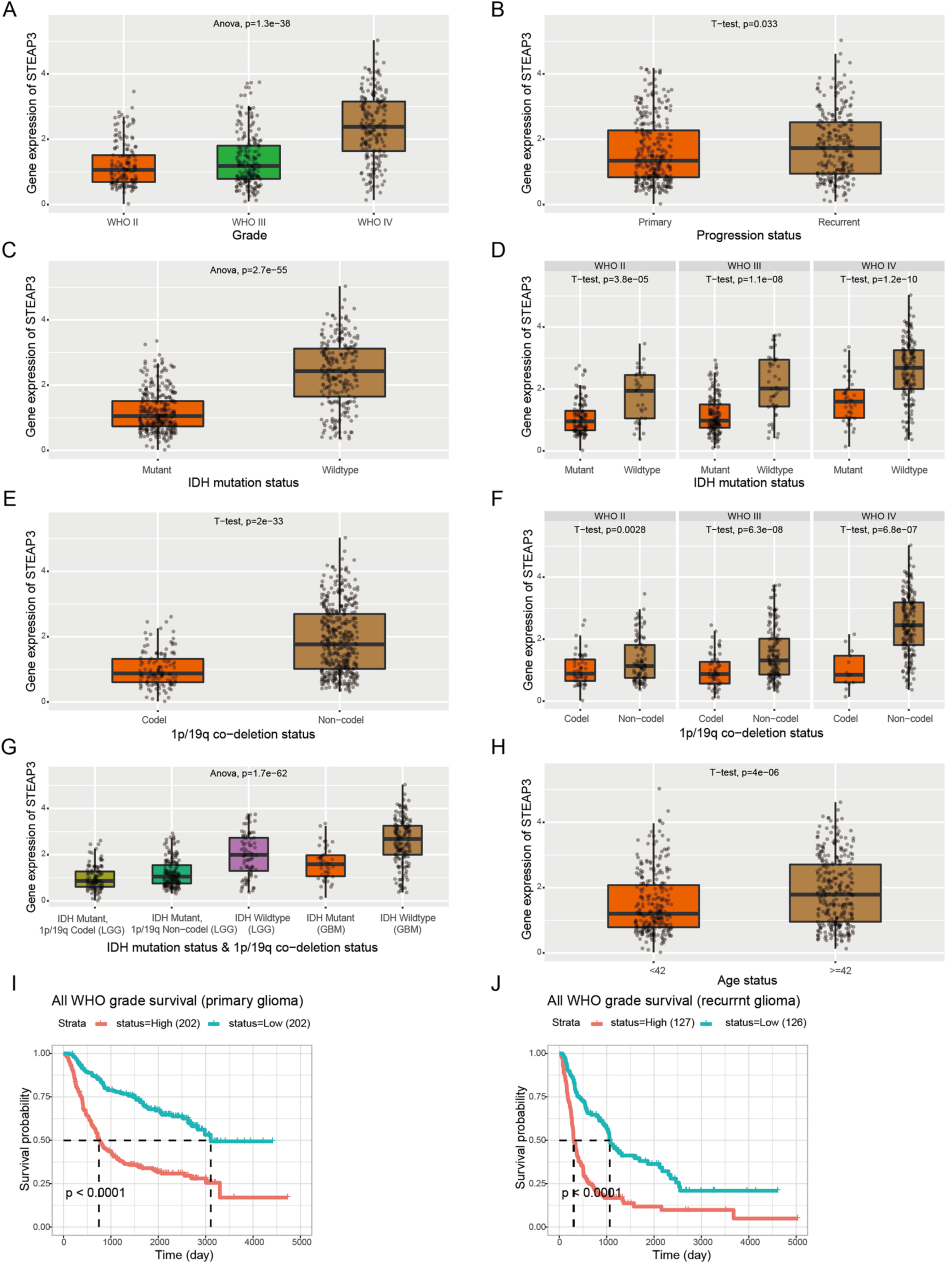
Correlation between STEAP3 expression and clinicopathological characteristics in glioma. (A–J) Correlation of STEAP3 expression level with WHO grade (A), progression status (B), IDH mutation status (C), IDH mutation status based on WHO grade (D), 1p/19q co-deletion status (E), 1p/19q co-deletion status based on WHO grade (F), combination of IDH mutation status and 1p/19q co-deletion status (G), age status (H), and survivals (I and J) from the RNAseq_693 dataset in CGGA database.

Furthermore, we employed the RNAseq_693 dataset from CGGA database to cross-validate the role of STEAP3 in glioma. STEAP3 expression was markedly elevated in high-grade and recurrent glioma patients (Figs. 3A and 3B). IDH mutation and 1p/19q co-deletion are two validated biomarkers for glioma patients. We then explored the association between STEAP3 expression and the status of IDH mutation and 1p/19q co-deletion. As shown in Figs. 3C–3G, we found significantly increased STEAP3 expression in patients with IDH wild-type and 1p/19q non-codeletion, and correlated with WHO grade. In addition, patients older than 42 years harbored higher STEAP3 expression levels (Fig. 3H). Patients with higher STEAP3 expression had worse prognosis than those with lower STEAP3 expression in primary and recurrent gliomas, respectively (Figs. 3I and 3J). These findings further validated the prognostic value of STEAP3 and its correlation with clinicopathological parameters in glioma.

### DNA methylation of STEAP3 and its prognostic value in glioma

**Figure 4:**
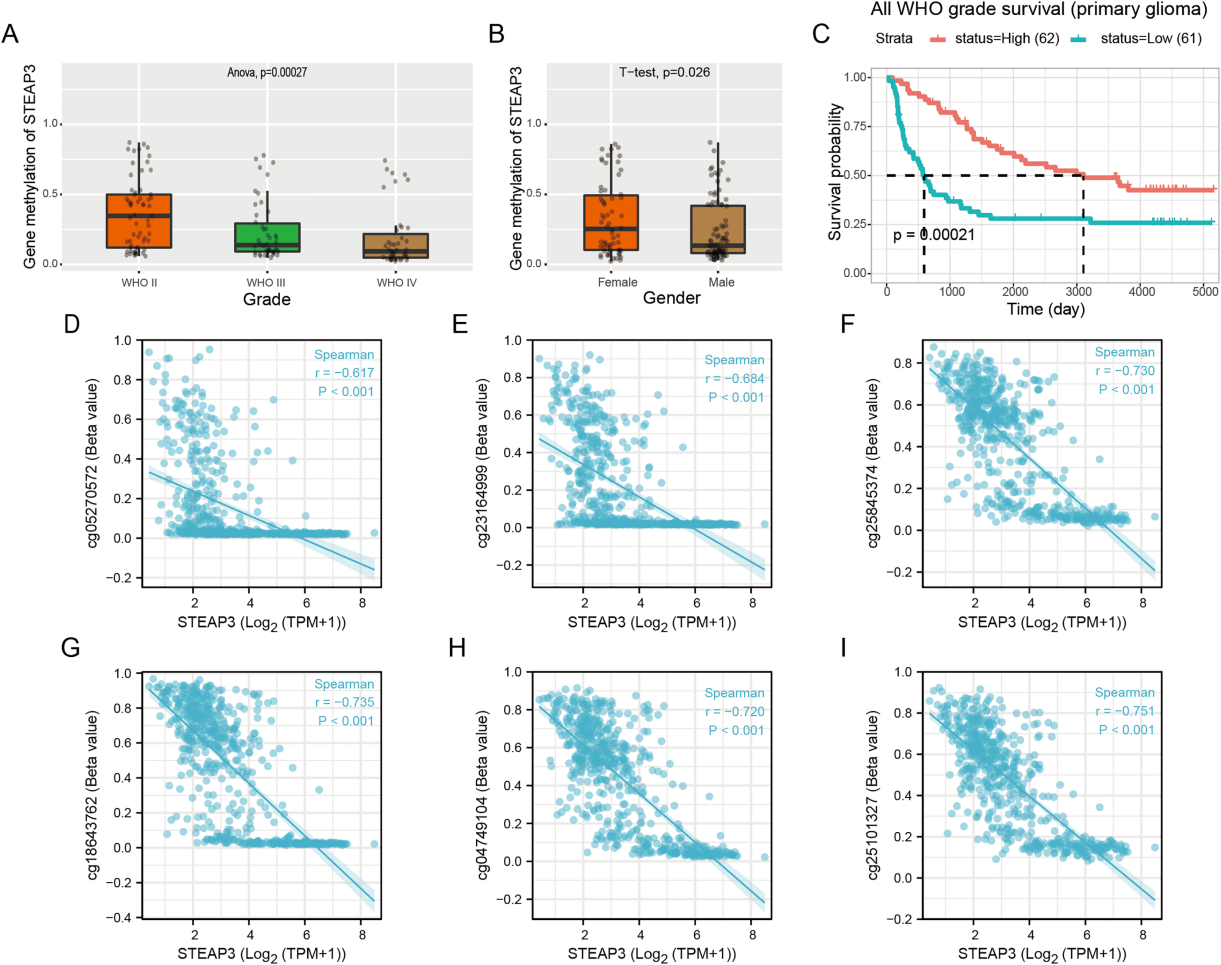
Correlation between STEAP3 methylation and clinicopathological characteristics in glioma. (A–C) Correlation of STEAP3 methylation level with WHO grade (A), gender (B), and survival (C) from the Methyl_159 dataset in CGGA database. (D–I) Correlation between STEAP3 expression and DNA methylation at CpG sites in the STEAP3 gene by the Xiantao database.

Epigenetic changes, such as aberrant DNA methylation, commonly contribute to the development of human tumors, including brain glioma (Russo et al., 2021). We explored the DNA methylation pattern of STEAP3 in the Methyl_159 dataset of CGGA database. The methylation level of STEAP3 was obviously decreased with WHO grade (Fig. 4A). STEAP3 methylation levels were significantly reduced in male patients (Fig. 4B). In primary gliomas, patients with lower STEAP3 methylation level had worse prognosis than those with higher STEAP3 methylation level (Fig. 4C). These results were consistent with STEAP3 expression data. Furthermore, we employed the Xiantao tool to explore the correlation between STEAP3 expression and the degree of DNA methylation at multiple CpG sites. As shown in Figs. 4D–4I, cg05270572, cg23164999, cg25845374, cg18643762, cg04749104, and cg25101327, were significantly negatively correlated with STEAP3 expression in glioma. A gene structure plot illustrating the position of the CpG sites was shown in Fig. S4. These results revealed that STEAP3 promoter methylation was inversely correlated with its gene expression, and might serve as an effective prognostic biomarker for glioma.

### Single-cell functional state atlas of STEAP3 in glioma

**Figure 5:**
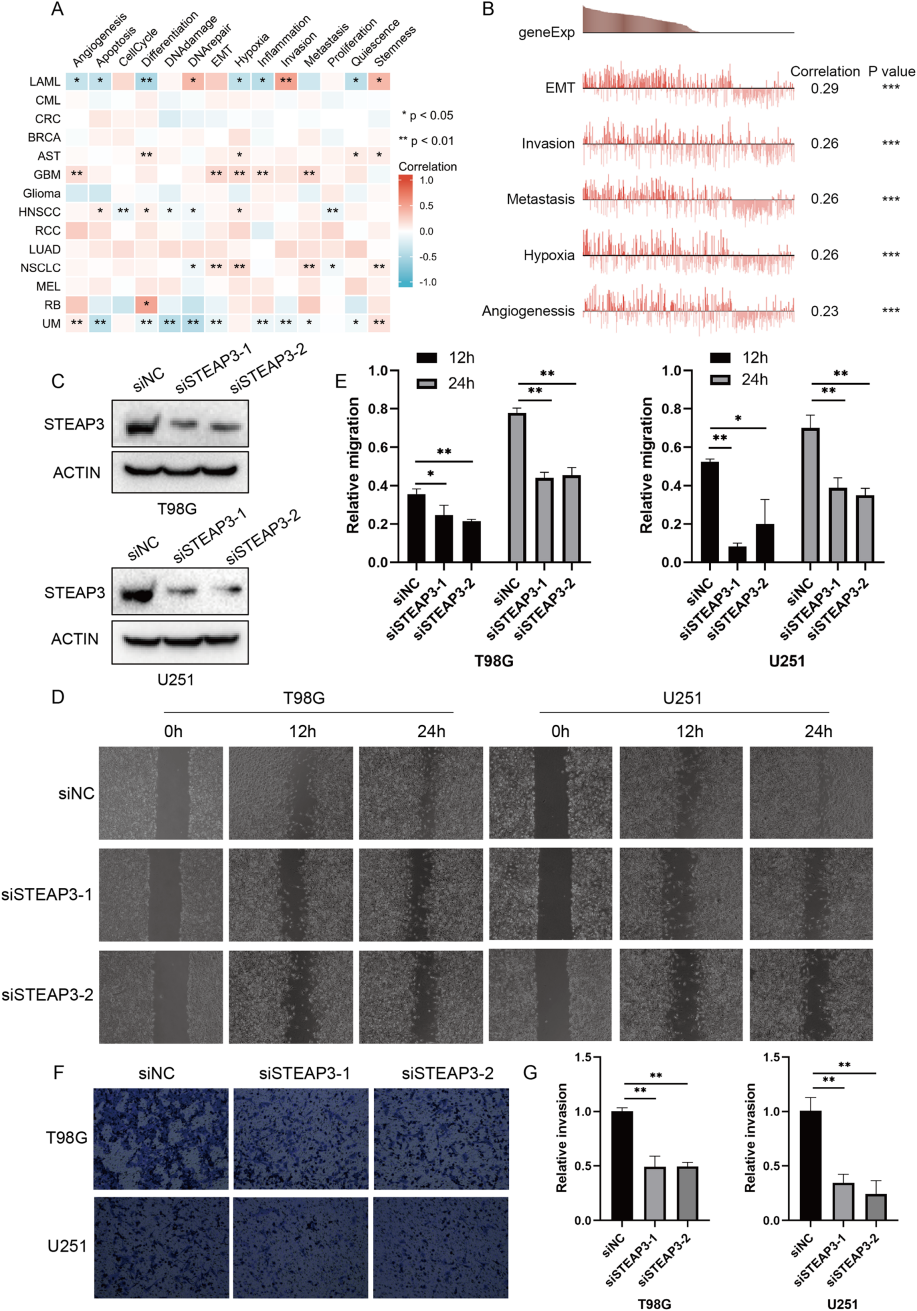
Functional relevance of STEAP3 in glioma. (A) Relevance of STEAP3 across 14 functional states in distinct cancers using the CancerSEA database. Red indicates positive correlation whereas blue represents negative correlation. (B) Functional relevance of STEAP3 in GBM analyzed by the CancerSEA database. (C) Confirmation of siSTEAP3 or siNC-mediated knockdown efficiency of STEAP3 by western blot. (D and E) Scratch assay showed significant migration delay in siSTEAP3 cells at 12, 24 h after transfection compared to siNC cells (T98G and U251). (F and G) Invasion of T98G and U251 cells with siNC or siSTEAP3 transfection was detected through transwell assay. *P* values: * represent *P* < 0.05, ** represent *P* < 0.01, and *** represent *P* < 0.001.

Considering the intra-tumoral heterogeneity, we continued to explore the relevance of STEAP3 expression across 14 functional states in cancers at single-cell resolution. STEAP3 expression was significantly positively associated with epithelial-to-mesenchymal transition (EMT) in GBM, but not all glioma types (Figs. 5A and 5B). These results suggested that STEAP3 might promote the EMT process of GBM, thus facilitating tumor invasion and metastasis. To further confirm the effects of STEAP3 in glioma, wound healing and transwell invasion assays were conducted. Transient transfection of STEAP3 was established by siRNAs and validated by Western Blot (Fig. 5C). The results of wound healing assays revealed that knocking down of STEAP3 could significantly suppress glioma cells migration (Figs. 5D and 5E). Transwell invasion assays revealed that STEAP3 downregulation remarkably inhibit T98G and U251 cells invasion (Figs. 5F and 5G). Namely, these observations indicate that STEAP3 facilitated migration and invasion in glioma cells.

### STEAP3 co‑expression network and pathway enrichment analysis

**Figure 6:**
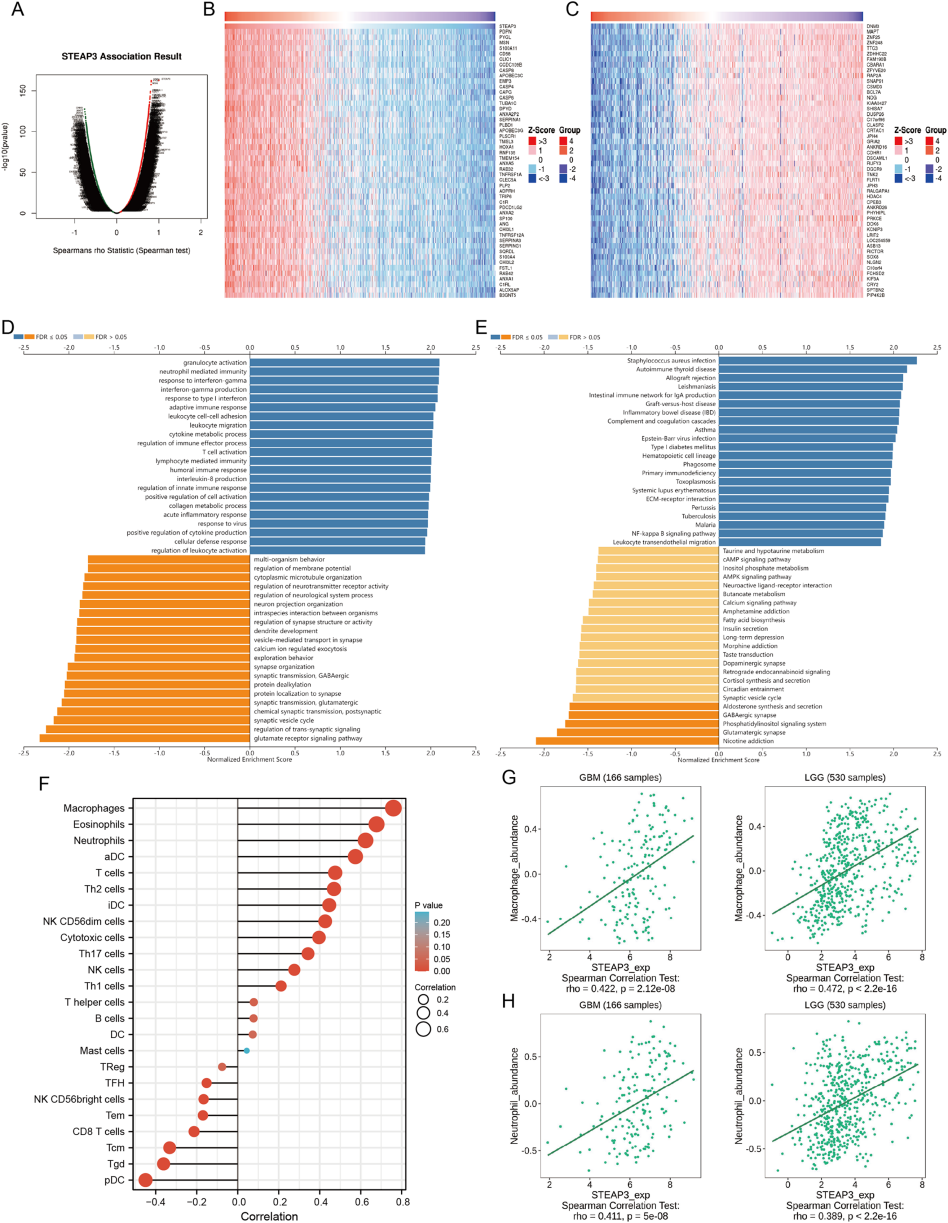
STEAP3 co-expression network and GSEA enrichment analysis. (A) Volcano plot showing the co-expressed profiling of STEAP3 in glioma by Spearman test. (B and C) Heatmaps of the top 50 genes positively (B) and negatively (C) correlated with STEAP3, respectively. (D and E) GO (D) and KEGG (E) pathway analysis of the STEAP3 co-expression genes with the LinkInterpreter module of the LinkedOmics database. (F) The correlation between STEAP3 expression and immune cell infiltration in glioma analyzed by the Xiantao tool. (G and H) Validations of the positive correlation between STEAP3 expression and macrophages (G) and neutrophils (H) in GBM and LGG by the TISIDB database.

To investigate the biological roles of STEAP3 in glioma progression, the STEAP3 co-expression profile in the TCGA-GBMLGG cohort was analyzed using the LinkFinder module of LinkedOmics. As presented in Fig. 6A, 8,702 genes (red dots) were positively associated with STEAP3, and 7,816 genes (green dots) were negatively correlated with STEAP3. Figures 6B and 6C showed the heatmaps of the top 50 genes bearing positive and negative correlations with STEAP3, respectively (Table S1). Additionally, genes positively associated with STEAP3 (*P* < 0.05, gene counts: 8,702) were subjected to functional enrichment analysis using the LinkInterpreter module. Gene Ontology term annotation suggested that genes co-expressed with STEAP3 were significantly enriched in inflammation and immune-associated biological process, such as granulocyte activation, neutrophil mediated immunity, response to interferon-gamma, interferon-gamma production, adaptive immune response, and so on (Fig. 6D). KEGG pathway analysis exhibited that these genes were mainly involved in inflammation and immune-related pathways, such as *Staphylococcus aureus* infection, autoimmune thyroid disease, allograft rejection, leishmaniasis, intestinal immune network for IgA production, *etc*. (Fig. 6E). These findings suggested that STEAP3 co‑expression network may play a role in inflammation and immune regulation in glioma.

### Role of STEAP3 in immune microenvironment of glioma

**Figure 7:**
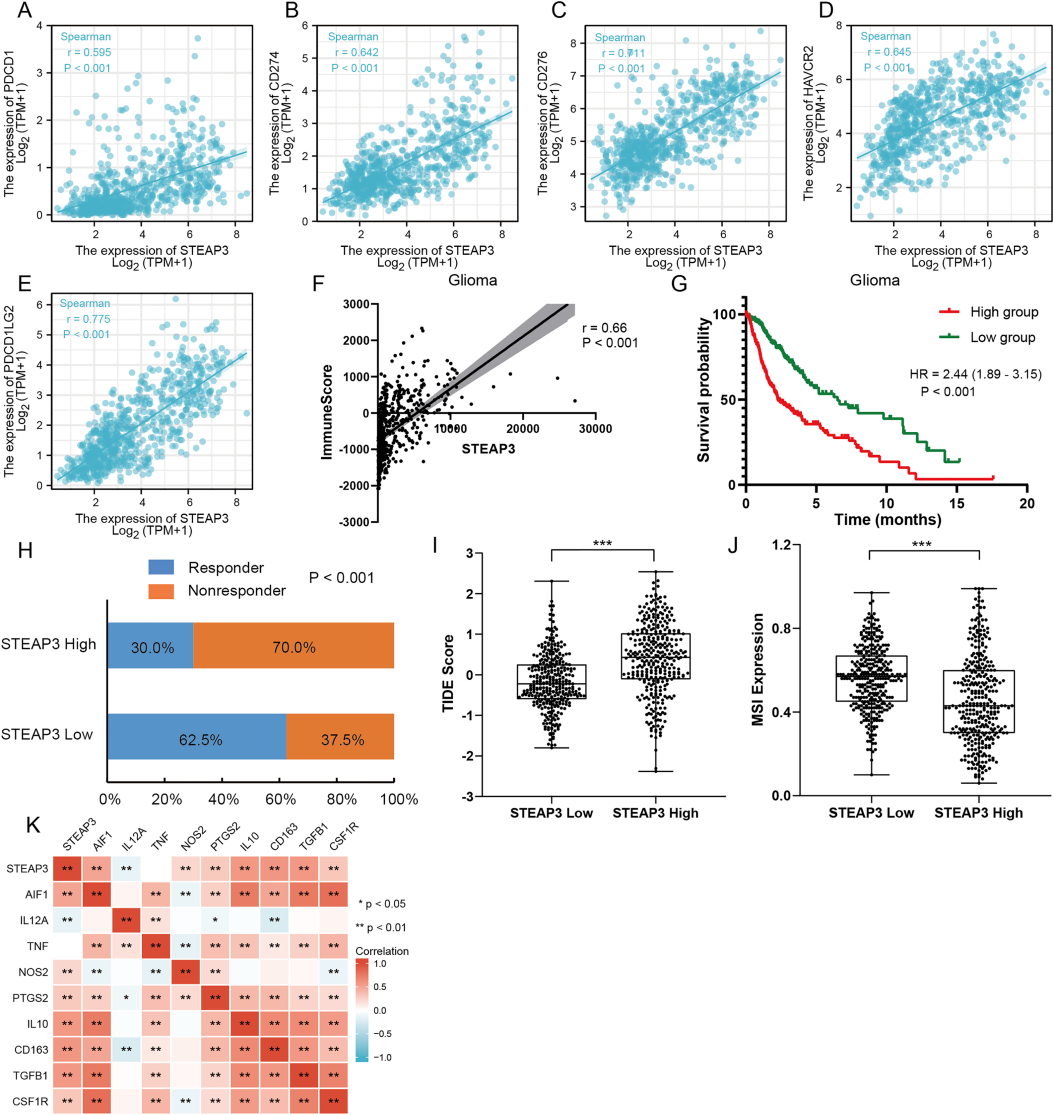
The role of STEAP3 in the immune microenvironment of glioma. (A–E) Correlation of STEAP3 expression and several immune checkpoints, such as PDCD1 (A), CD274 (B), CD276 (C), HAVCR2 (D), PDCD1LG2 (E). (F) Association between STEAP3 expression with immune score in the TCGA glioma dataset calculated by ESTIMATE algorithm. (G) Kaplan–Meier survival curve of overall survival stratified by immune score in the TCGA glioma dataset. (H) Predicted anti-PD1 and anti-CTLA4 response rate for STEAP3 high and low groups in the TCGA glioma dataset. (I and J) TIIDE score (I) and MSI expression (J) between STEAP3 high and low groups. (K) Heatmap of correlation between STEAP3 and classical macrophage phenotype markers. *P* values: * represent *P* < 0.05, ** represent *P* < 0.01, and *** represent *P* < 0.001.

An increasing number of studies have confirmed that ferroptosis plays an important role in regulating tumor immune microenvironment (Wang et al., 2022; Yan et al., 2021). Therefore, we explored the role of STEAP3 in glioma immune microenvironment. As shown in Fig. 6F, STEAP3 expression was positively correlated with the abundance of various immune infiltration cells, such as macrophages, eosinophils, and neutrophils. The association between STEAP3 expression and macrophages was cross-validated in the TCGA-GBM and TCGA-LGG cohort using TISIDB database, respectively, but not in eosinophils (Figs. 6G and 6H). Using the Xiantao tool, we further analyzed the correlations between STEAP3 with immune checkpoints. As shown in Figs. 7A–7E, immune checkpoints were found to have significant positive correlations with STEAP3, while programmed cell death 1 ligand 2 (PDCD1LG2) exhibited the highest correlation coefficient (r = 0.775, *P* < 0.001). In addition, we assessed the relation between immune infiltration score and STEAP3 expression. Figures 7F and 7G showed its positive association and patients with high immune infiltration score had poorer overall survival (OS) in glioma. We then used the TIDE algorithm to predict anti-PD1 and anti-CTLA4 immunotherapy response in glioma patients. As shown in Fig. 7H, individuals with low STEAP3 expression were more likely to respond to immunotherapy than those with high STEAP3 expression. Consistent with this result, the TIDE score was down-regulated in STEAP3 low expression group, and the microsatellite instability (MSI) score was up-regulated in STEAP3 low expression group (Figs. 7I and 7J). These results indicated that STEAP3 might influence the clinical outcome of glioma patients by regulating the tumor immune microenvironment.

### STEAP3 relating with M2 macrophages in glioma

The correlation analysis revealed that STEAP3 expression was significantly positively associated with the abundance of macrophage infiltration. We further explored the associations between STEAP3 expression and classical macrophage phenotype in the TCGA-GBMLGG cohort with Spearman’s rank correlation test, including gene expression of M0 (undifferentiated) marker (AIF1), M1 (anti-tumor) markers (IL12A, TNF, NOS2, PTGS2) and M2 (tumor-promoting) markers (IL10, CD163, TGFB1, CSF1R). As shown in Fig. 7K, STEAP3 had stronger positive correlations with M0 (AIF1) and M2 (IL10, CD163, TGFB1, CSF1R) macrophage markers, but not with M1 (IL12A, TNF, NOS2, PTGS2) markers. These findings suggested that STEAP3 may regulate tumor immune microenvironment by promoting the formation of the M2 macrophages in glioma.

## Discussion

In this study, we aimed to investigate the clinical significance and biological function of STEAP3 in the tumorigenesis and progression of glioma. Compared with normal brain tissue, STEAP3 was highly expressed in glioma and was significantly associated with poor prognosis. Furthermore, multivariate Cox regression analysis indicated that STEAP3 was an independent prognostic factor in glioma. STEAP3 promoter hypomethylation may be the mechanism of upregulation in glioma. Further research revealed that STEAP3 may affect tumor immune response by increasing the infiltration level of M2 macrophages.

Recently, accumulating evidence has revealed that ferroptosis plays an essential role in glioma initiation and progression. RND1 (Rho family GTPase 1), a positive regulator of p53, could induce lipid peroxidation and enhance ferroptosis in GBM (Sun et al., 2022). Xia et al. (2022) identified that apatinib, a small-molecule tyrosine kinase inhibitor, induced ferroptosis in glioma through modulation of the NRF2 pathway, in addition to antiangiogenic and anticancer activities. Additionally, many studies have suggested that ferroptosis induction could be a promising therapeutic strategy (de Souza et al., 2022b; Hu et al., 2020). In a recent study by de Souza et al. (2022a), high levels of NRF2 could reverse temozolomide resistance in glioma *via* ferroptosis induction. Ferroptosis inducers could enhance the antitumor effect of radiation and may serve as effective radiosensitizers that could expand the efficacy and indications of radiation therapy (Ye et al., 2020). However, the link between ferroptosis-related genes and prognosis in glioma patients has been rarely reported. In our study, we explored the prognostic value of ferroptosis-associated gene STEAP3 in glioma, and found that high levels of STEAP3 served as an independent poor prognostic prediction factor in glioma patients.

DNA methylation, a methyl group added to the fifth carbon of the cytosine residue in cytosine-guanine (CpG) dinucleotides, is one of the well-characterized epigenetic mechanisms for regulating gene expression (Xue et al., 2021). Accumulating evidence has suggested that altered DNA methylation patterns are associated with a wide range of age-related diseases, including vascular disease (Lu et al., 2021), Alzheimer’s disease (Altuna et al., 2019), and cancer (Klutstein et al., 2017). DNA methylation patterns could contribute to tumorigenesis and progression by regulating the expression levels of oncogenes and tumor-suppressor genes (Costello et al., 2000; Pathania et al., 2015). According to the analysis of TCGA datasets and IHC staining from HPA database, glioma exhibited significantly high STEAP3 mRNA and protein expression. The mechanisms of STEAP3 upregulation in glioma are currently poorly understood. In our study, the DNA methylation levels of STEAP3 in high-grade glioma were significantly lower than those in low-grade glioma, and the methylation levels of multiple methylated CpG sites were significantly negatively correlated with STEAP3 expression, indicating that low levels of STEAP3 promoter methylation are responsible for the overexpression of STEAP3 in glioma.

The immune microenvironment is composed of various immune cells, cytokines, chemokines, and so on (Huang et al., 2018; Wang et al., 2022). Emerging studies have suggested that glioma has a suppressive immune microenvironment, which further inhibits the response to immunotherapy represented by anti-PD-1/PD-L1 and anti-CTLA4 (Jackson, Choi & Lim, 2019; Lim et al., 2018; Xu et al., 2020). Tumor-associated macrophages (TAMs), instead of T lymphocytes, are the most abundant immune cell populations involved in glioma development (Wei et al., 2019). TAMs in glioma exhibit M2-like macrophage polarization, which largely contributes to the induction of immunosuppressive microenvironment, and further facilitate tumor proliferation, migration, and survival (Hambardzumyan, Gutmann & Kettenmann, 2016; Zhang et al., 2021a). Colony stimulating factor 1 receptor (CSF1R) inhibition could regulate M2 macrophage polarization and attenuate glioma progression (Przystal et al., 2021; Pyonteck et al., 2013). CD163, a membrane protein considered as the most specific M2 macrophage phenotypic marker, predicts poor prognosis in patients with glioma (Liu et al., 2019). In the present study, the findings showed that M2 macrophage markers CD163 and CSF1R were positively correlated with STEAP3 expression in glioma, suggesting that the function of STEAP3 might be related to the regulation of macrophage M2 polarization. Furthermore, our studies revealed that co-expression genes of STEAP3 might participate in inflammation and immune-associated pathways, and STEAP3 expression was positively associated with immune checkpoints. Additionally, immune infiltration analysis showed that individuals with high immune score had a tendency towards the upregulation of STEAP3, which coupled with worse OS. Patients with high STEAP3 expression were associated with low response rates to immunotherapy. Together these results imply that the role of STEAP3 might be involved in the induction of immunosuppressive environment and may be a promising therapeutic target for glioma immunotherapy.

To date, several studies have reported the prognostic prediction potential of STEAP3 in GBM (Chen et al., 2021a; Han et al., 2018; Zhao et al., 2021a). STEAP3-associated prognostic signatures for glioma have also been reported (Guo et al., 2021; Weston et al., 2016). However, existing studies on the biological function and molecular mechanisms of STEAP3 in glioma are few in number. Han et al. (2018) identified that STEAP3 could promote the growth and invasion of glioblastoma, which was consistent with our findings. The possible mechanism by which STEAP3 promotes glioma progression may be through the activation of TfR and the downstream ferritin-STAT3 pathway. In addition, our study indicated that the methylation of STEAP3 promoter region was highly negatively correlated with its expression. Tumor immune infiltrate analysis showed that STEAP3 might influence the clinical outcome of glioma patients by regulating the tumor immune microenvironment, especially the formation of the M2 macrophages. The CancerSEA database showed that STEAP3 may contribute to glioma progression by promoting EMT. However, the mechanism of this effect remains to be further investigated.

In the present study, several limitations still exist. Firstly, gene expression analysis based on retrospective databases needs further investigations in large-scale prospective clinical cohorts to confirm the prognostic value of STEAP3 in glioma. Secondly, it is necessary to conduct functional experiments to elaborate the biological mechanism of STEAP3 and tumor-immune interactions in glioma.

## Conclusion

Taken together, by comprehensively assessing the gene expression profiles, our study provides new insights into the interaction between ferroptosis and glioma immune microenvironment. STEAP3 was up-regulated in glioma, and increased with tumor grade. High STEAP3 expression was recognized as an independent poor prognostic factor. Further study suggested that STEAP3 may contribute to the induction of glioma immunosuppressive microenvironment by regulating macrophage M2 polarization. In summary, STEAP3 has great potential as a prognostic biomarker and therapeutic target in glioma.

## Supplemental Information

10.7717/peerj.15136/supp-1 Supplemental Information 1 Flowchart showing the research processes to identify STEAP3 in this study.

10.7717/peerj.15136/supp-2 Supplemental Information 2 The immunohistochemical analysis of STEAP3 in HNSC and corresponding normal tissues using the HPA database.

10.7717/peerj.15136/supp-3 Supplemental Information 3 The prognostic value of STEAP3 in glioma. (A-B) Kaplan-Meier survival curves for overall survival, disease-specific survival, and progression free survival of STEAP3 in GBM (A) and LGG (B).

10.7717/peerj.15136/supp-4 Supplemental Information 4 Gene structure plot illustrating the methylation position of CpG sites in the STEAP3 promoter. Transcription start site (TSS) is usually marked as +1, the position of the first base upstream is −1, the position of the first base d

10.7717/peerj.15136/supp-5 Supplemental Information 5 The top 50 genes positively and negatively correlated with STEAP3 in LinkedOmics.

10.7717/peerj.15136/supp-6 Supplemental Information 6 Raw data for Figure 1. Expression level of STEAP3 in pan-cancer.

10.7717/peerj.15136/supp-7 Supplemental Information 7 Raw data for Figure 2. Prognostic value of STEAP3 in glioma, LIHC, LUAD, and UCEC.

10.7717/peerj.15136/supp-8 Supplemental Information 8 Raw data for Figure 3. Correlation between STEAP3 expression and clinicopathological characteristics in glioma.

10.7717/peerj.15136/supp-9 Supplemental Information 9 Raw data for Figure 4. Correlation between STEAP3 methylation and clinicopathological characteristics in glioma.

10.7717/peerj.15136/supp-10 Supplemental Information 10 Raw data for Figure 5. Functional relevance of STEAP3 in glioma.

10.7717/peerj.15136/supp-11 Supplemental Information 11 Raw data for Figure 6. STEAP3 co-expression network and GSEA enrichment analysis.

10.7717/peerj.15136/supp-12 Supplemental Information 12 Raw data for Figure 7. The role of STEAP3 in the immune microenvironment of glioma.

10.7717/peerj.15136/supp-13 Supplemental Information 13 Raw data for Figures S2–S3. The immunohistochemical analysis and prognostic value of STEAP3 in tumor.

10.7717/peerj.15136/supp-14 Supplemental Information 14 Raw data for Table 2. Univariate and multivariable Cox regression of STEAP3 expression for overall survival in TCGA-GBMLGG cohorts.

10.7717/peerj.15136/supp-15 Supplemental Information 15 Raw data for Table 3. Univariate and multivariable Cox regression of STEAP3 expression for progression-free survival in TCGA-GBMLGG cohorts.

## Abbreviations

ACC: adrenocortical carcinoma
BLCA: bladder urothelial carcinoma
BRCA: breast invasive carcinoma
CESC: cervical squamous cell carcinoma and endocervical adenocarcinoma
CHOL: cholangiocarcinoma
COAD: colon adenocarcinoma
DLBC: diffuse large B-cell lymphoma
ESCA: esophageal carcinoma
GBM: glioblastoma multiforme
HNSC: head and neck squamous cell carcinoma
KICH: kidney chromophobe
KIRC: kidney renal clear cell carcinoma
KIRP: kidney renal papillary cell carcinoma
LAML: acute myeloid leukemia
LGG: brain lower grade glioma
LIHC: liver hepatocellular carcinoma
LUAD: lung adenocarcinoma
LUSC: lung squamous cell carcinoma
MESO: mesothelioma
OV: ovarian serous cystadenocarcinoma
PAAD: pancreatic adenocarcinoma
PCPG: pheochromocytoma and paraganglioma
PRAD: prostate adenocarcinoma
READ: rectum adenocarcinoma
SARC: sarcoma
SKCM: skin cutaneous melanoma
STAD: stomach adenocarcinoma
TGCT: testicular germ cell tumors
THCA: thyroid carcinoma
THYM: thymoma
UCEC: uterine *corpus* endometrial carcinoma
UCS: uterine carcinosarcoma
UVM: uveal melanoma

## Additional Information and Declarations

### Data Availability

The following information was supplied regarding data availability: The raw data is available in the Supplemental Files.
