# Supplementary figures and images for: High expression of six-transmembrane epithelial antigen of prostate 3 promotes the migration and invasion and predicts unfavorable prognosis in glioma
Source: PeerJ. 2023 Mar 28;11:e15136. doi: 10.7717/peerj.15136 (PMC10065001; doi:10.7717/peerj.15136)

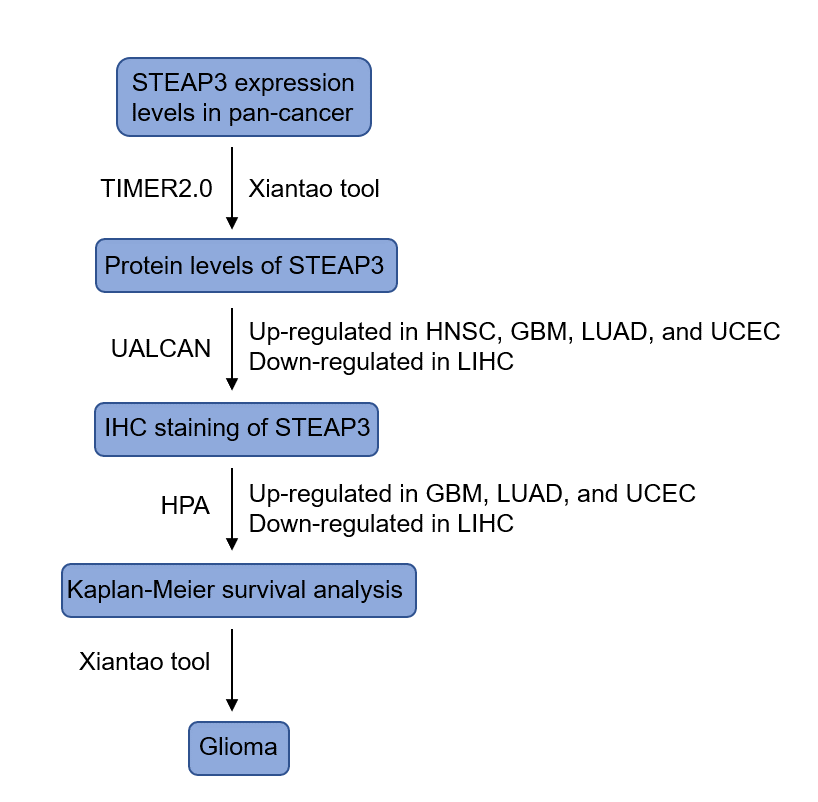

Supplement: Supplemental Information 1 [file peerj-11-15136-s001.png]

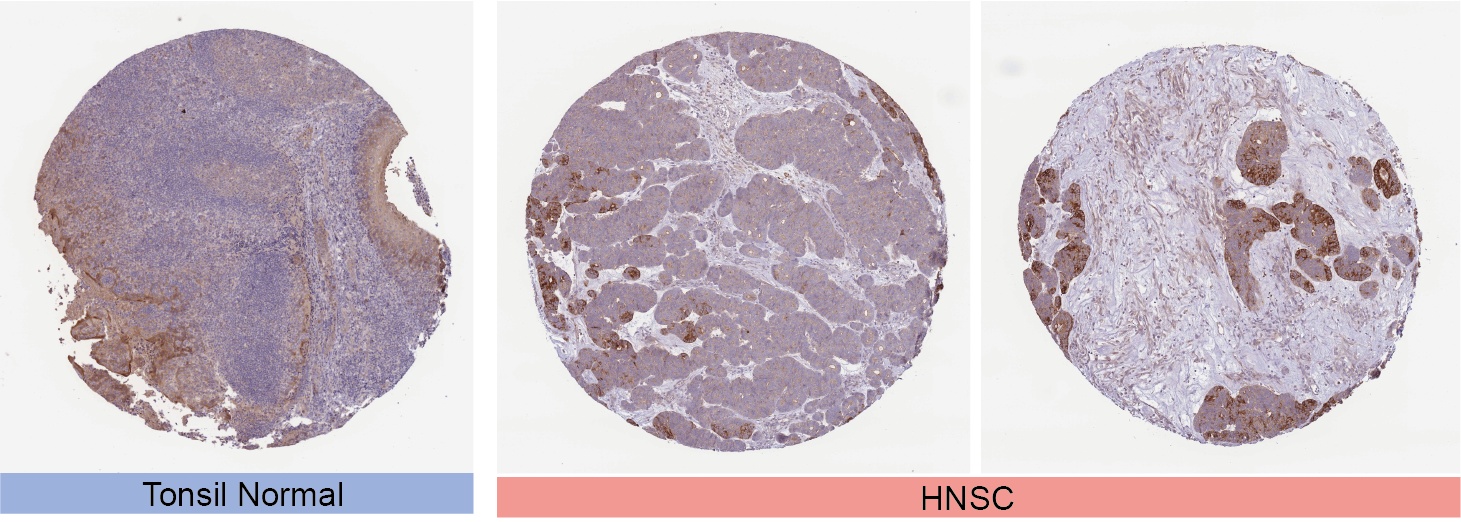

Supplement: Supplemental Information 2 [file peerj-11-15136-s002.png]

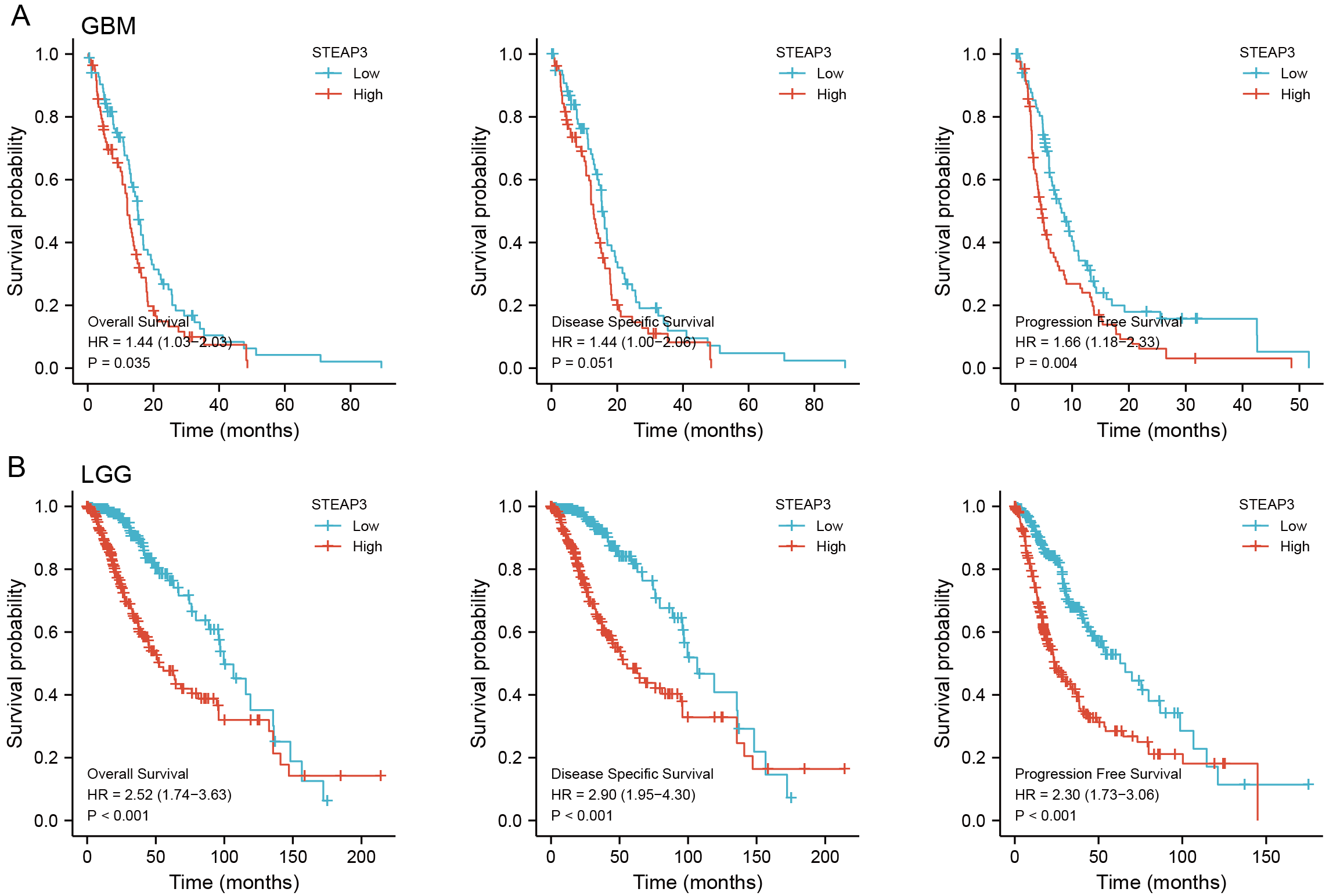

Supplement: Supplemental Information 3 — (A-B) Kaplan-Meier survival curves for overall survival, disease-specific survival, and progression free survival of STEAP3 in GBM (A) and LGG (B). [file peerj-11-15136-s003.png]

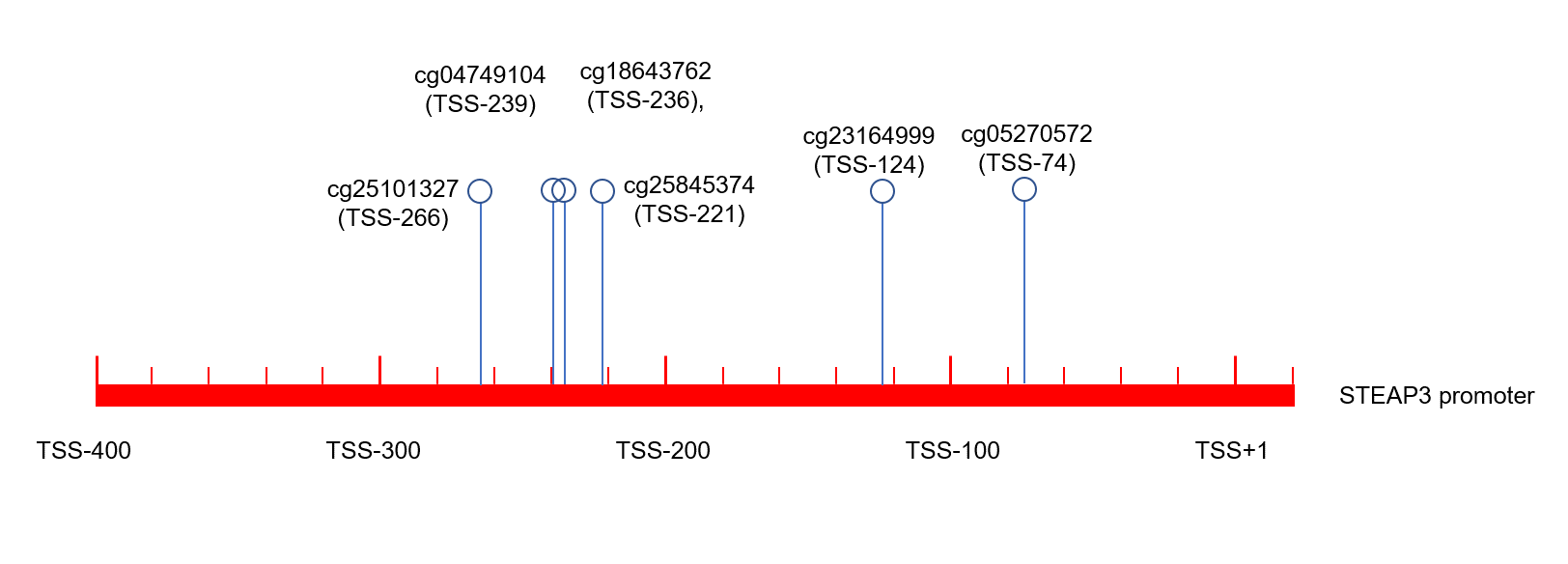

Supplement: Supplemental Information 4 — Transcription start site (TSS) is usually marked as +1, the position of the first base upstream is −1, the position of the first base d [file peerj-11-15136-s004.png]

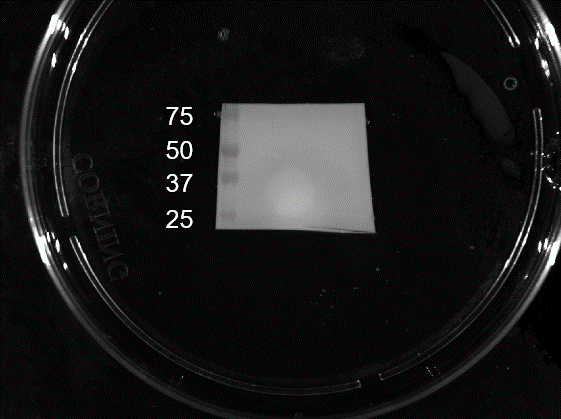

Supplement: Supplemental Information 10 — Functional relevance of STEAP3 in glioma. [file peerj-11-15136-s010.zip › raw data for Figure 5/Raw data for Figure 5C (WB)/T98G-ACTIN (white).png]

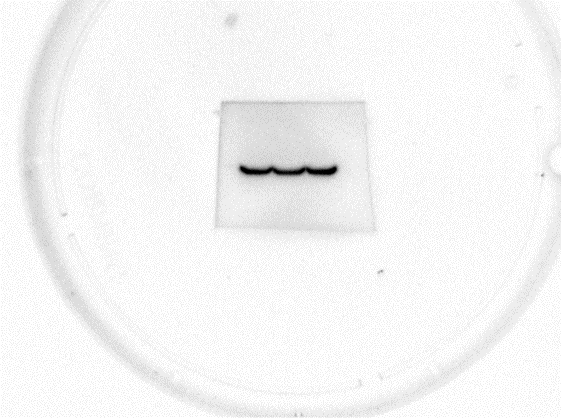

Supplement: Supplemental Information 10 — Functional relevance of STEAP3 in glioma. [file peerj-11-15136-s010.zip › raw data for Figure 5/Raw data for Figure 5C (WB)/T98G-ACTIN.png]

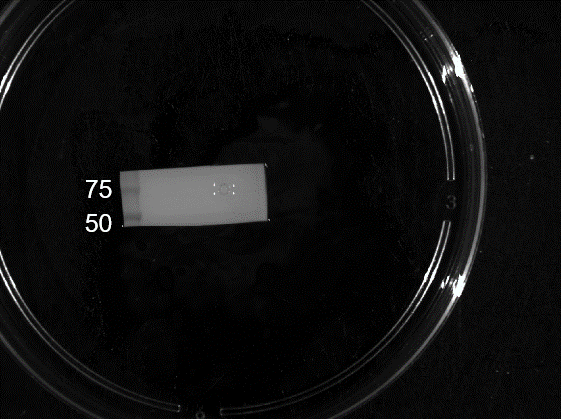

Supplement: Supplemental Information 10 — Functional relevance of STEAP3 in glioma. [file peerj-11-15136-s010.zip › raw data for Figure 5/Raw data for Figure 5C (WB)/T98G-STEAP3 (white).png]

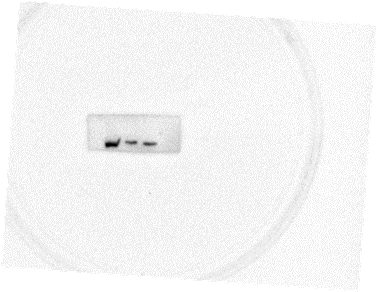

Supplement: Supplemental Information 10 — Functional relevance of STEAP3 in glioma. [file peerj-11-15136-s010.zip › raw data for Figure 5/Raw data for Figure 5C (WB)/T98G-STEAP3.png]

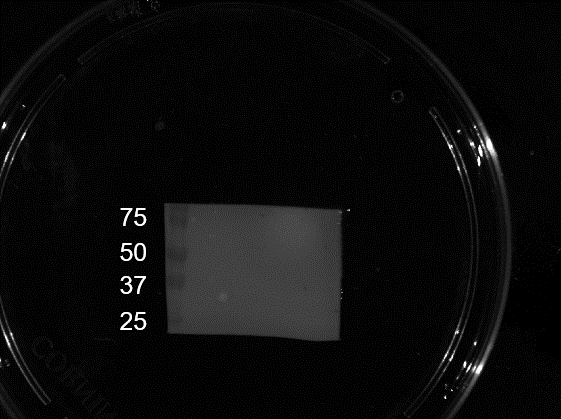

Supplement: Supplemental Information 10 — Functional relevance of STEAP3 in glioma. [file peerj-11-15136-s010.zip › raw data for Figure 5/Raw data for Figure 5C (WB)/U251-ACTIN (white).png]

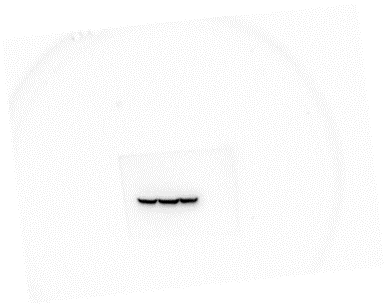

Supplement: Supplemental Information 10 — Functional relevance of STEAP3 in glioma. [file peerj-11-15136-s010.zip › raw data for Figure 5/Raw data for Figure 5C (WB)/U251-ACTIN.png]

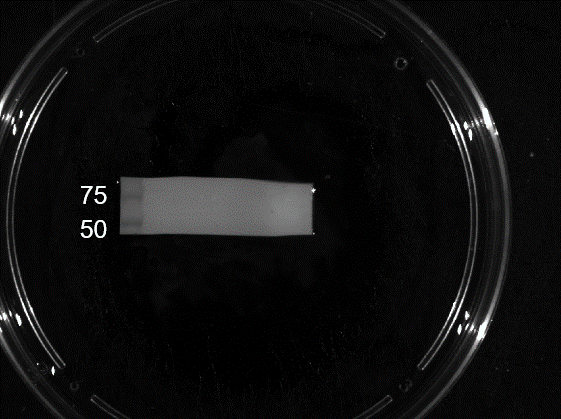

Supplement: Supplemental Information 10 — Functional relevance of STEAP3 in glioma. [file peerj-11-15136-s010.zip › raw data for Figure 5/Raw data for Figure 5C (WB)/U251-STEAP3 (white).png]

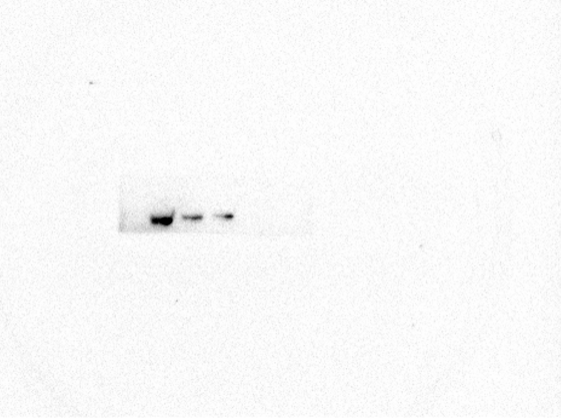

Supplement: Supplemental Information 10 — Functional relevance of STEAP3 in glioma. [file peerj-11-15136-s010.zip › raw data for Figure 5/Raw data for Figure 5C (WB)/U251-STEAP3.tif]

Figure 7A:


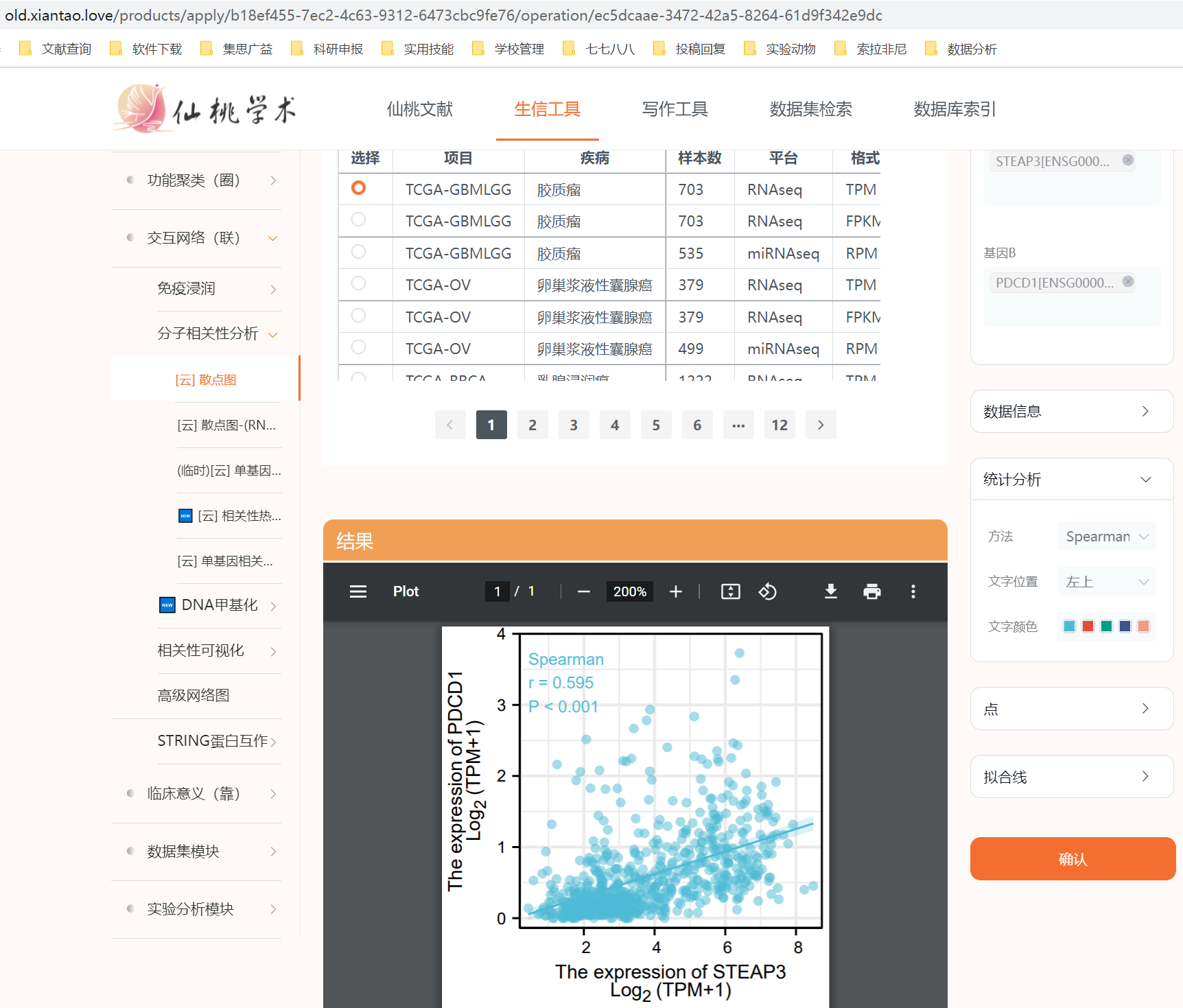


Figure 7B:


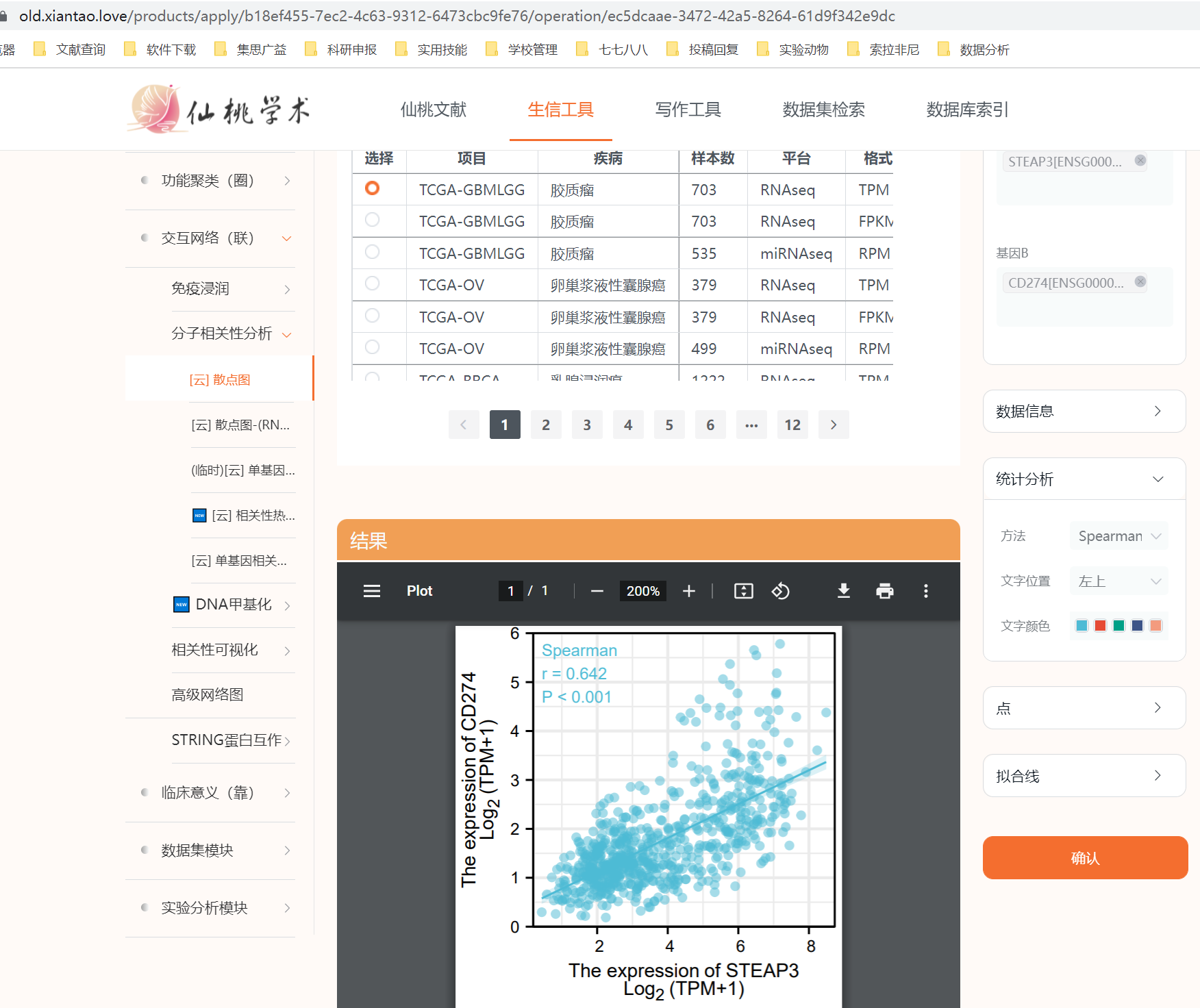


Figure 7C:


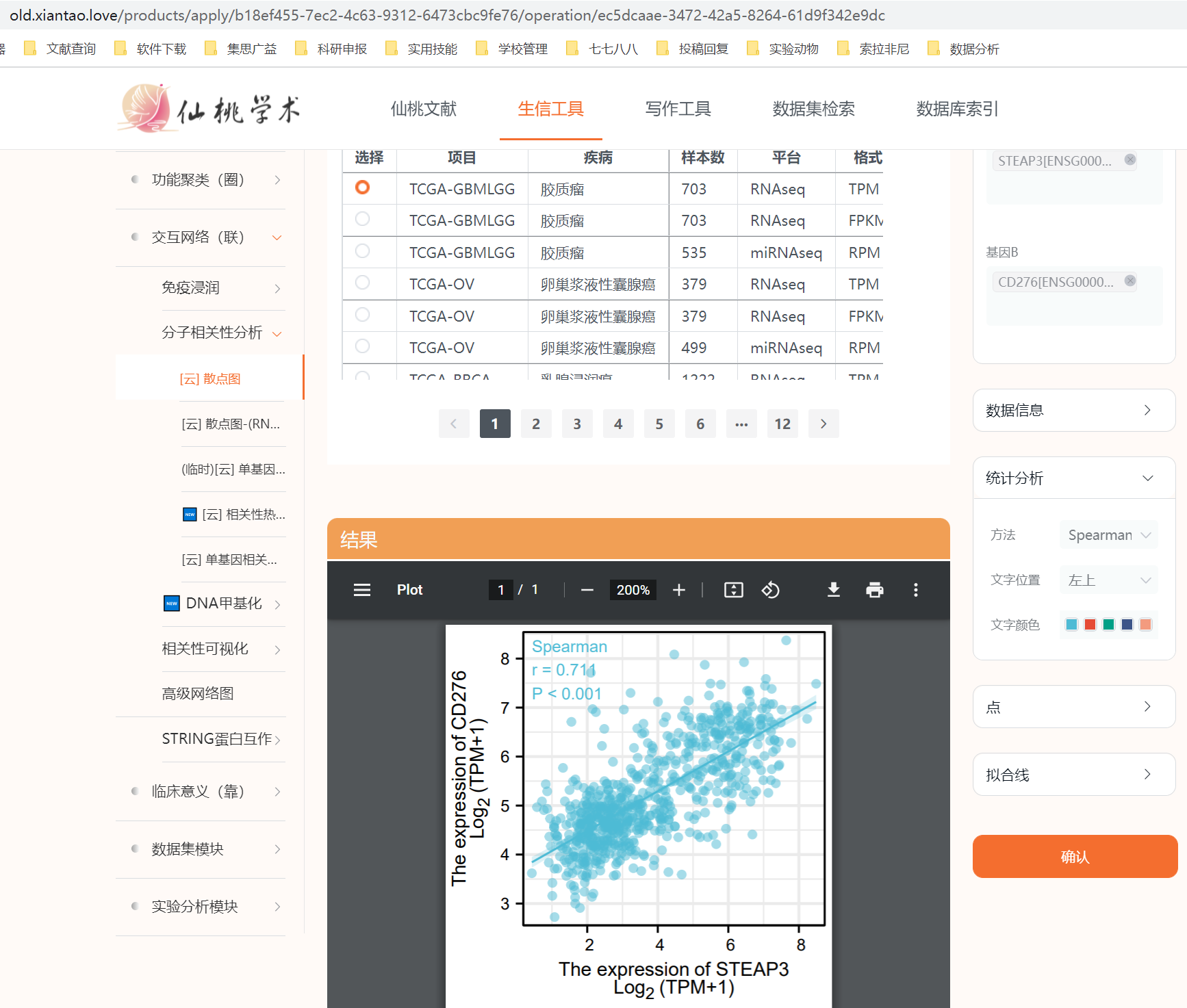


Figure 7D:


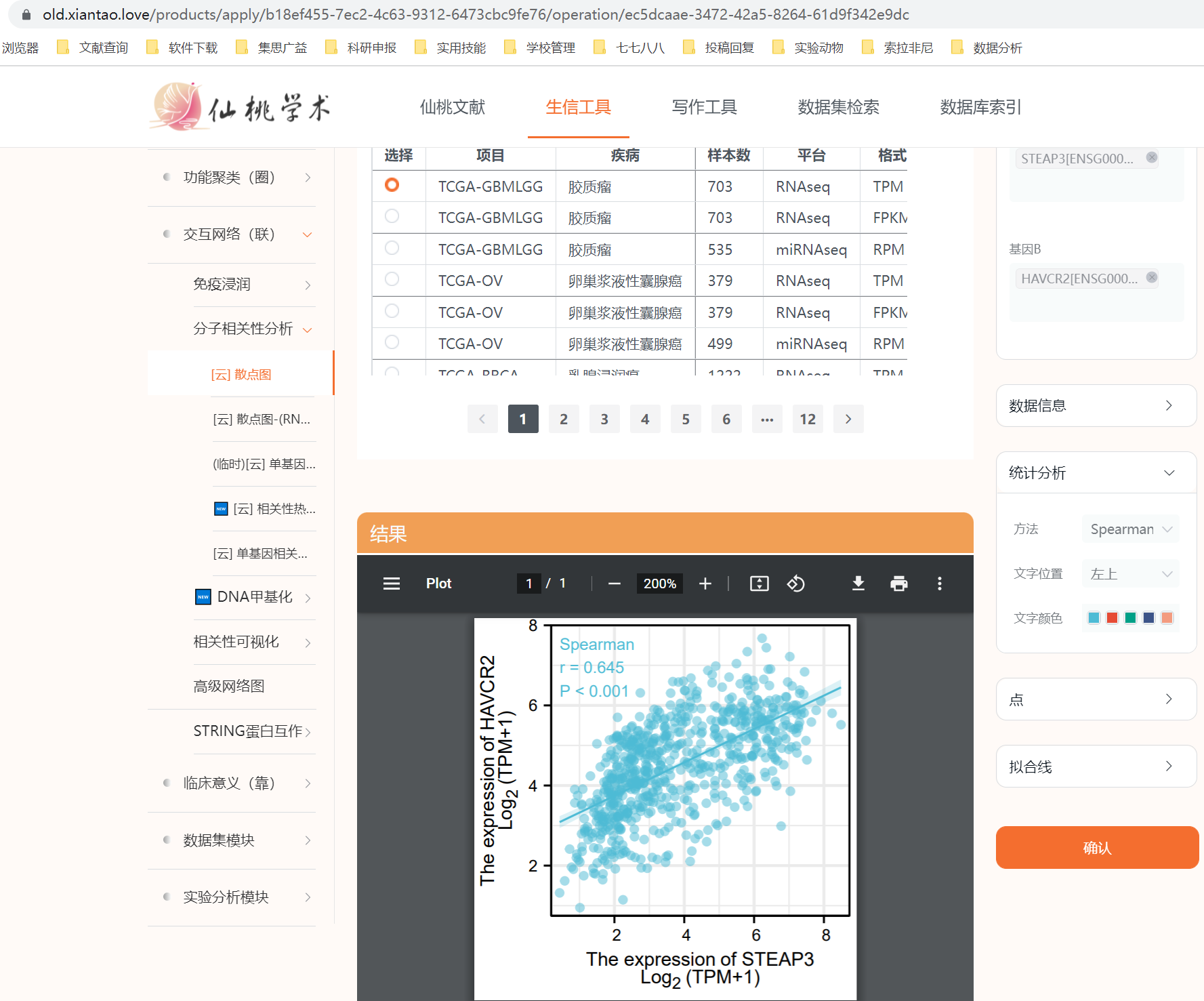


Figure 7E:


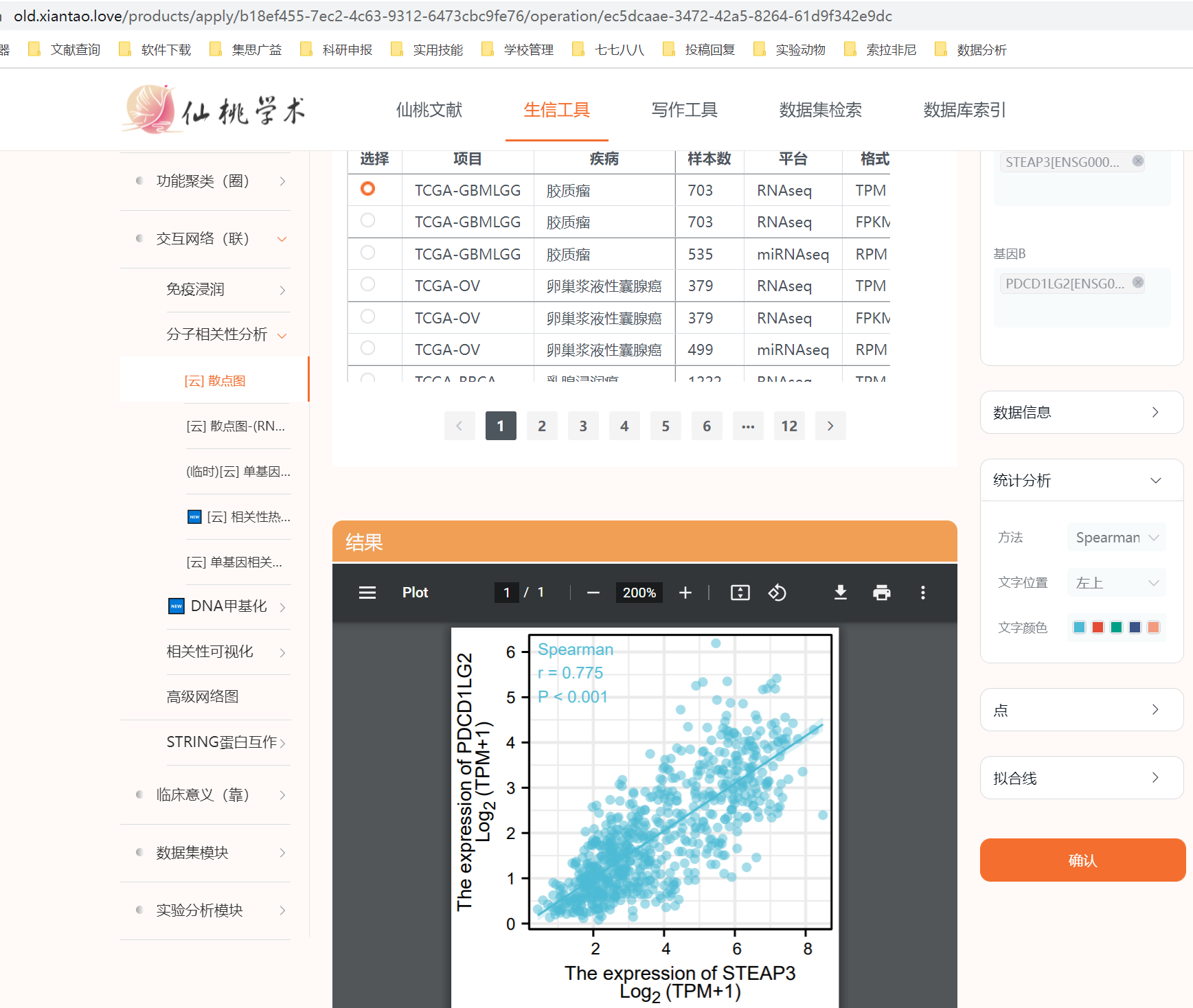


Figure 7H:


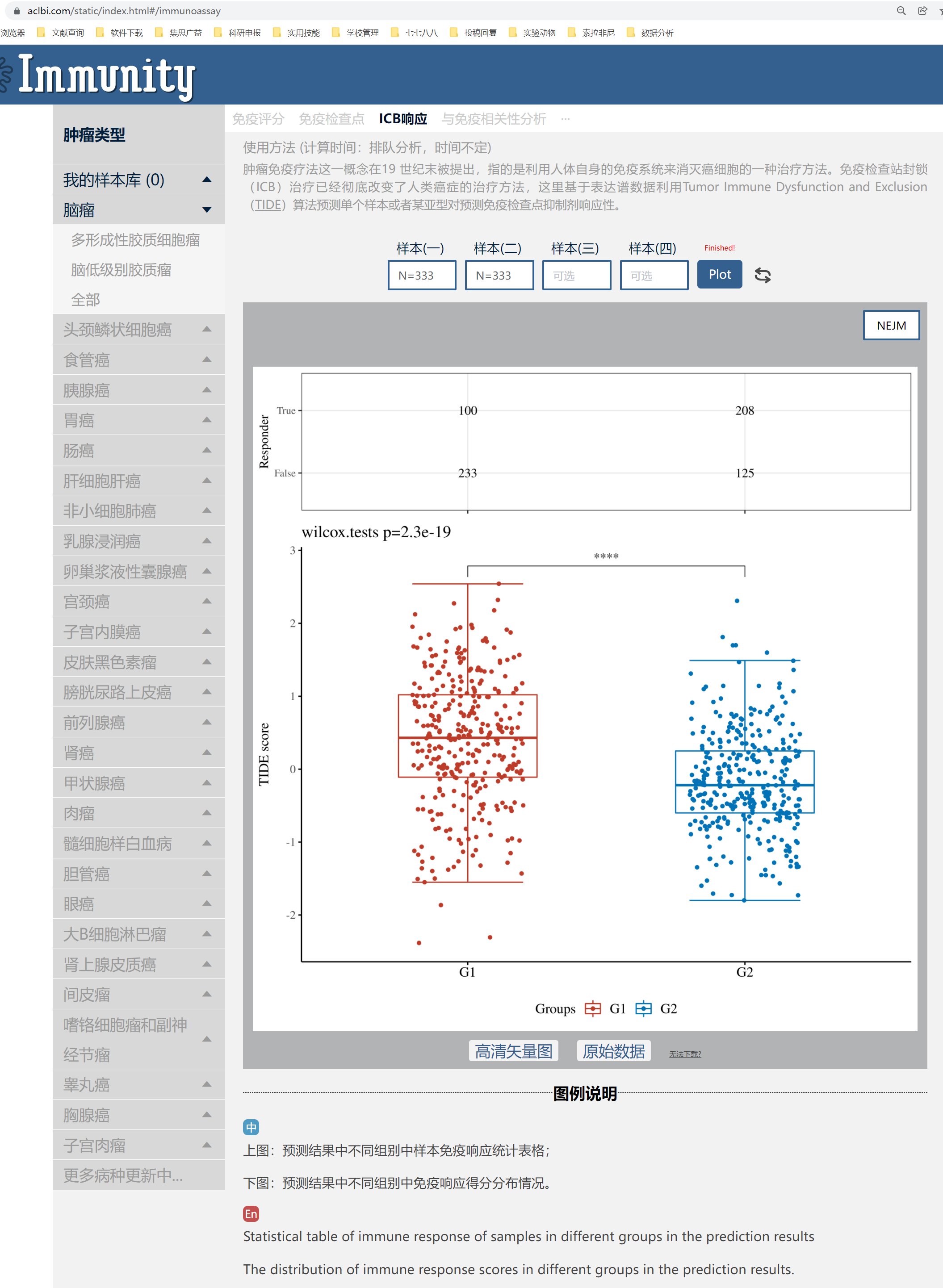

Supplement: Supplemental Information 12 — The role of STEAP3 in the immune microenvironment of glioma. [file peerj-11-15136-s012.zip › raw data for Figure 7/Raw data for Figure 7A-E+Figure 7H.docx]

Table 2


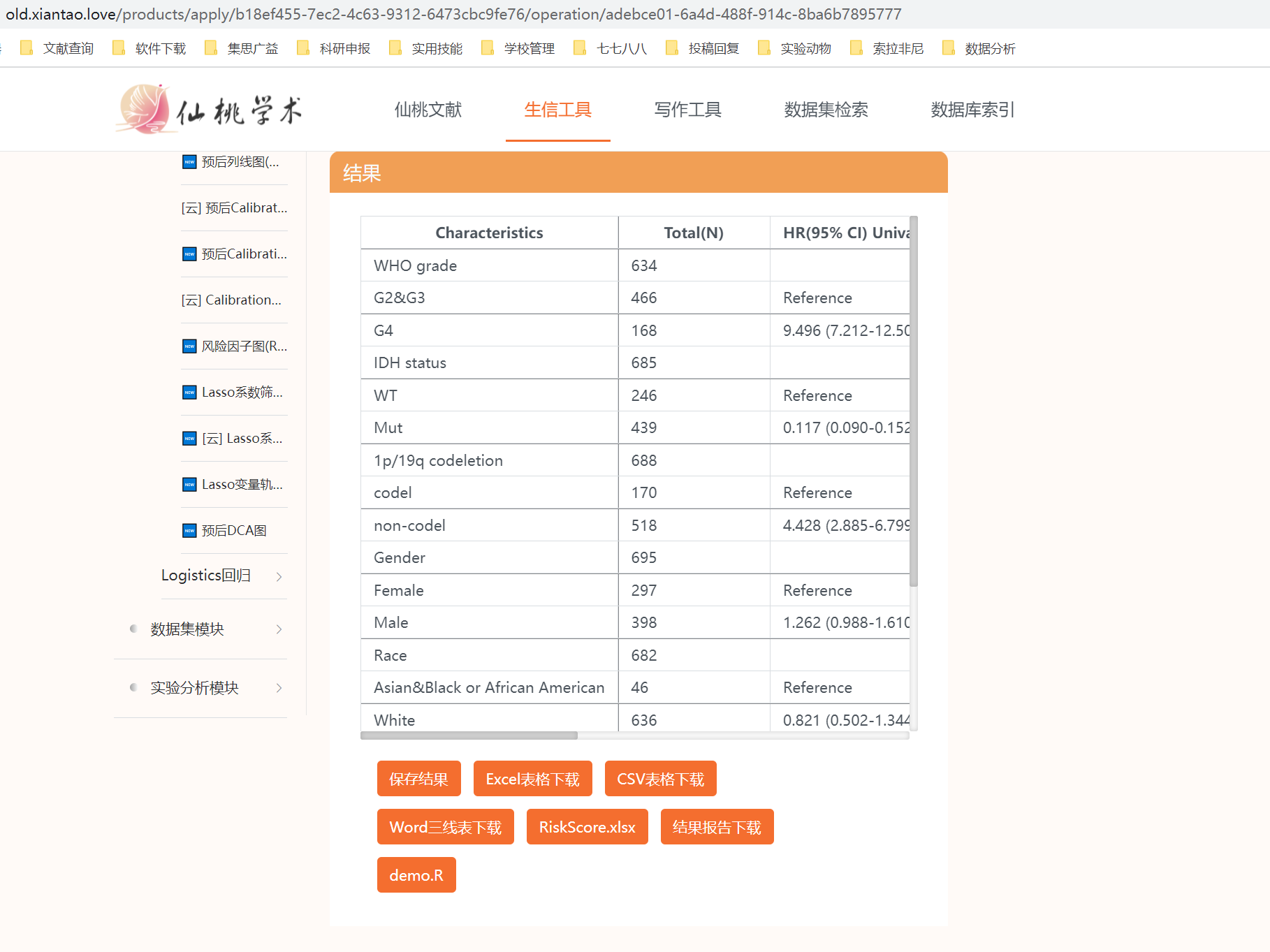

Supplement: Supplemental Information 14 — Univariate and multivariable Cox regression of STEAP3 expression for overall survival in TCGA-GBMLGG cohorts. [file peerj-11-15136-s014.zip › raw data for Table 2/Raw data for Table 2.docx]

Table 3


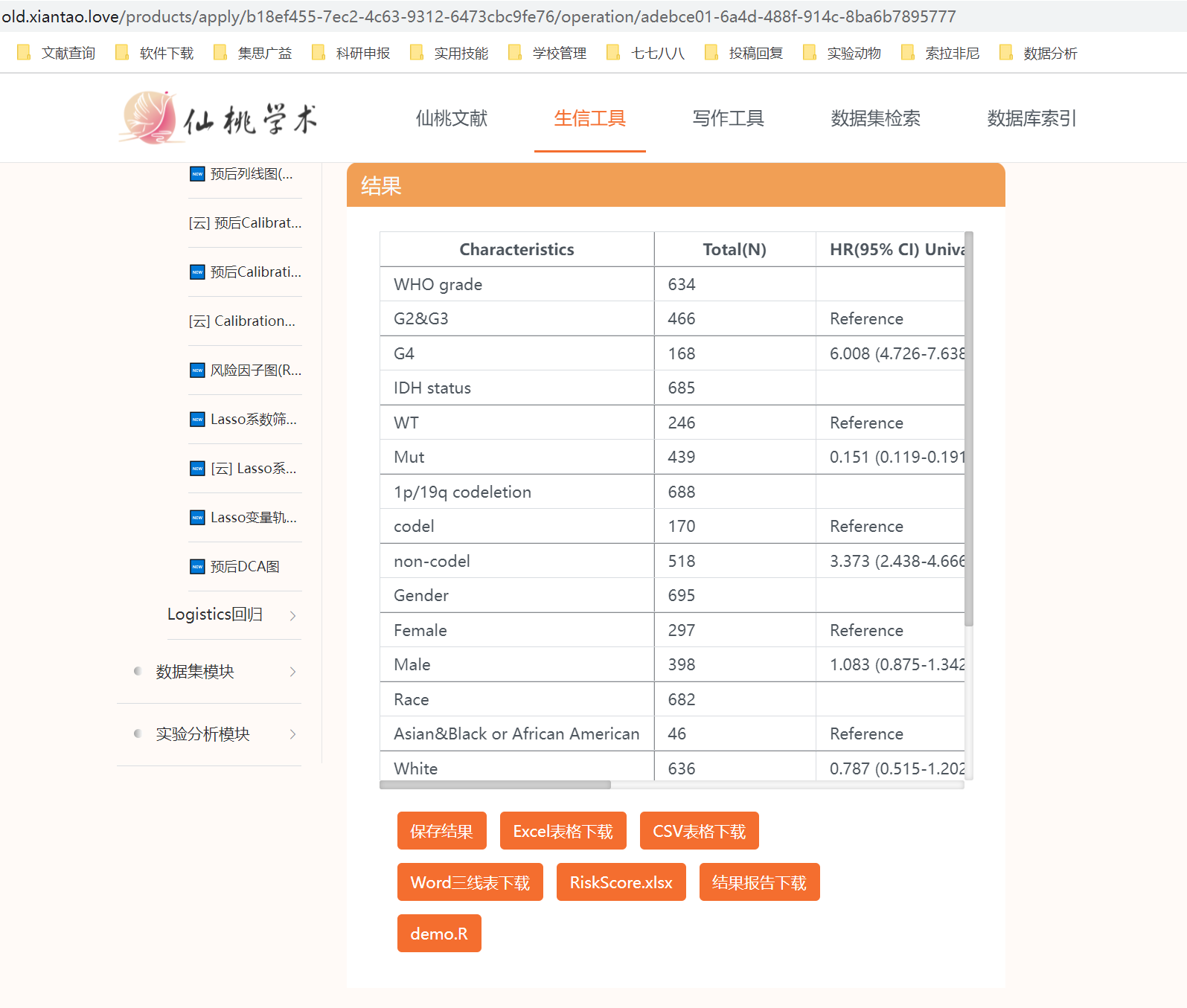

Supplement: Supplemental Information 15 — Univariate and multivariable Cox regression of STEAP3 expression for progression-free survival in TCGA-GBMLGG cohorts. [file peerj-11-15136-s015.zip › raw data for Table 3/Raw data for Table 3.docx]
