# Supplementary material for: High expression of six-transmembrane epithelial antigen of prostate 3 promotes the migration and invasion and predicts unfavorable prognosis in glioma
Source: PeerJ. 2023 Mar 28;11:e15136. doi: 10.7717/peerj.15136 (PMC10065001; doi:10.7717/peerj.15136)
Supplement: Supplemental Information 6 — Expression level of STEAP3 in pan-cancer. [file peerj-11-15136-s006.zip › raw data for Figure 1/Raw data for Figure 1A-D.docx]

Figure 1A:


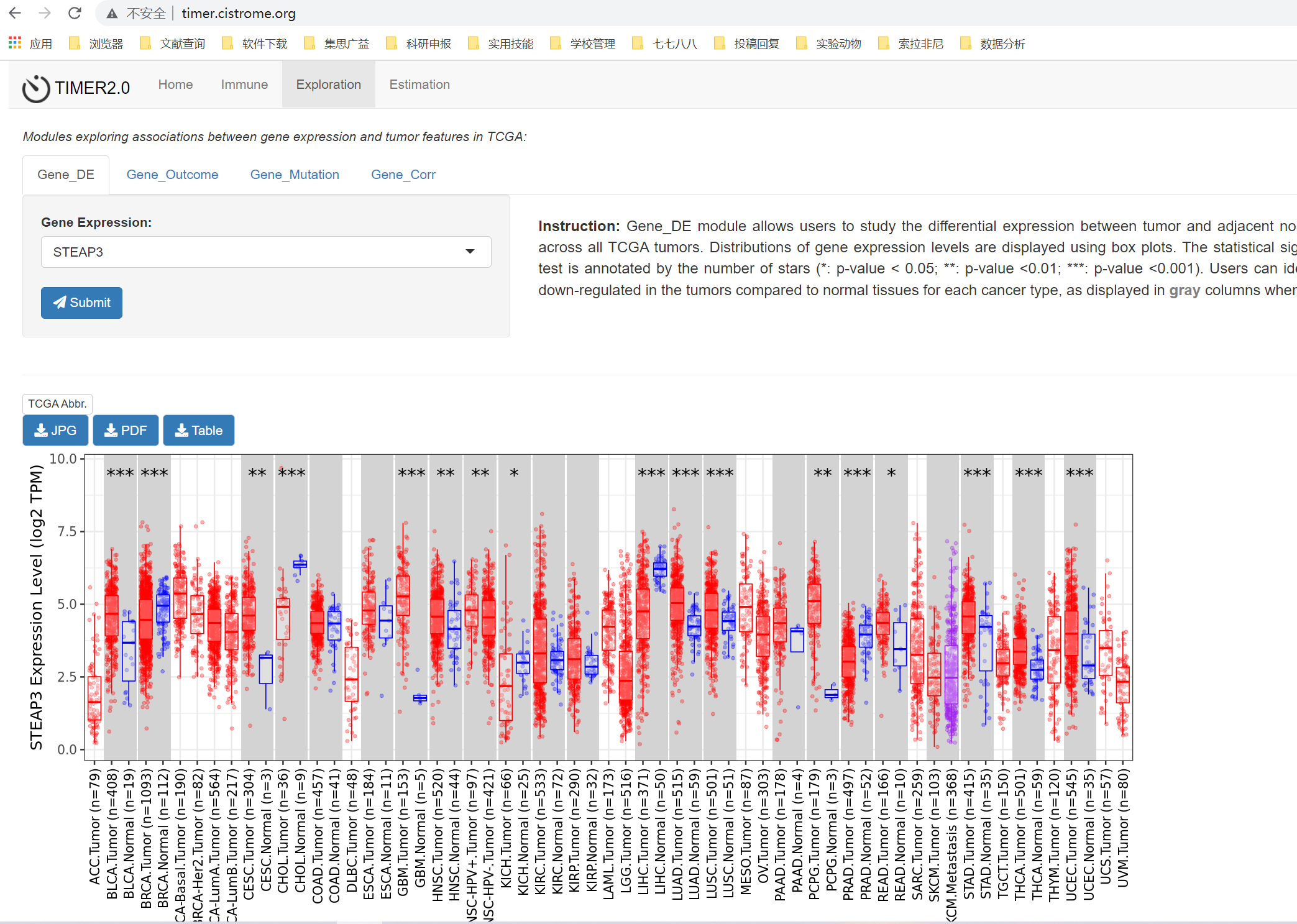


Figure 1B:


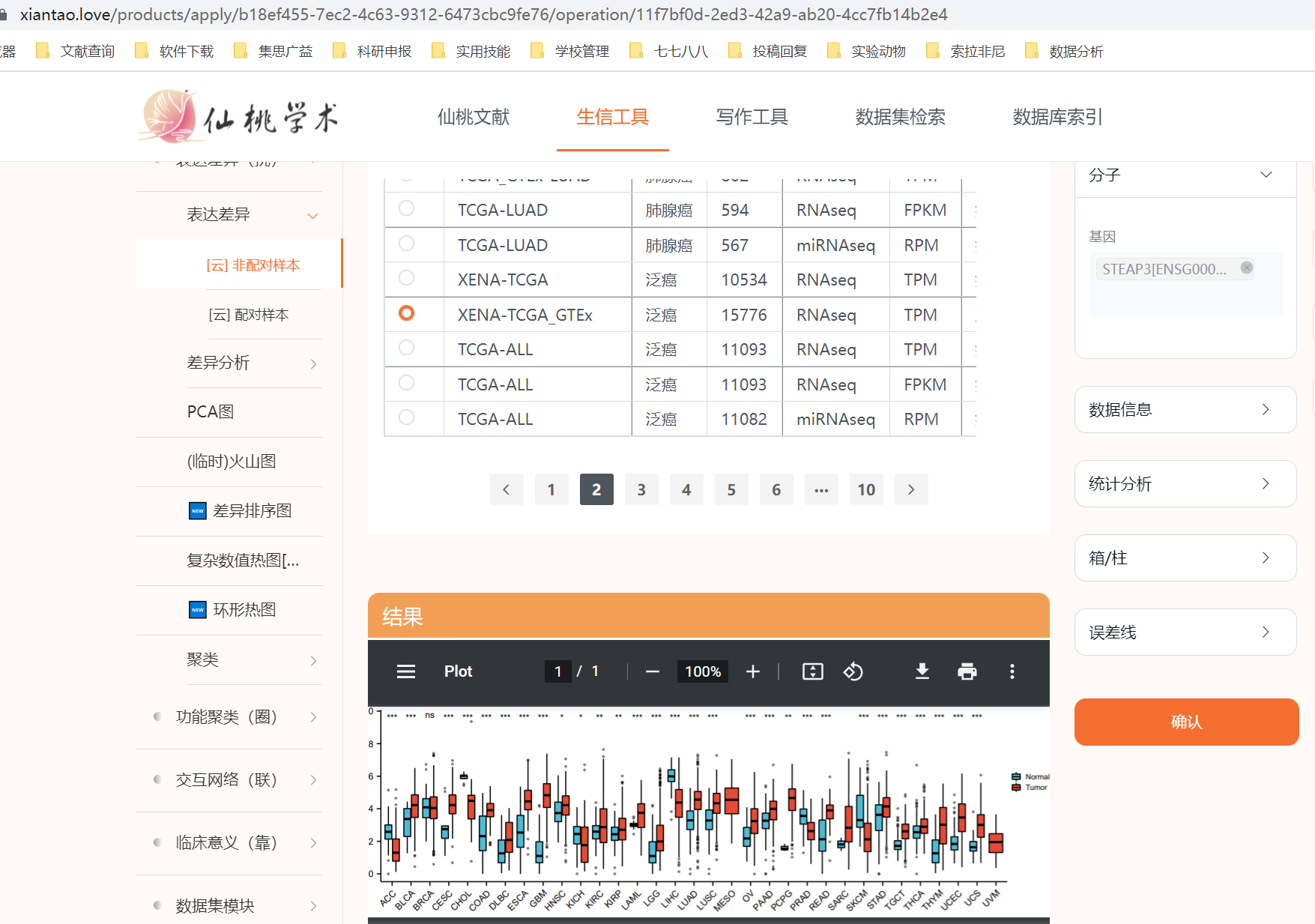


Figure 1C:

<http://ualcan.path.uab.edu/cgi-bin/CPTAC-Result.pl?genenam=STEAP3&ctype=HNSC>

<http://ualcan.path.uab.edu/cgi-bin/CPTAC-Result.pl?genenam=STEAP3&ctype=GBM>

<http://ualcan.path.uab.edu/cgi-bin/CPTAC-Result.pl?genenam=STEAP3&ctype=LUAD>

<http://ualcan.path.uab.edu/cgi-bin/CPTAC-Result.pl?genenam=STEAP3&ctype=UCEC>

<http://ualcan.path.uab.edu/cgi-bin/CPTAC-Result.pl?genenam=STEAP3&ctype=Liver>

Figure 1D:

Brain normal

<https://www.proteinatlas.org/ENSG00000115107-STEAP3/tissue/cerebellum#img>

GBM

<https://www.proteinatlas.org/ENSG00000115107-STEAP3/pathology/glioma#img>

Lung normal

<https://www.proteinatlas.org/ENSG00000115107-STEAP3/tissue/lung#img>

LUAD

<https://www.proteinatlas.org/ENSG00000115107-STEAP3/pathology/lung+cancer#img>

Uterine normal

<https://www.proteinatlas.org/ENSG00000115107-STEAP3/tissue/cervix#img>

UCEC

<https://www.proteinatlas.org/ENSG00000115107-STEAP3/pathology/endometrial+cancer#img>

Liver normal

<https://www.proteinatlas.org/ENSG00000115107-STEAP3/tissue/liver#img>

LIHC

<https://www.proteinatlas.org/ENSG00000115107-STEAP3/pathology/liver+cancer#img>
