# Supplementary material for: High expression of six-transmembrane epithelial antigen of prostate 3 promotes the migration and invasion and predicts unfavorable prognosis in glioma
Source: PeerJ. 2023 Mar 28;11:e15136. doi: 10.7717/peerj.15136 (PMC10065001; doi:10.7717/peerj.15136)

Figure 2A:

Overall survival


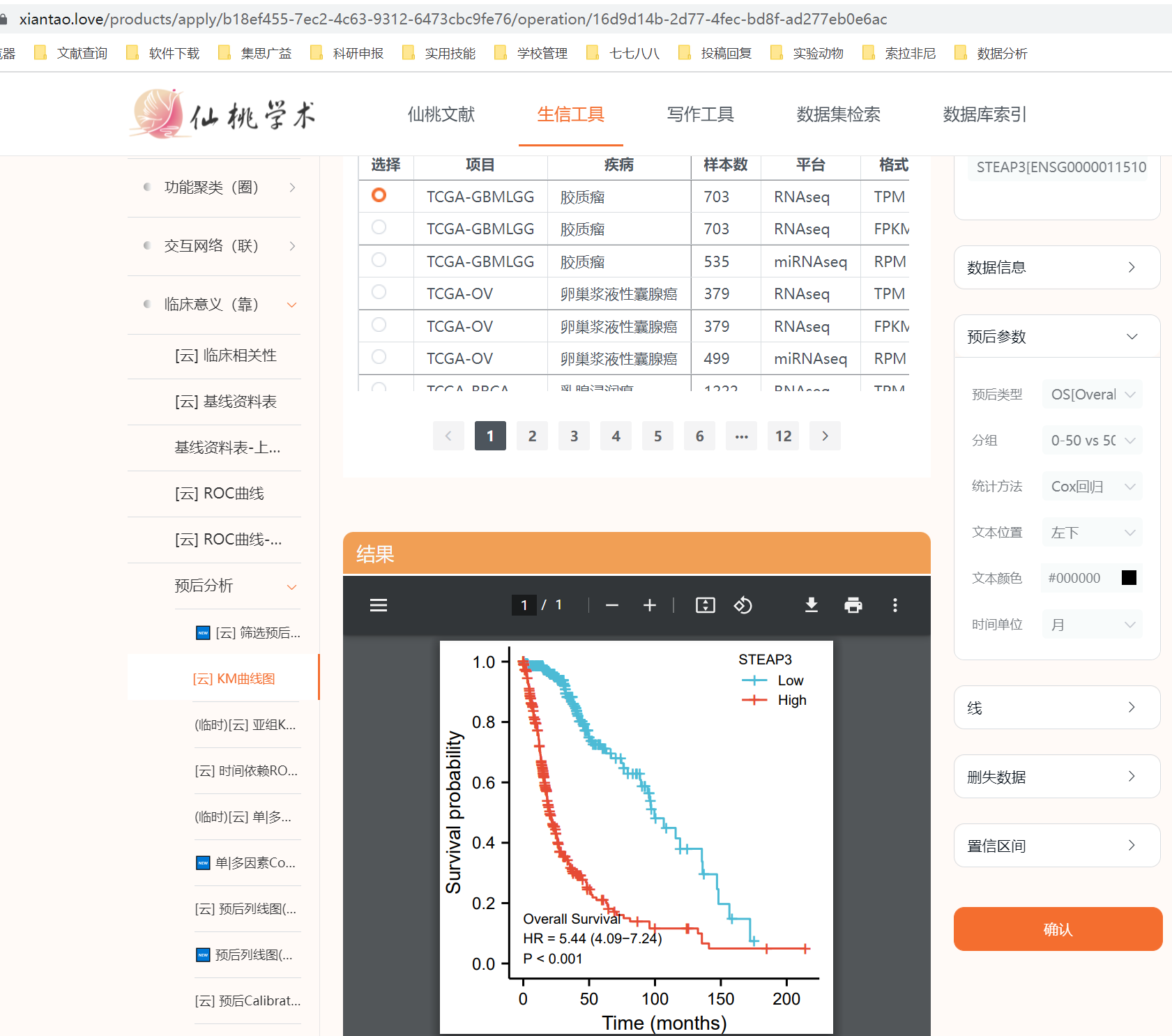


Disease specific survival


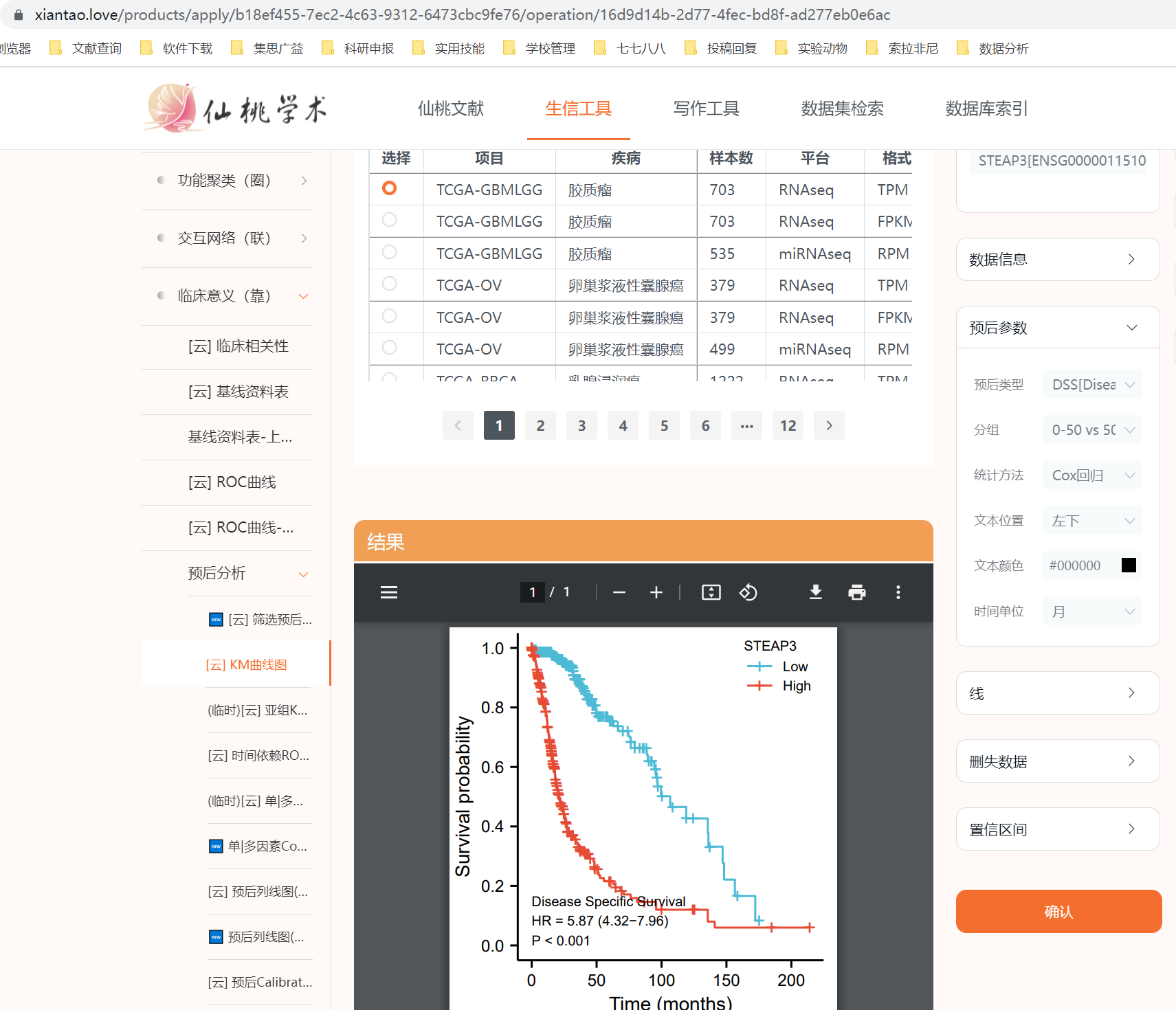


Progression free survival


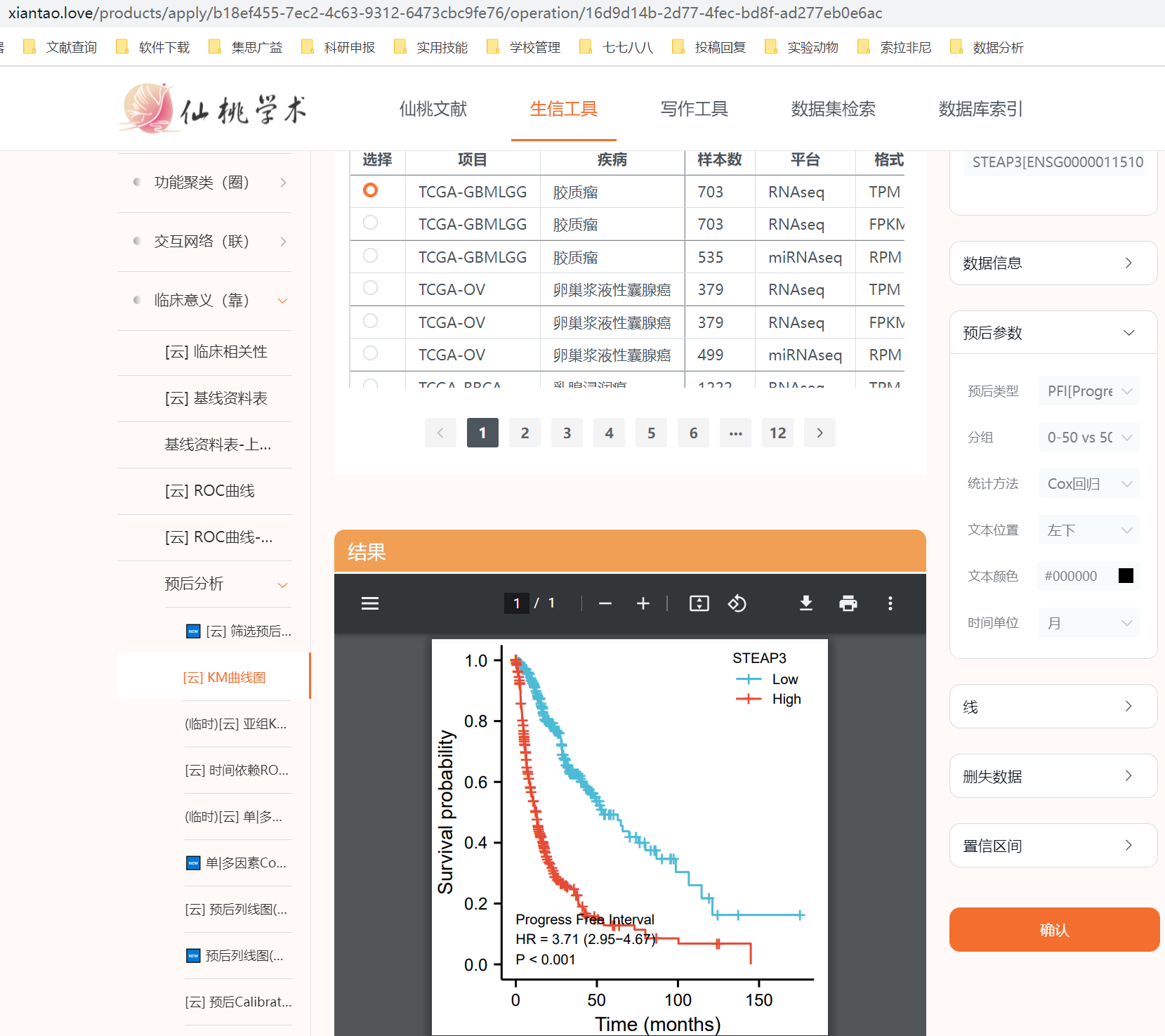


Figure 2B:

Overall survival


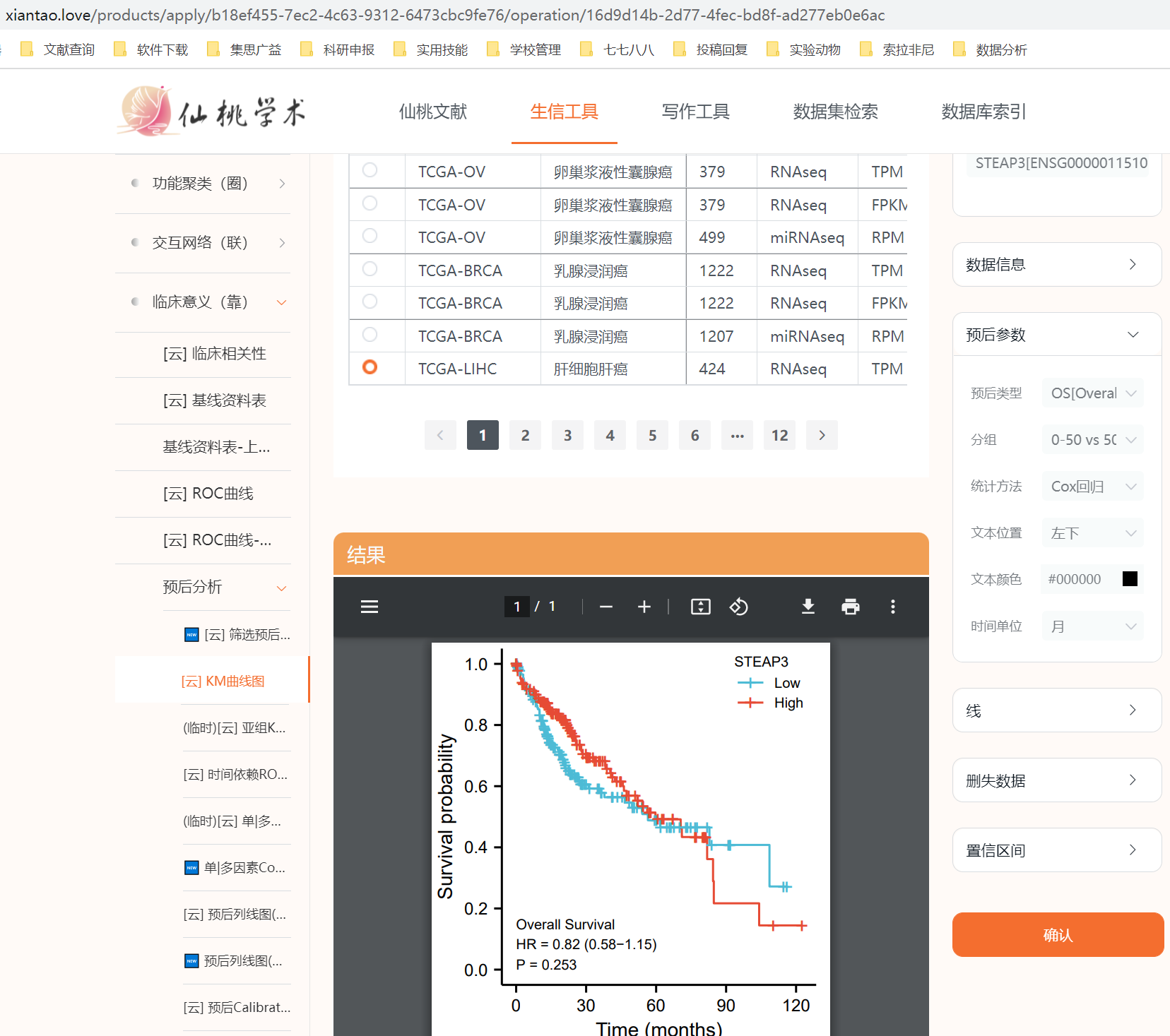


Disease specific survival


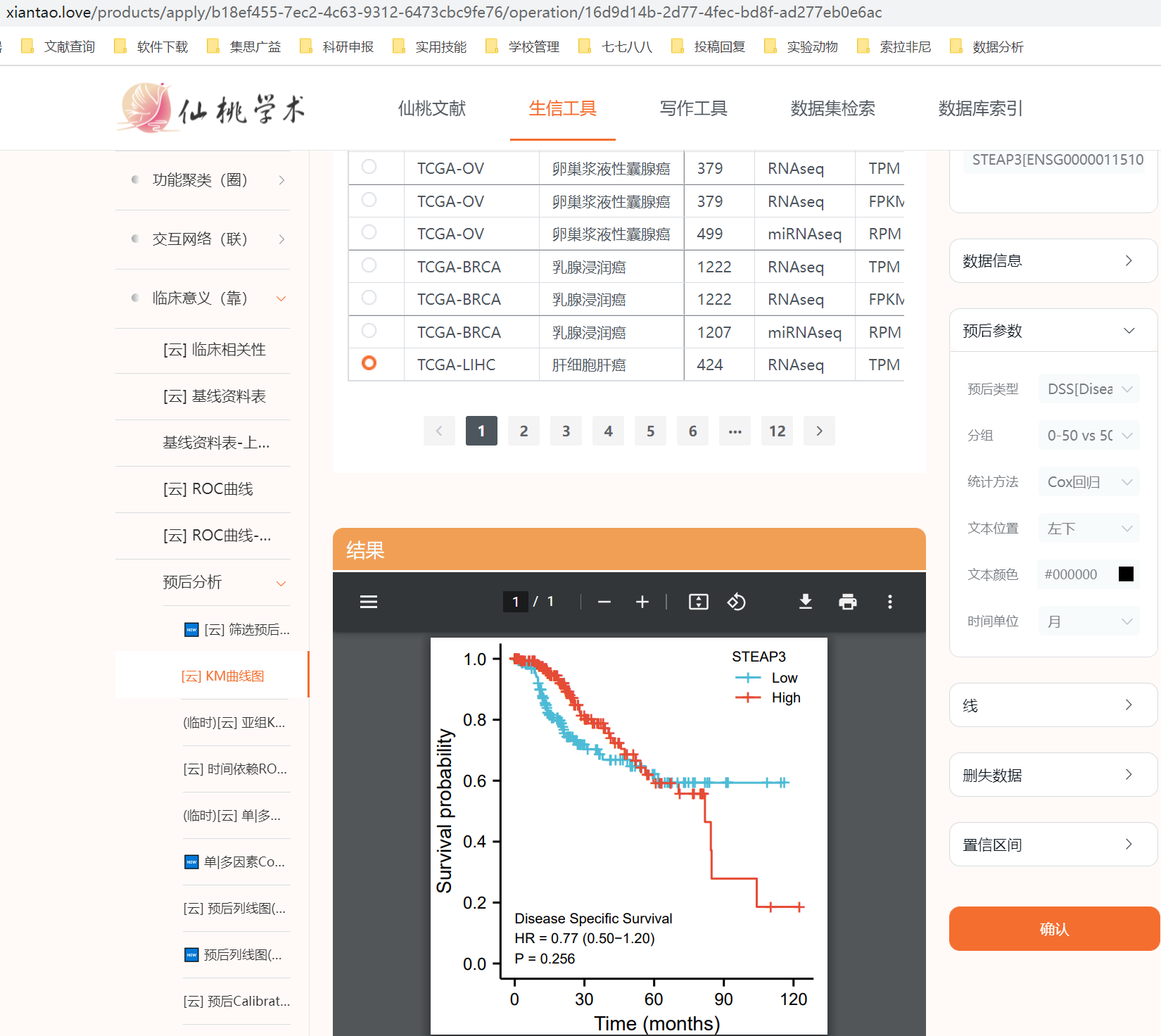


Progression free survival


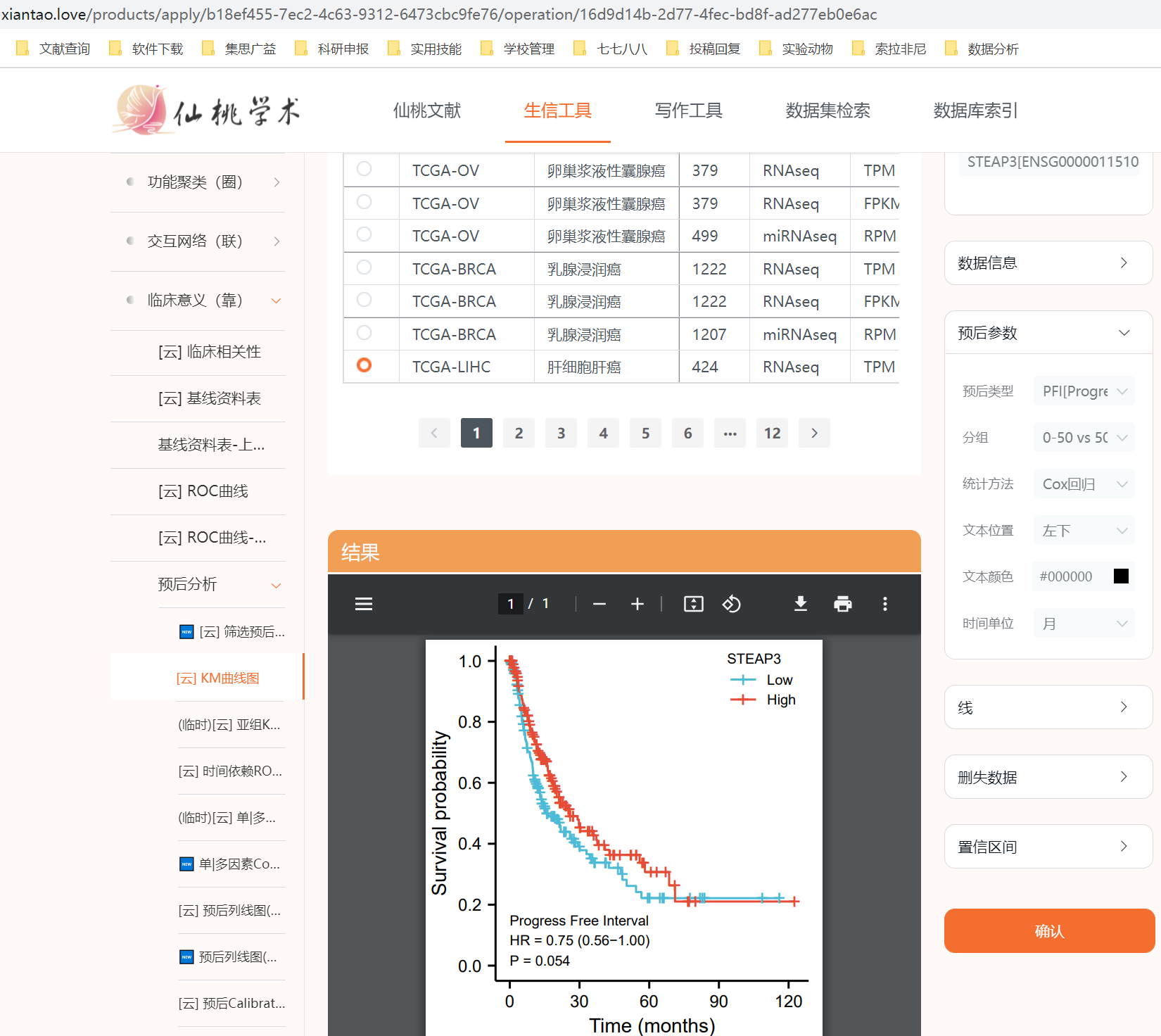


Figure 2C:

Overall survival


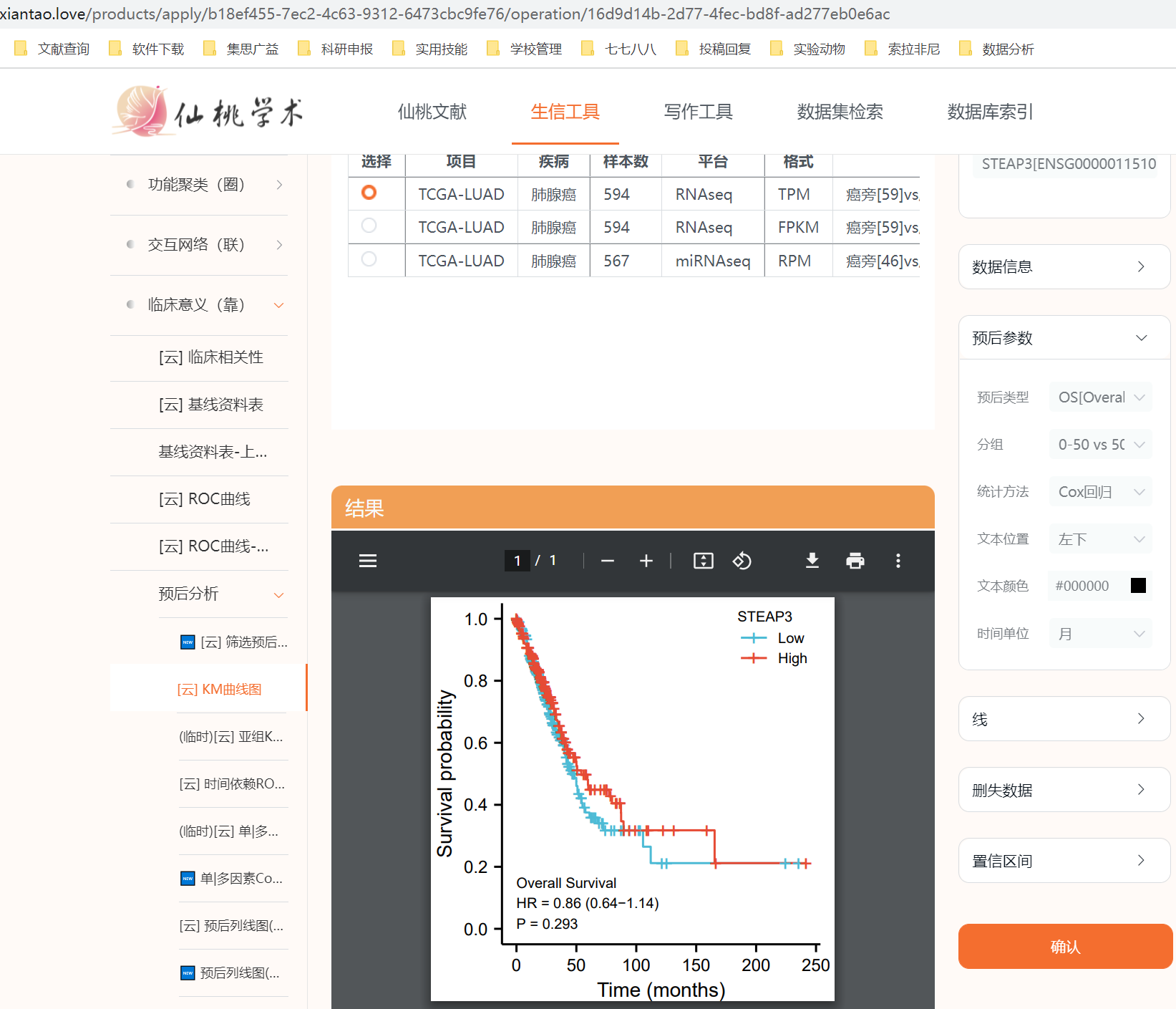


Disease specific survival


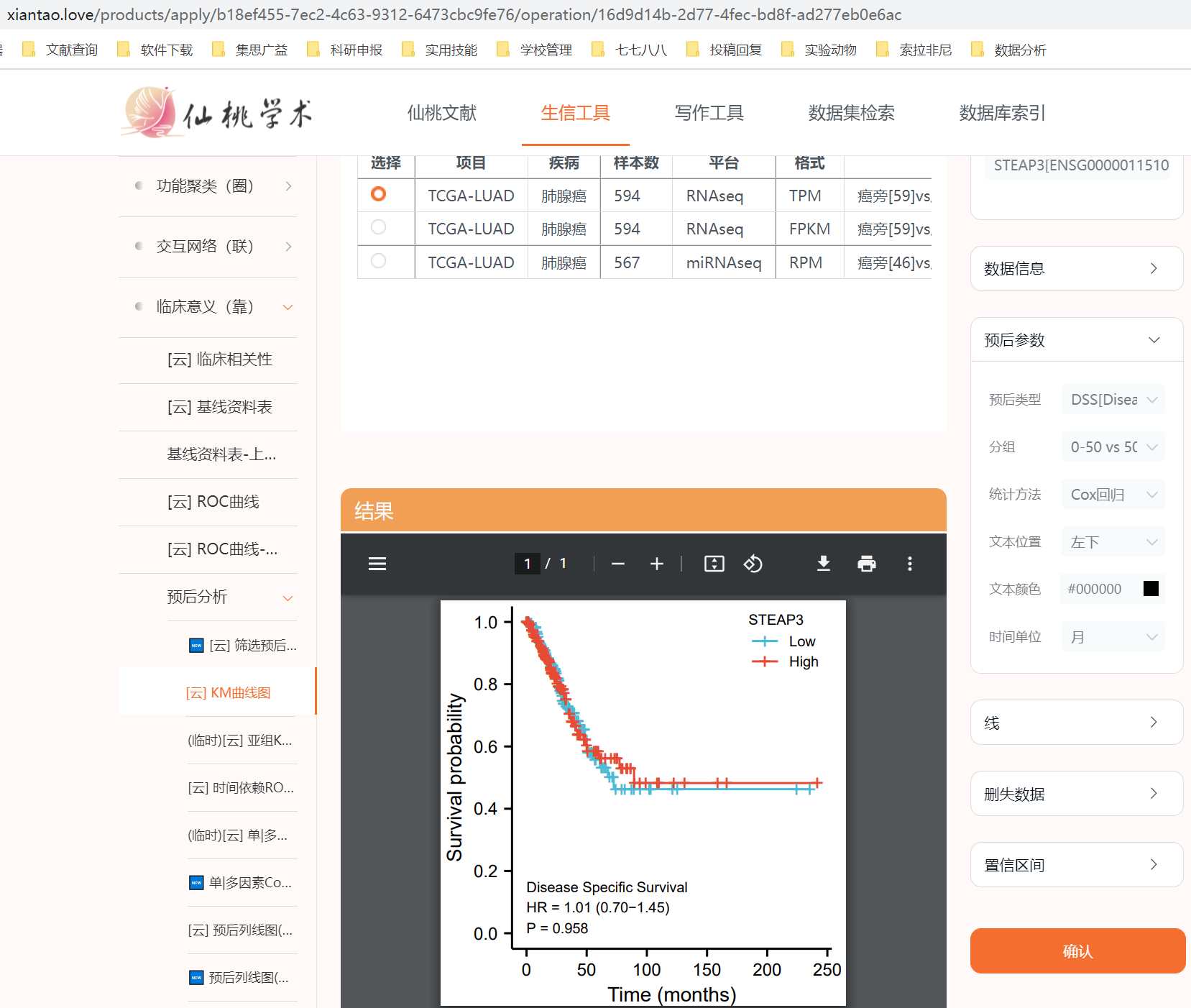


Progression free survival


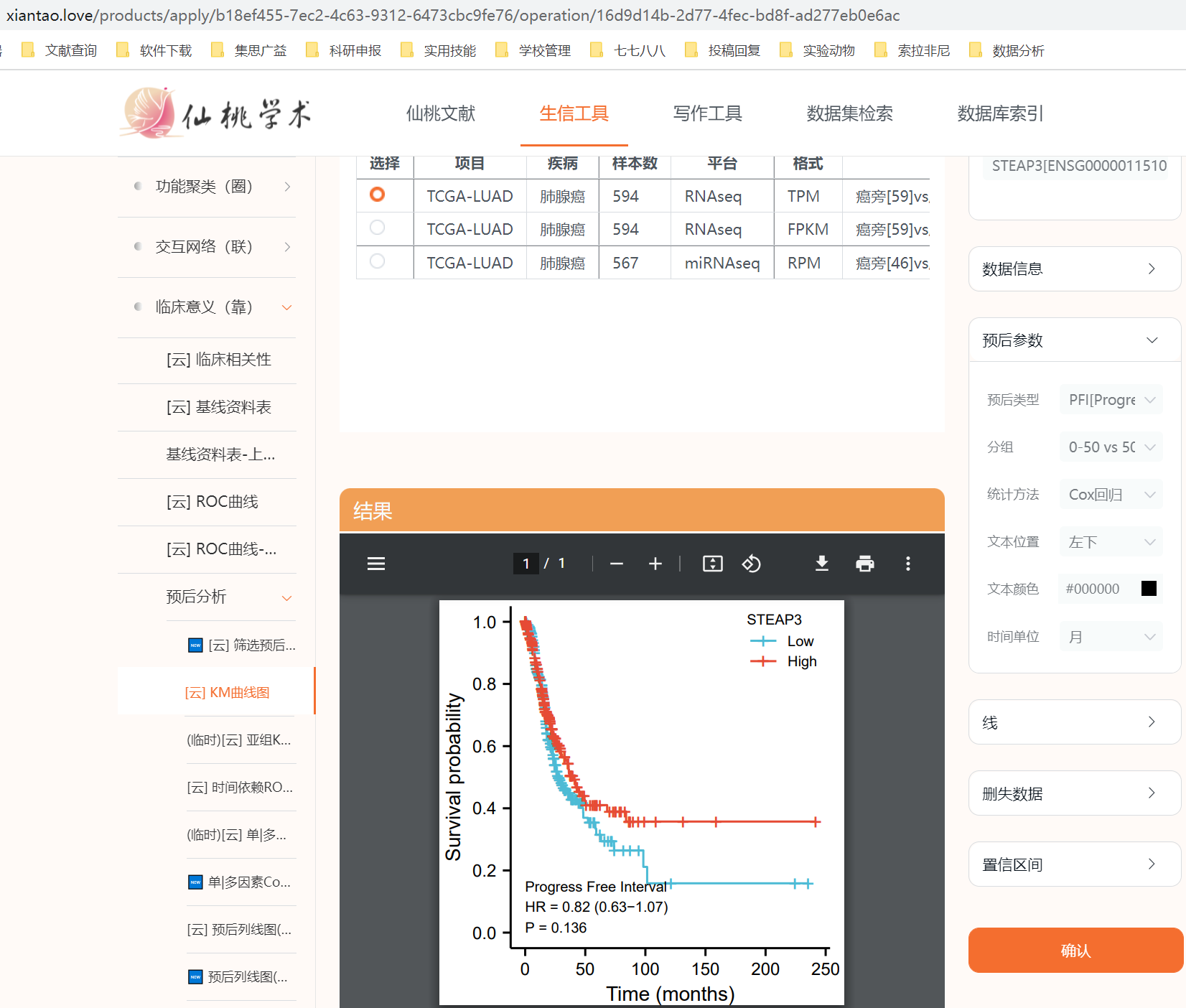


Figure 2D:

Overall survival


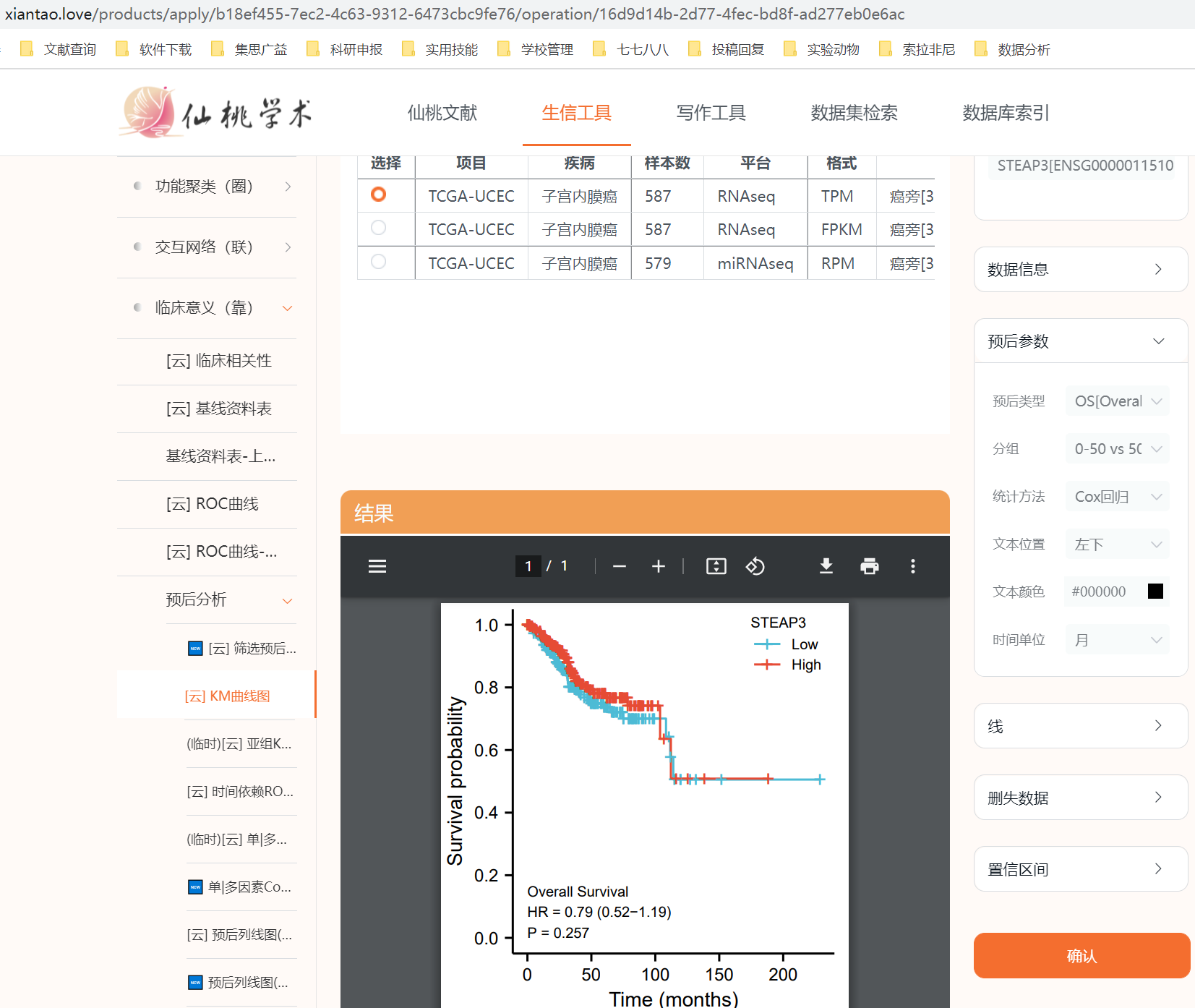


Disease specific survival


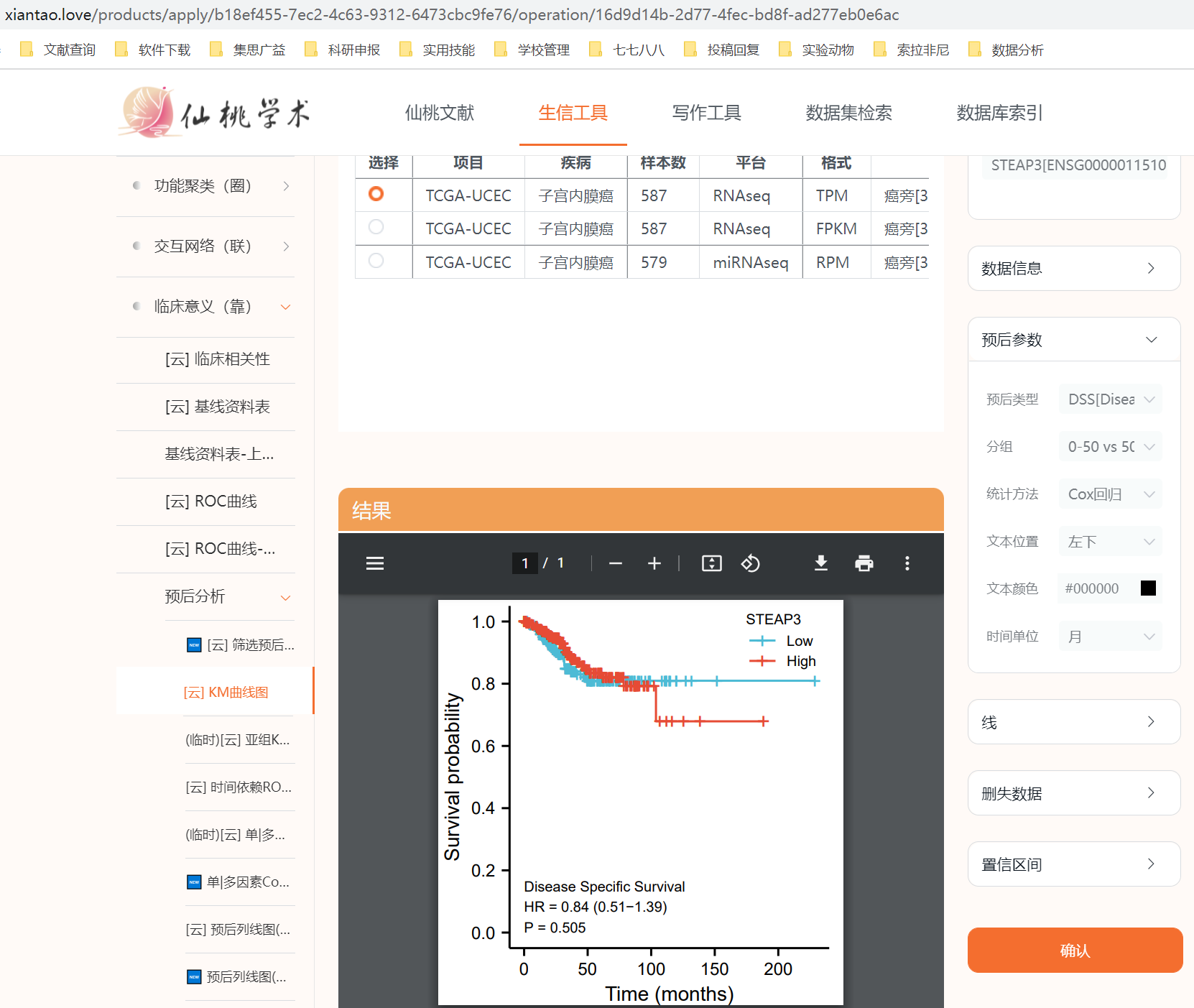


Progression free survival


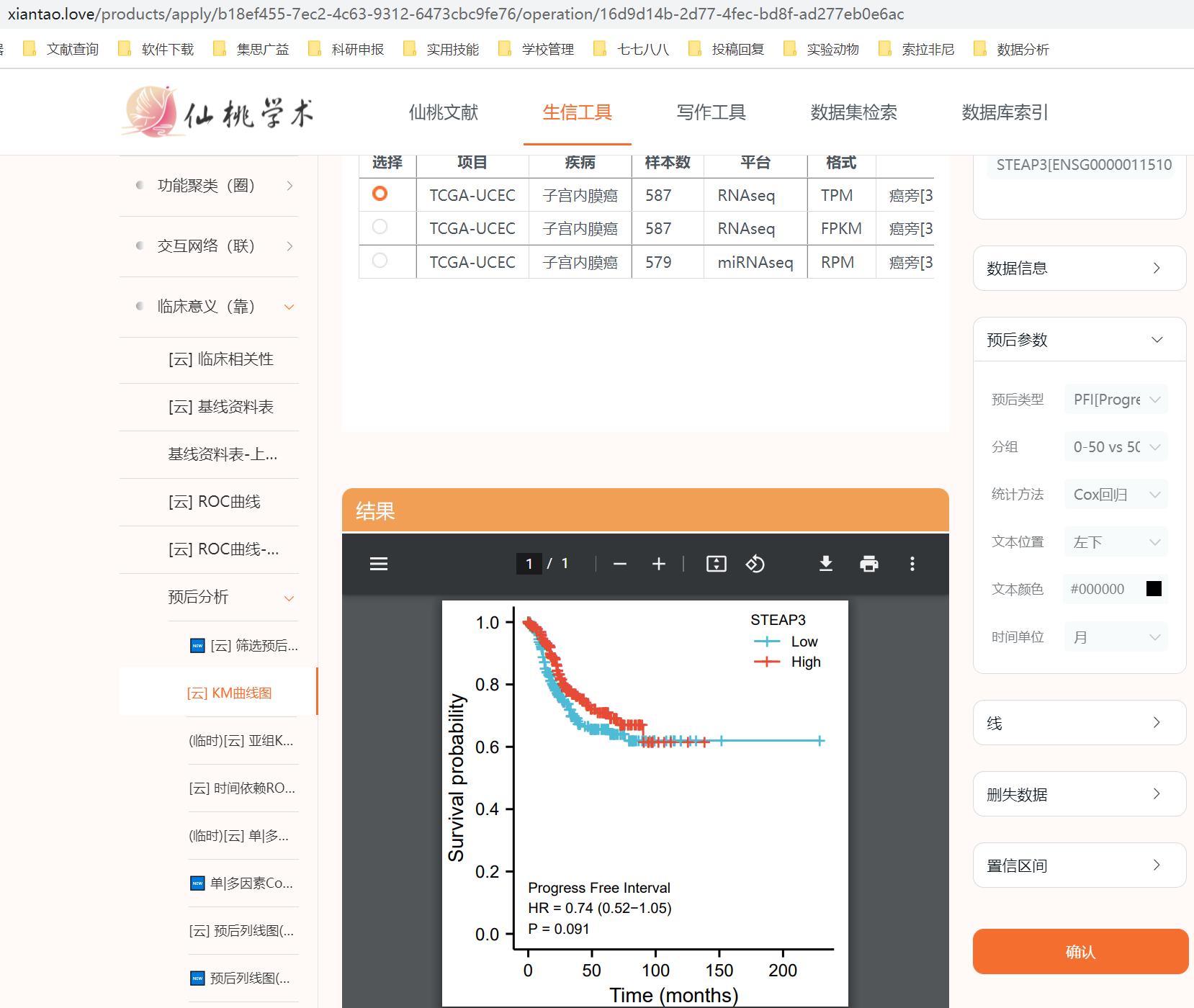

Supplement: Supplemental Information 7 — Prognostic value of STEAP3 in glioma, LIHC, LUAD, and UCEC. [file peerj-11-15136-s007.zip › raw data for Figure 2/Raw data for Figure 2A-D.docx]
