# Supplementary material for: High expression of six-transmembrane epithelial antigen of prostate 3 promotes the migration and invasion and predicts unfavorable prognosis in glioma
Source: PeerJ. 2023 Mar 28;11:e15136. doi: 10.7717/peerj.15136 (PMC10065001; doi:10.7717/peerj.15136)

Figure 3A-H:

<http://www.cgga.org.cn/analyse/RNA-data-expression-distribution-result.jsp>


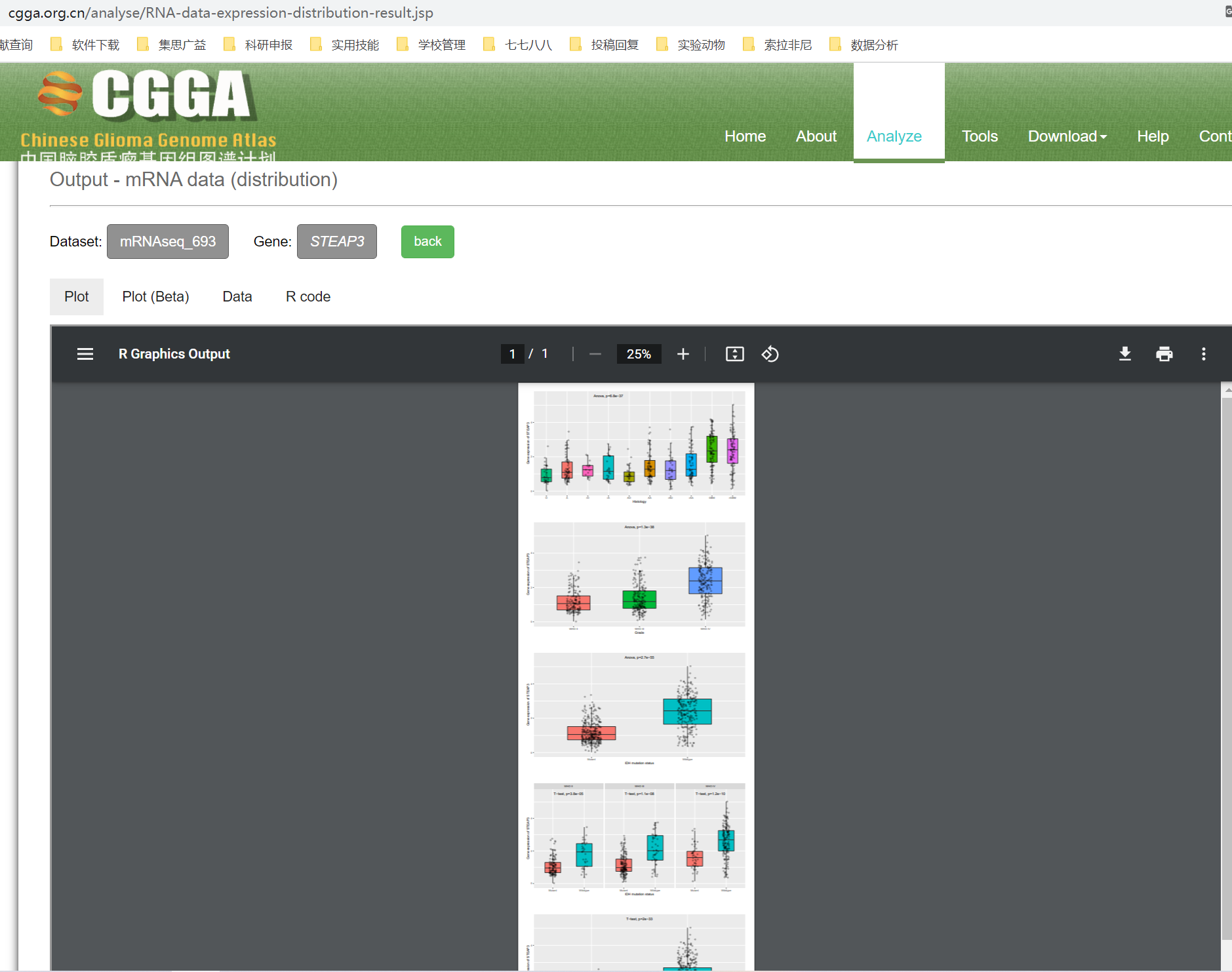


Figure 3I-J:

<http://www.cgga.org.cn/analyse/RNA-data-survival-result.jsp>


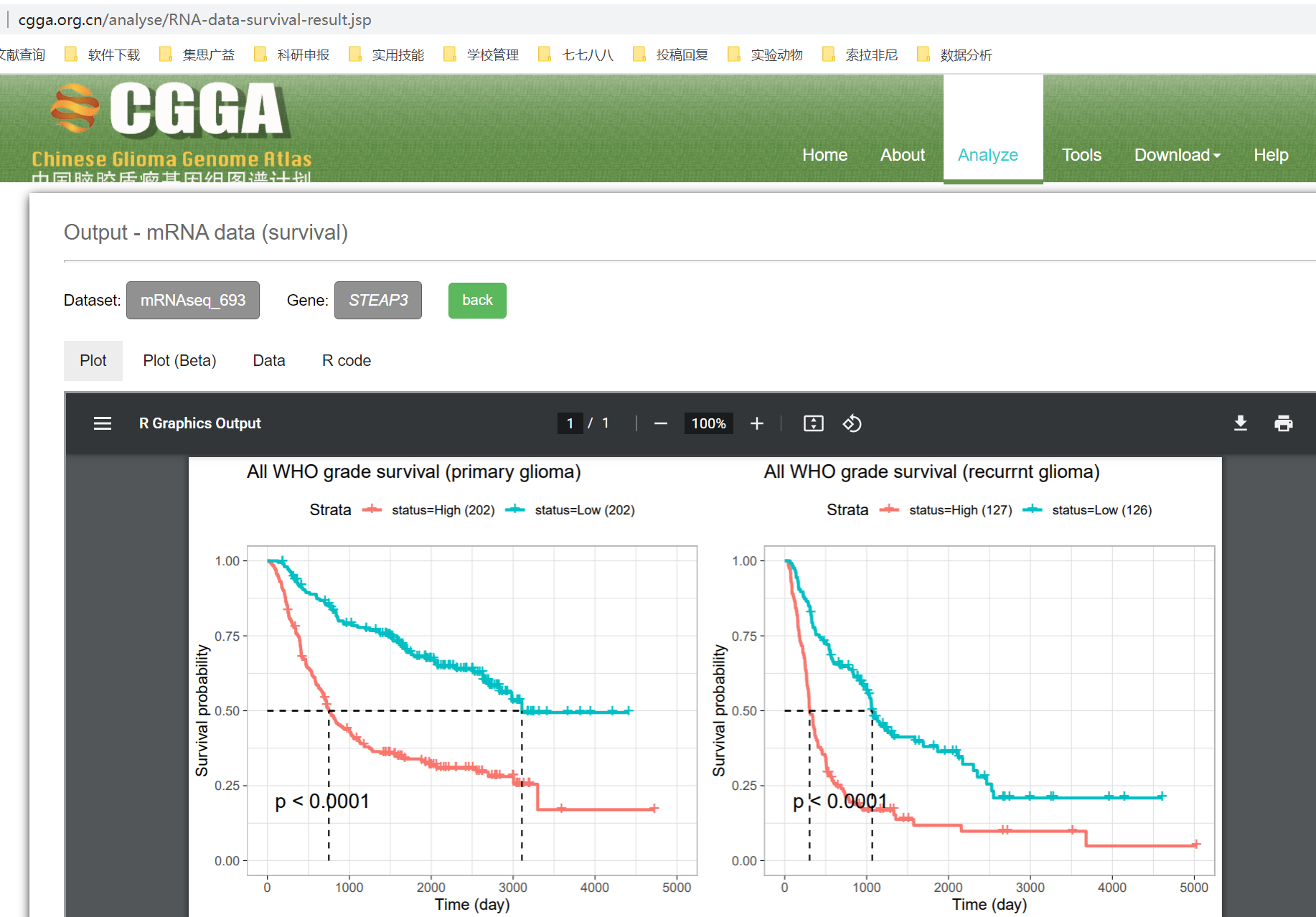

Supplement: Supplemental Information 8 — Correlation between STEAP3 expression and clinicopathological characteristics in glioma. [file peerj-11-15136-s008.zip › raw data for Figure 3/Raw data for Figure 3A-J.docx]
