# Supplementary material for: High expression of six-transmembrane epithelial antigen of prostate 3 promotes the migration and invasion and predicts unfavorable prognosis in glioma
Source: PeerJ. 2023 Mar 28;11:e15136. doi: 10.7717/peerj.15136 (PMC10065001; doi:10.7717/peerj.15136)

Figure 4A-B:

<http://www.cgga.org.cn/analyse/Methyl-data-distribution-result.jsp>


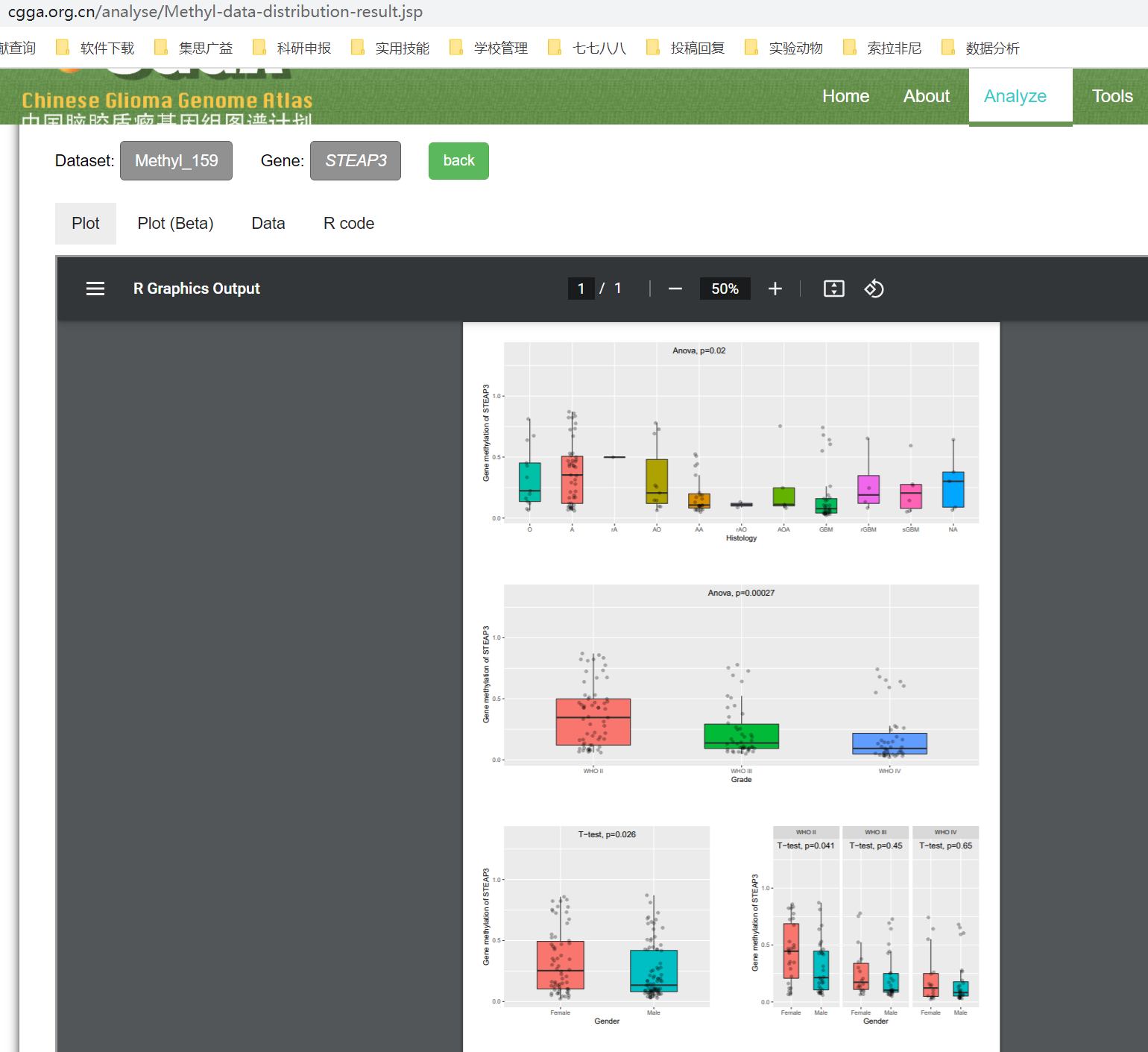


Figure 4C:

<http://www.cgga.org.cn/analyse/Methyl-data-survival-result.jsp>
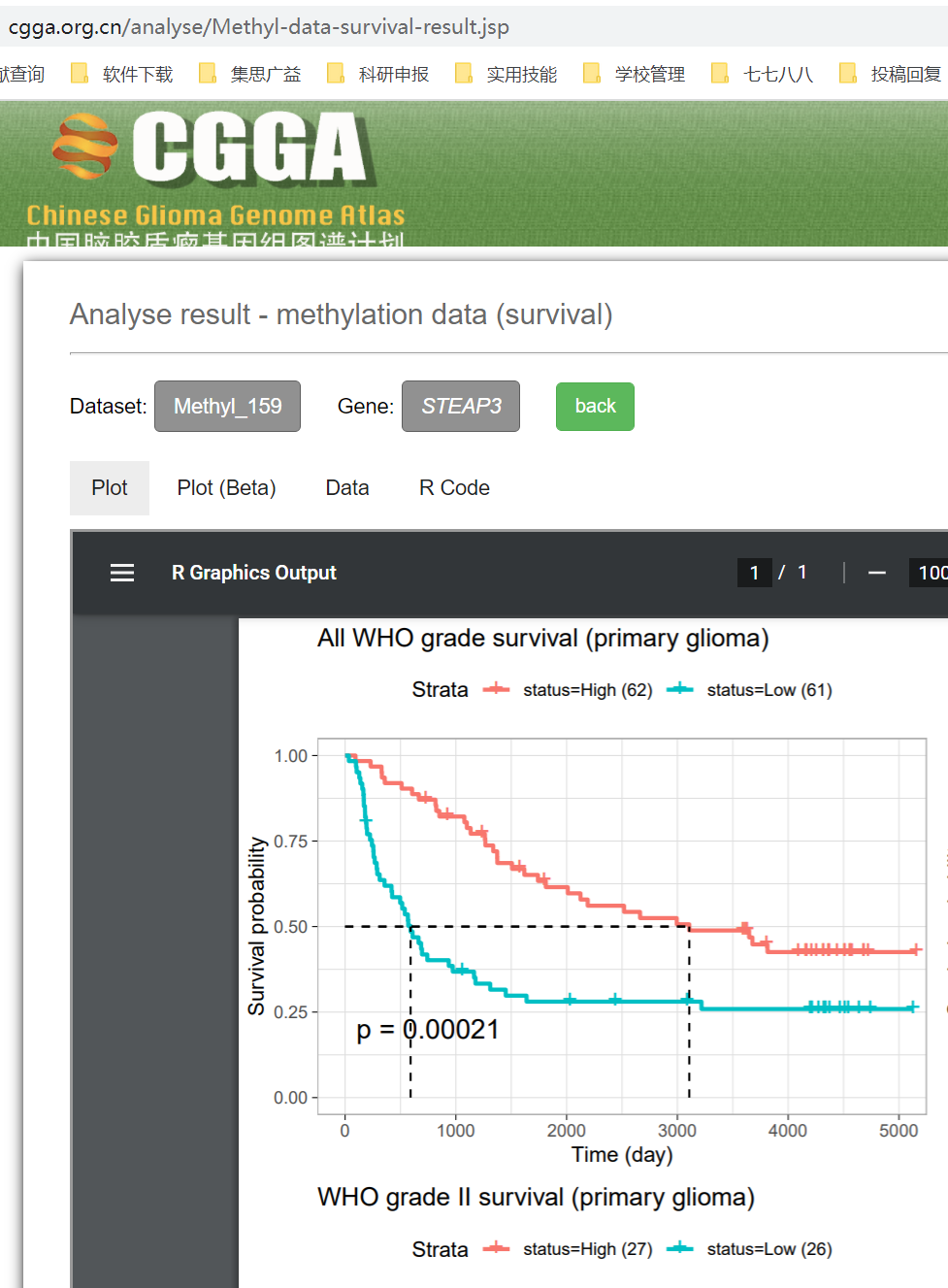


Figure 4D:


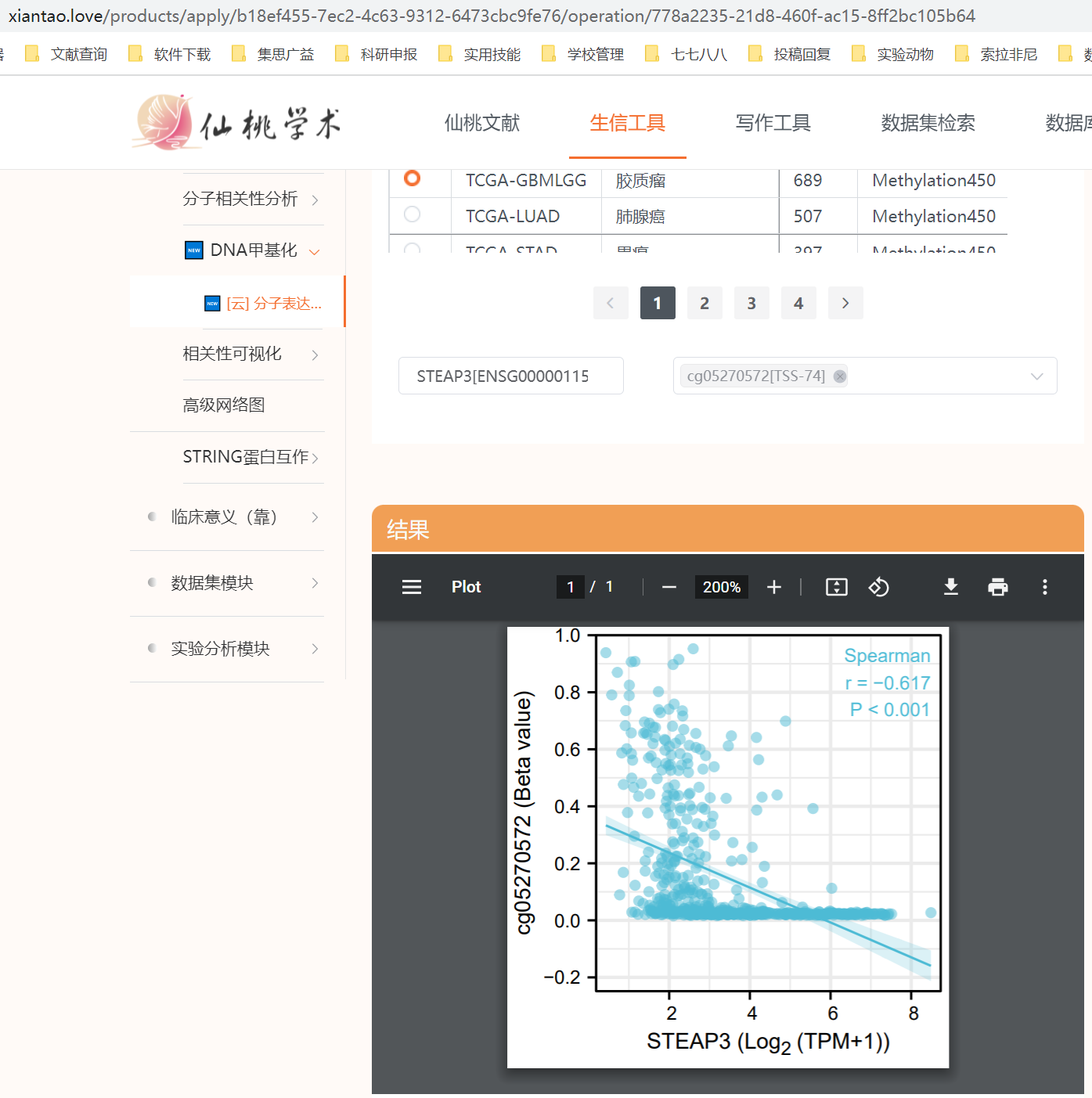


Figure 4E:


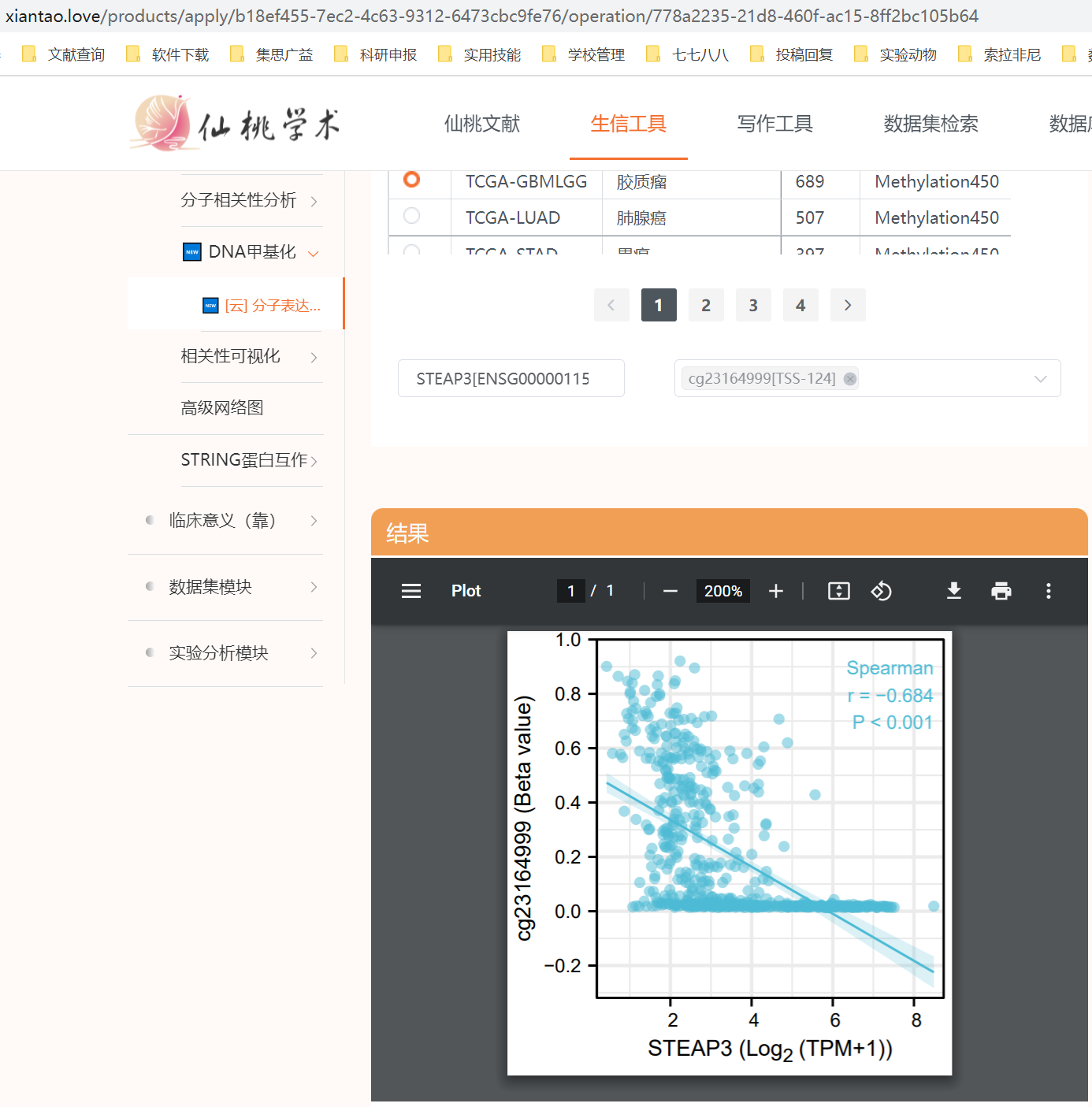


Figure 4F:


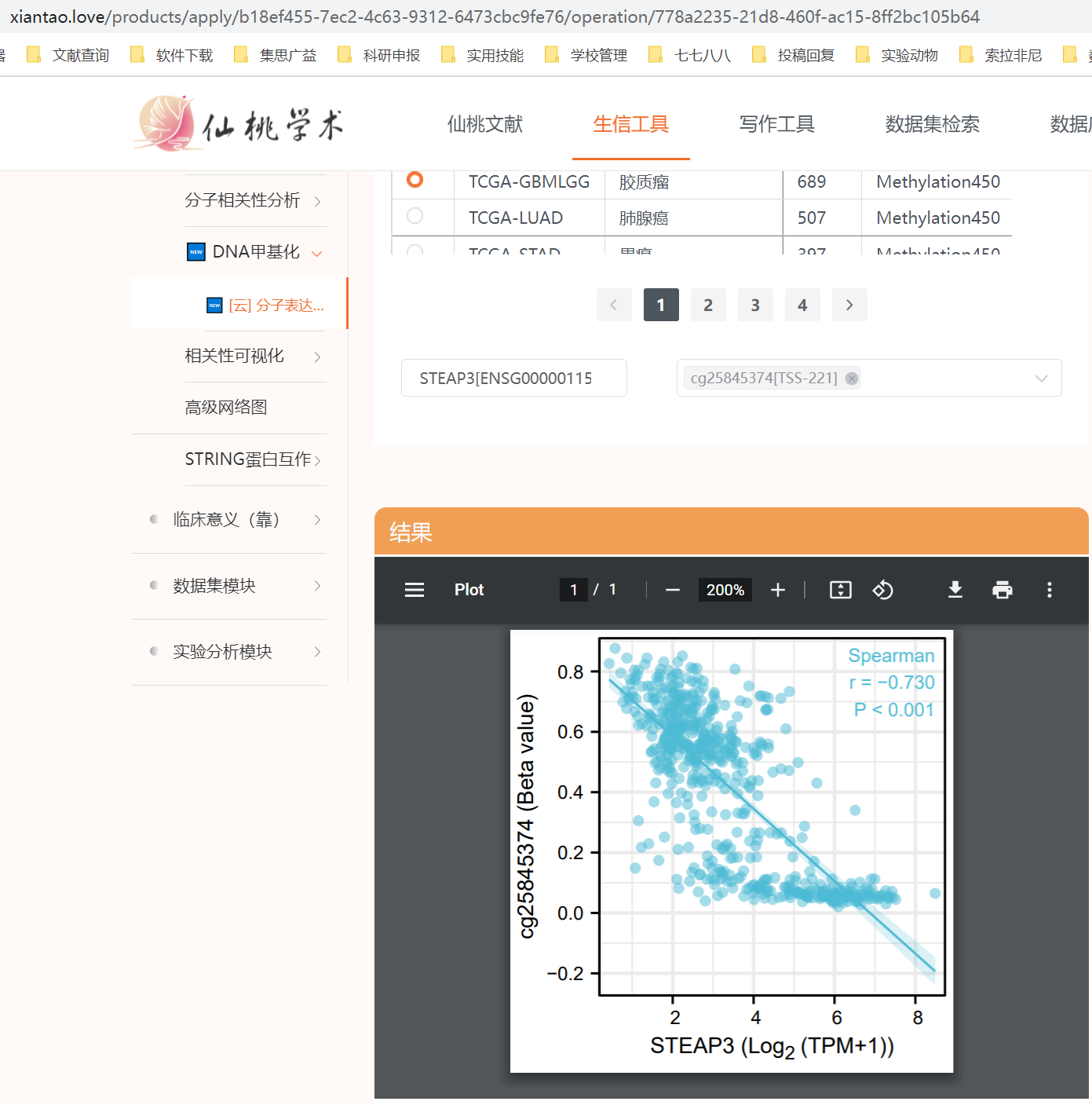


Figure 4G:


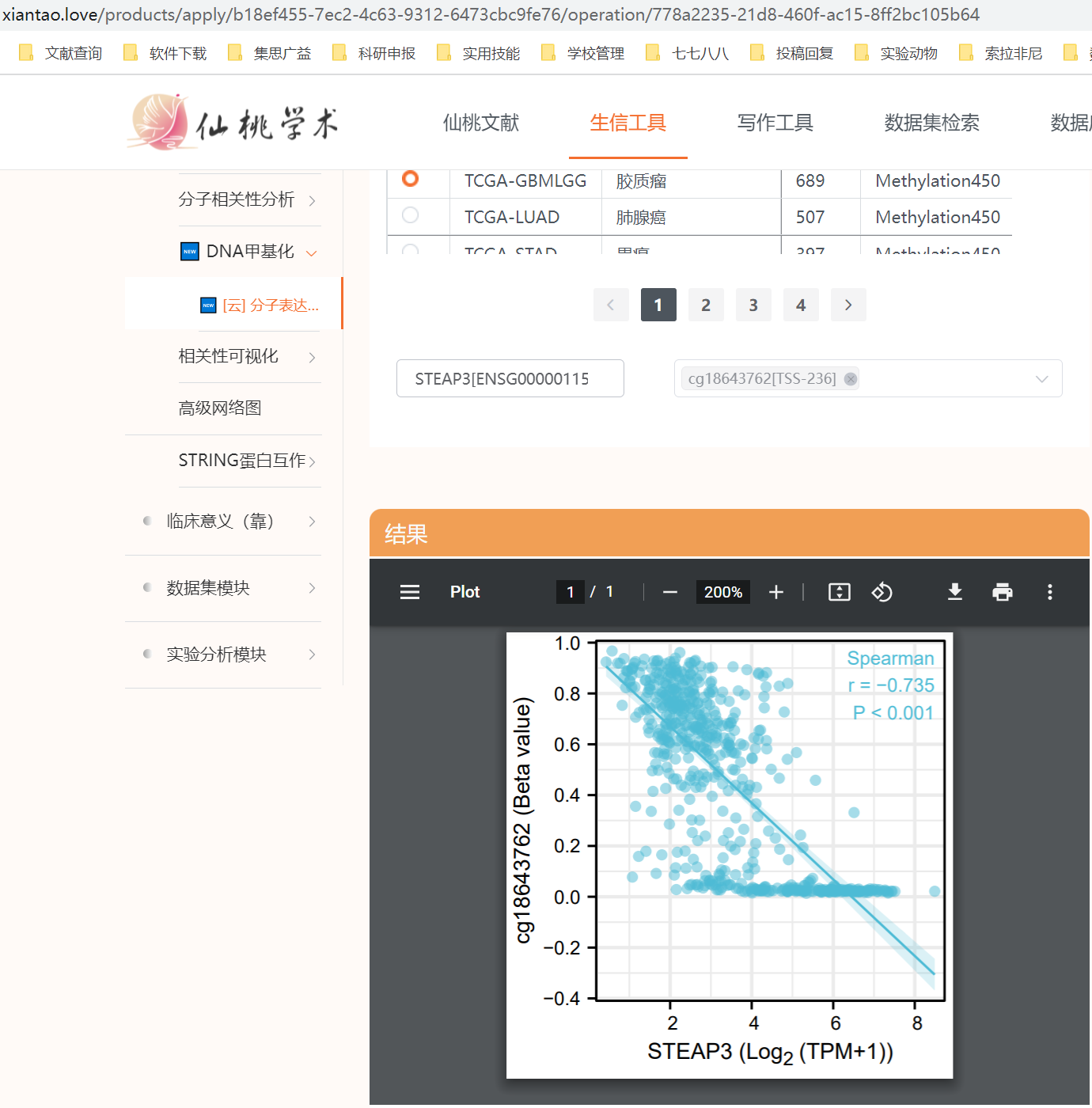


Figure 4H:


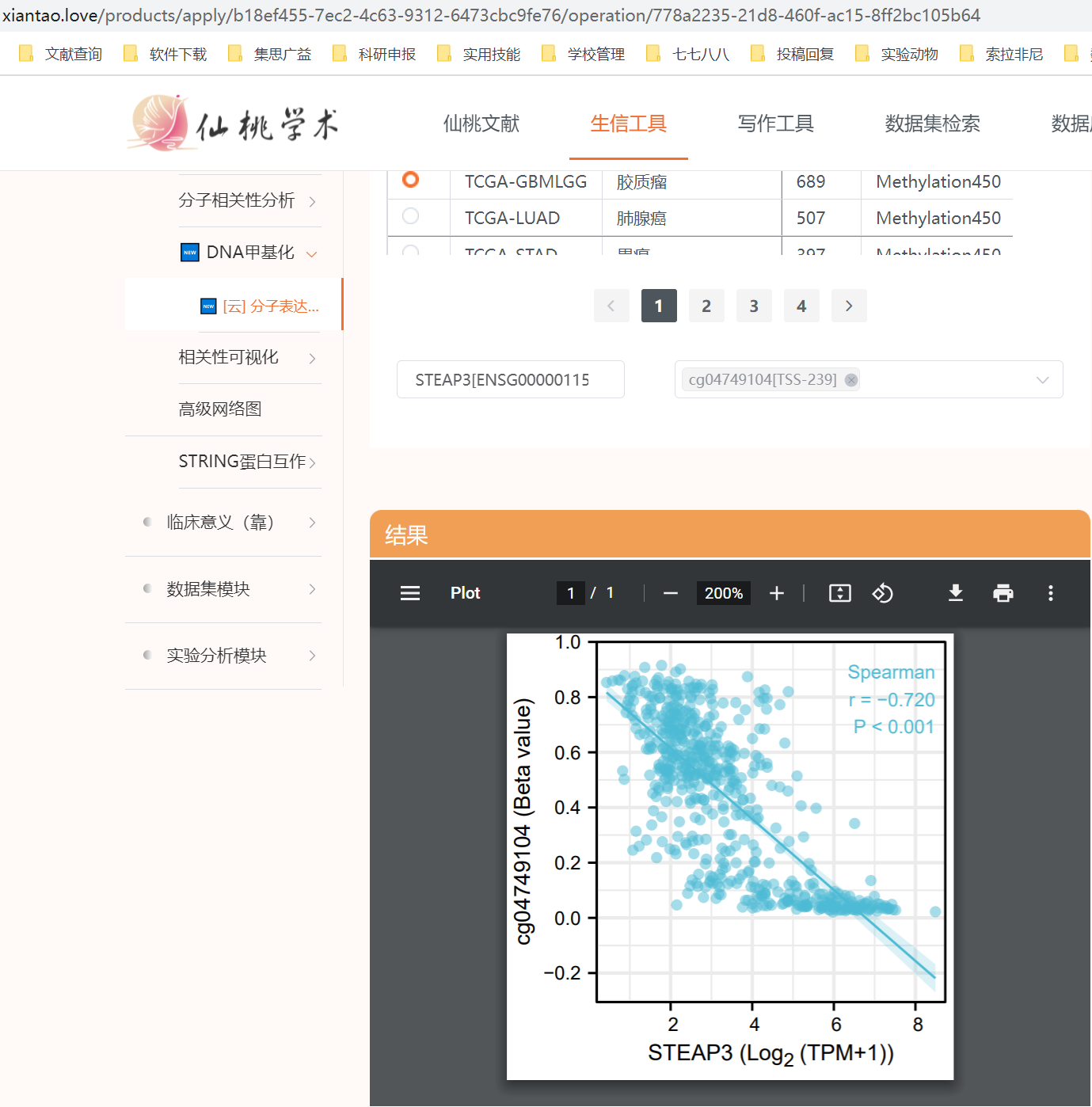


Figure 4I:


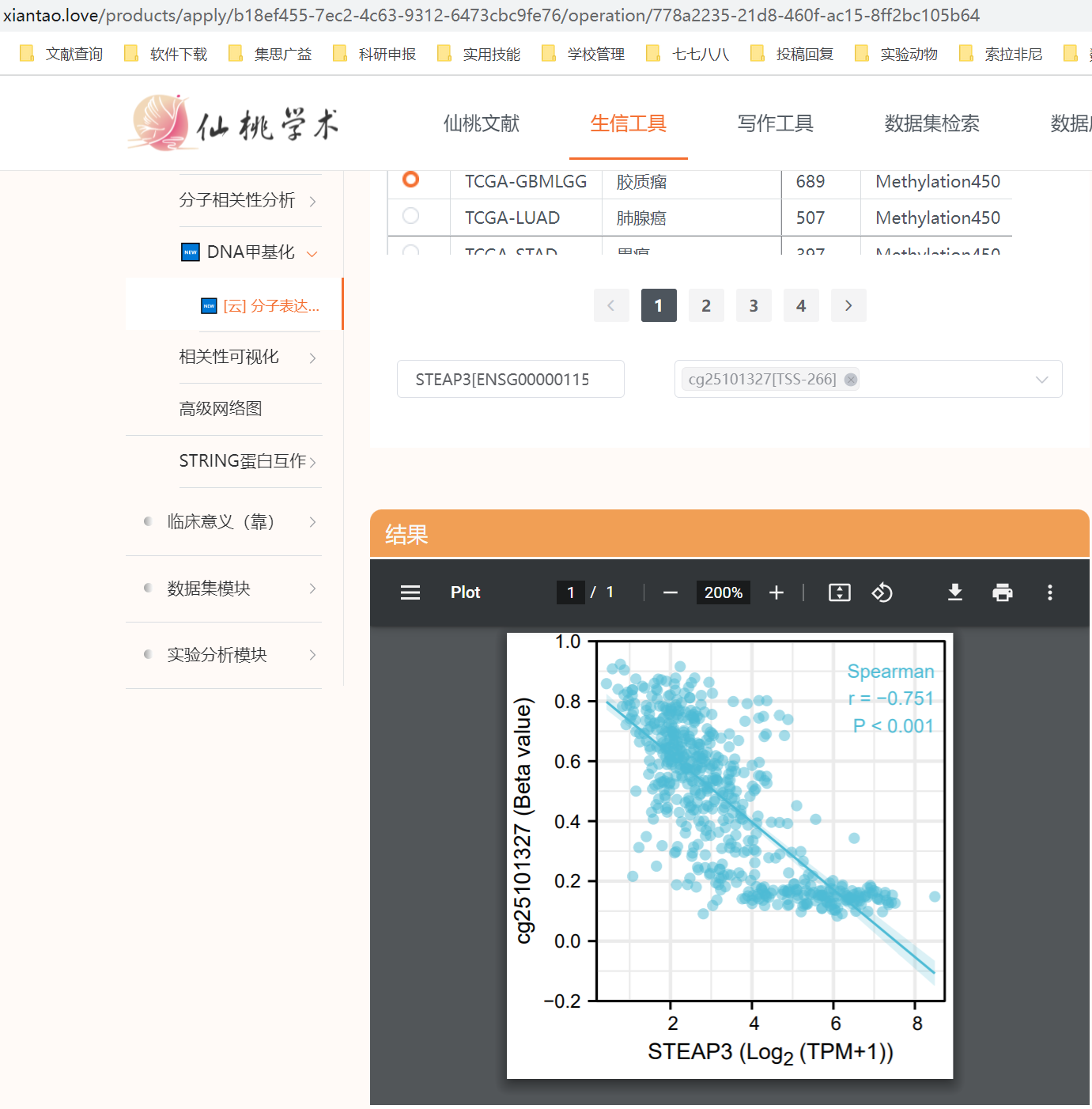

Supplement: Supplemental Information 9 — Correlation between STEAP3 methylation and clinicopathological characteristics in glioma. [file peerj-11-15136-s009.zip › raw data for Figure 4/Raw data for Figure 4A-I.docx]
