# Supplementary material for: High expression of six-transmembrane epithelial antigen of prostate 3 promotes the migration and invasion and predicts unfavorable prognosis in glioma
Source: PeerJ. 2023 Mar 28;11:e15136. doi: 10.7717/peerj.15136 (PMC10065001; doi:10.7717/peerj.15136)

Figure 5A:

<http://biocc.hrbmu.edu.cn/CancerSEA/calForSingle?singleGene=STEAP3&submit=Search#datasetsDetail>

Figure 5B:


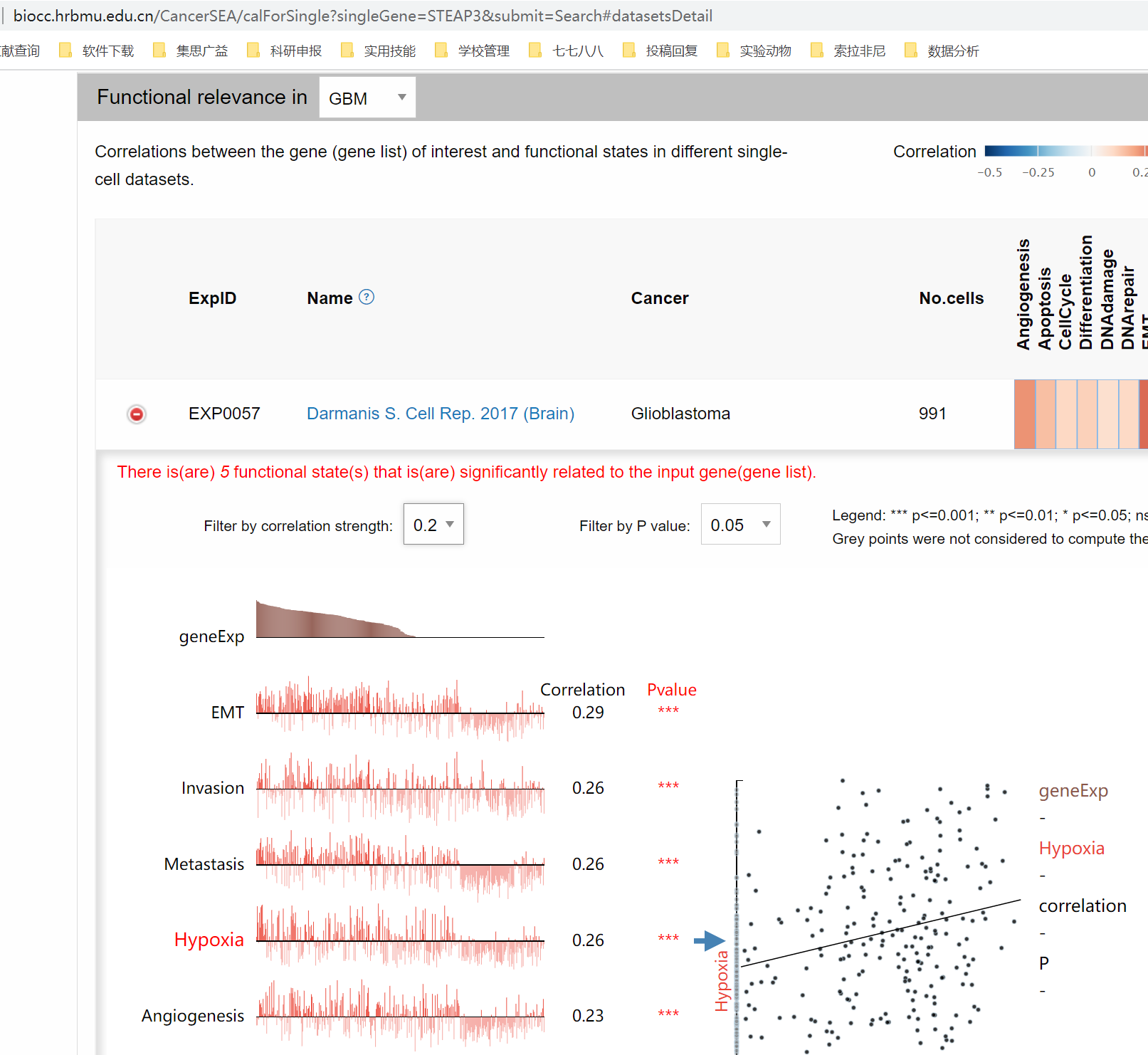

Supplement: Supplemental Information 10 — Functional relevance of STEAP3 in glioma. [file peerj-11-15136-s010.zip › raw data for Figure 5/Raw data for Figure 5A-B.docx]
