# Supplementary material for: High expression of six-transmembrane epithelial antigen of prostate 3 promotes the migration and invasion and predicts unfavorable prognosis in glioma
Source: PeerJ. 2023 Mar 28;11:e15136. doi: 10.7717/peerj.15136 (PMC10065001; doi:10.7717/peerj.15136)

Figure 6A:

<http://linkedomics.org/lo_batchfile/lo_TCGAresult/lo_result_120374.png>


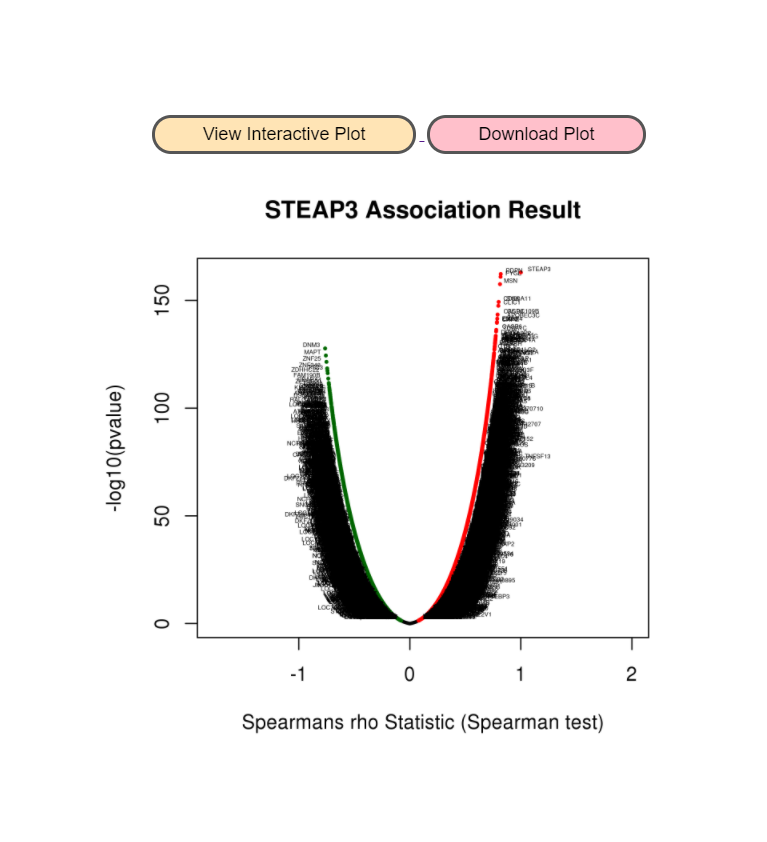


Figure 6B:

<http://linkedomics.org/lo_batchfile/lo_TCGAresult/lo_result_120374_pos_heatplot.png>


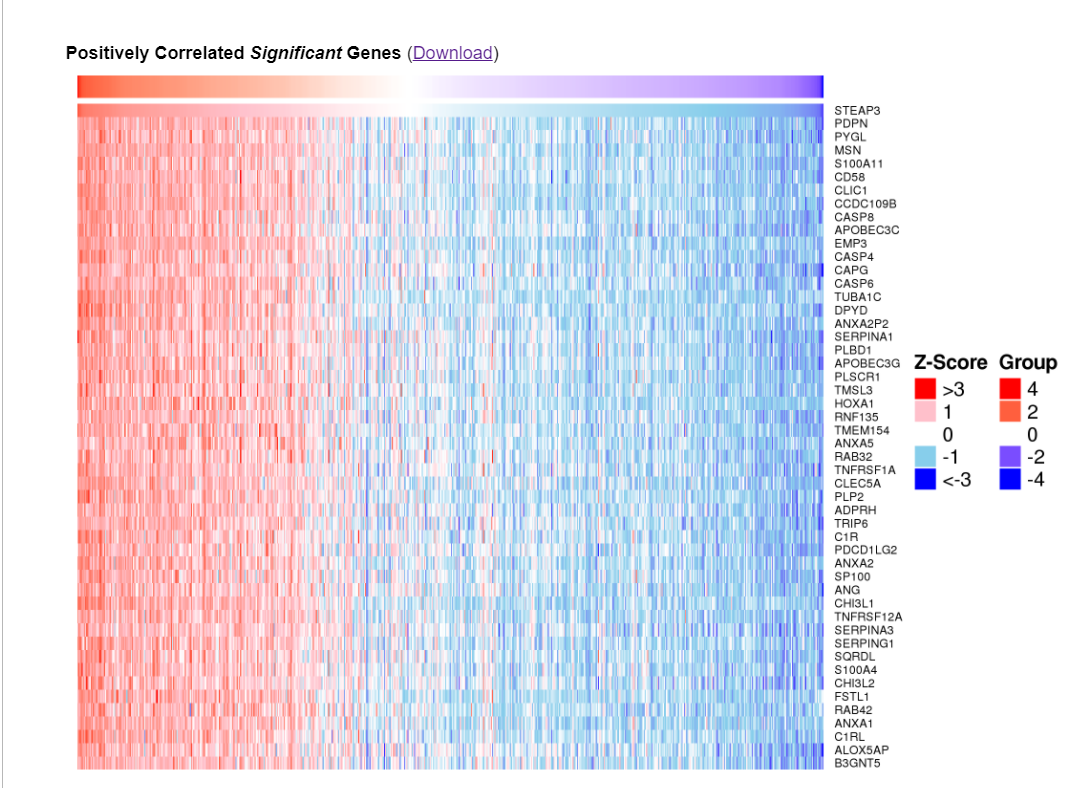


Figure 6C:

<http://linkedomics.org/lo_batchfile/lo_TCGAresult/lo_result_120374_neg_heatplot.png>


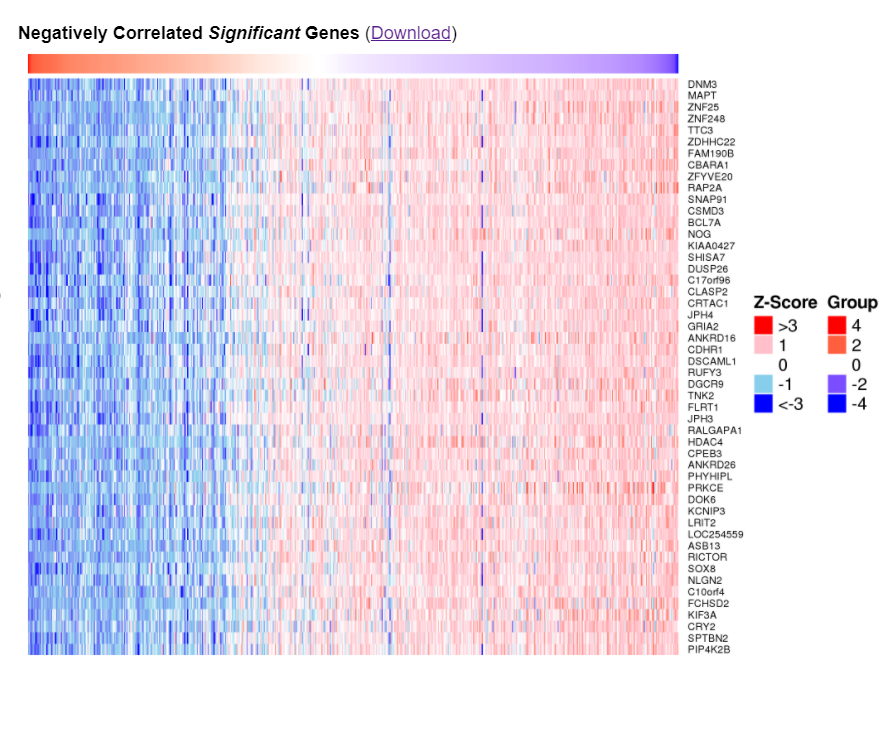


Figure 6D:


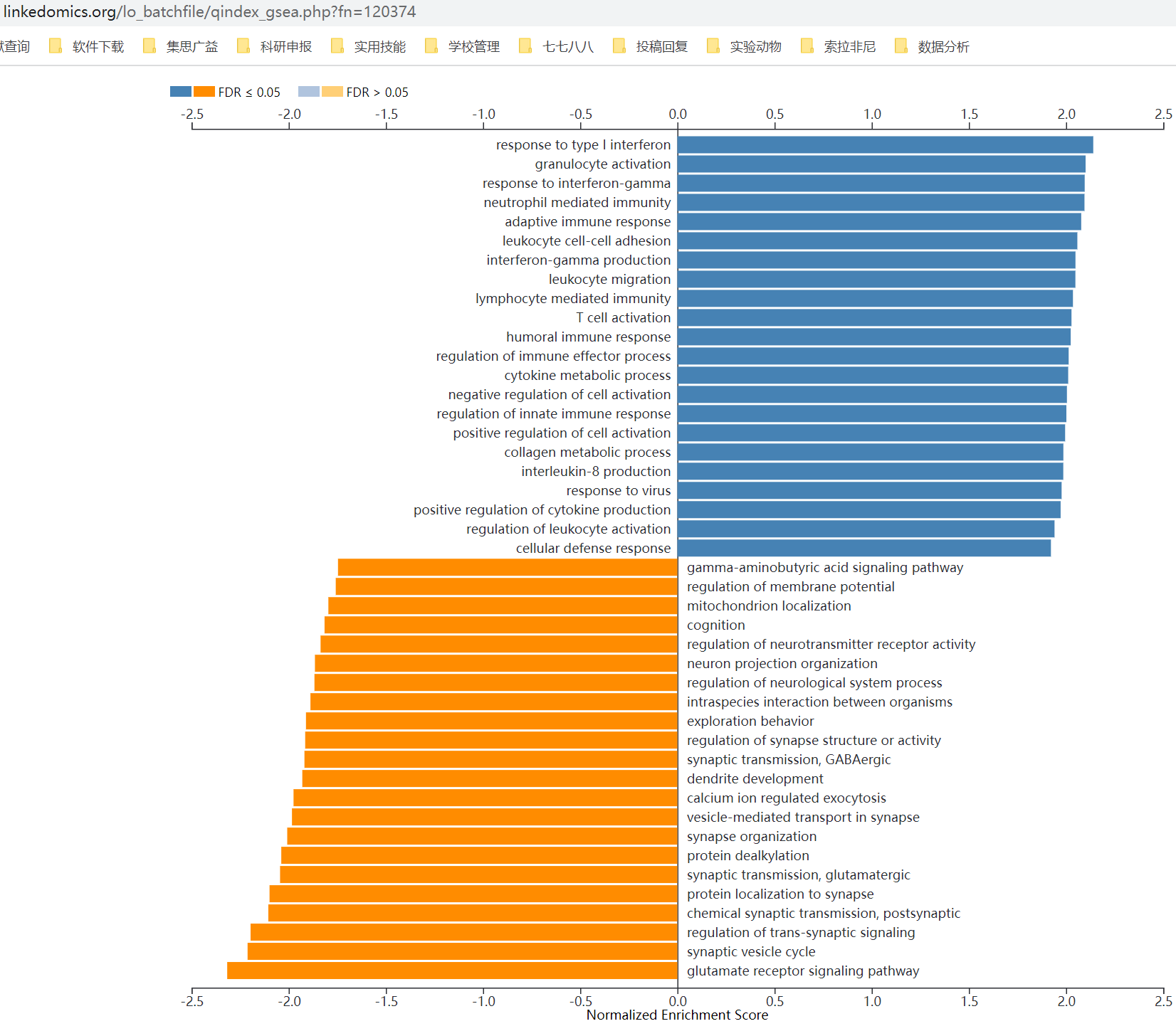


Figure 6E:


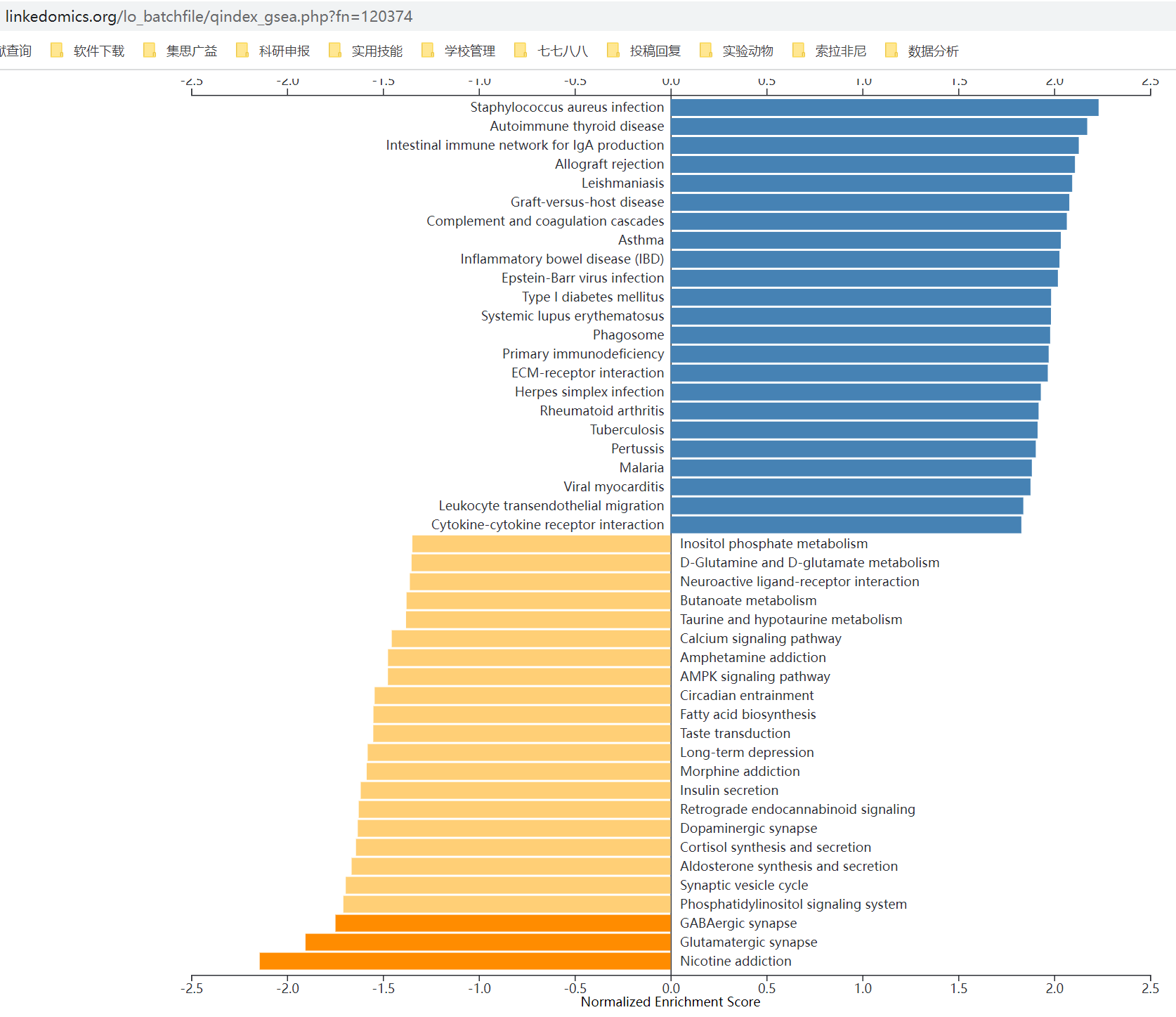


Figure 6F:


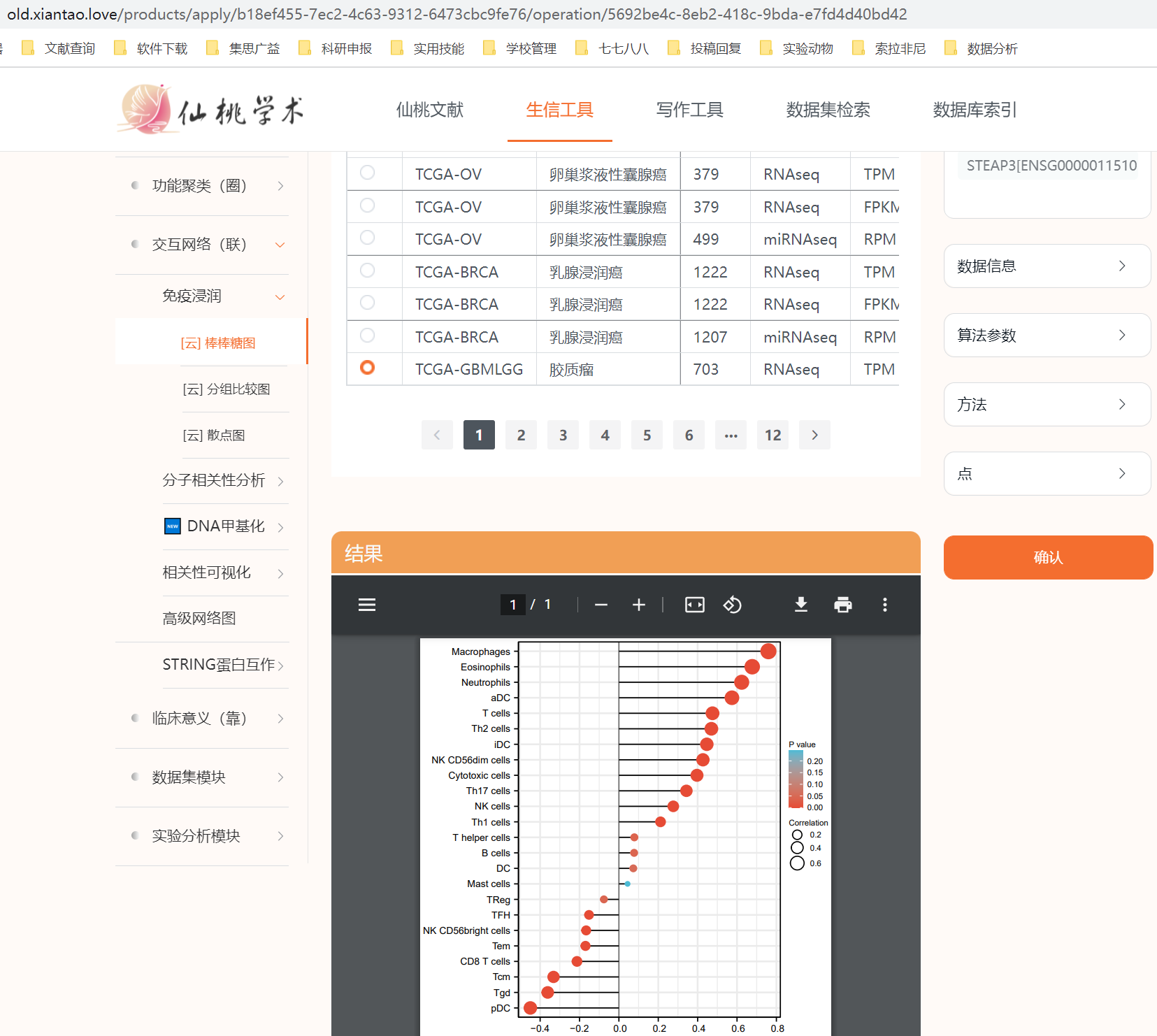


Figure 6G:


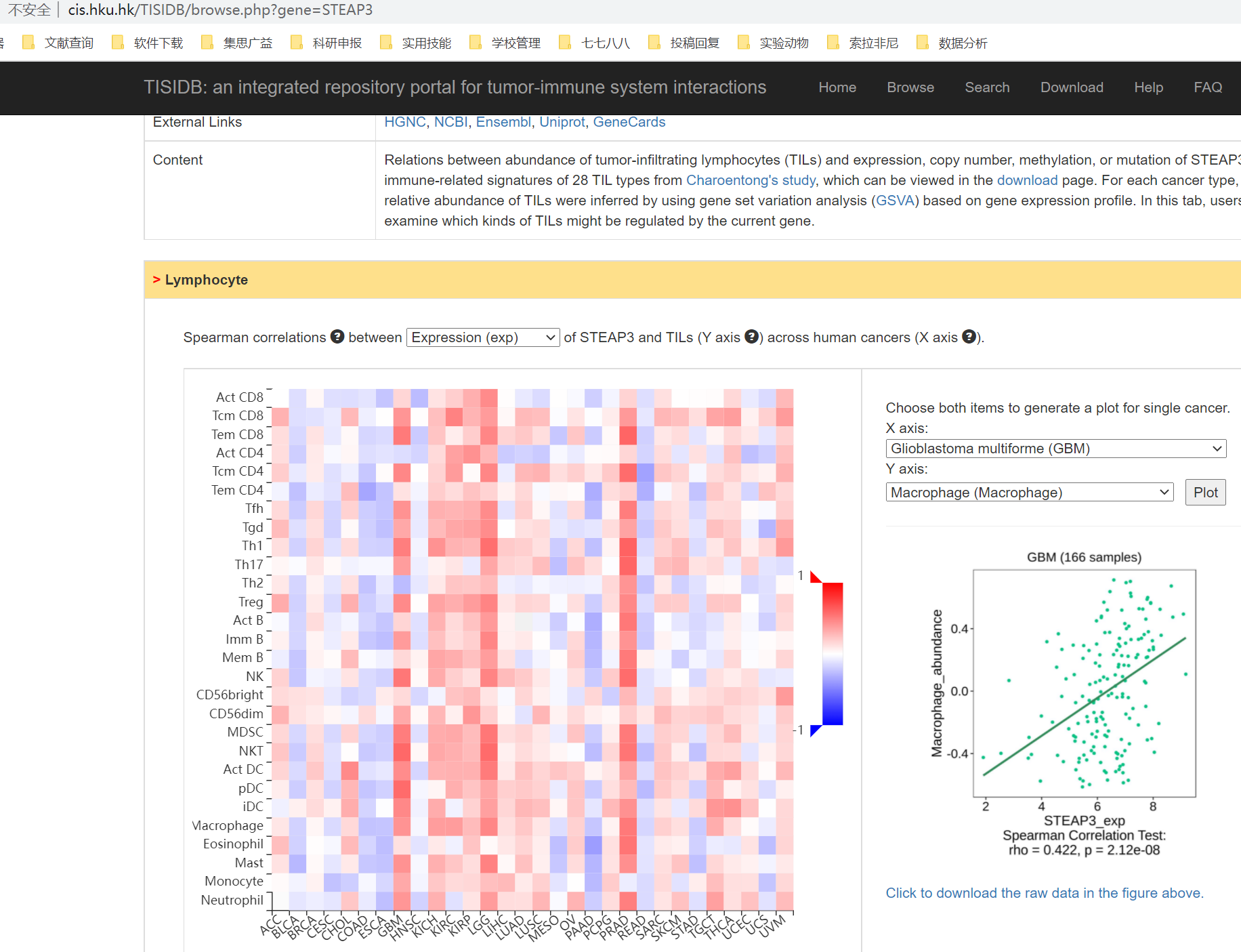


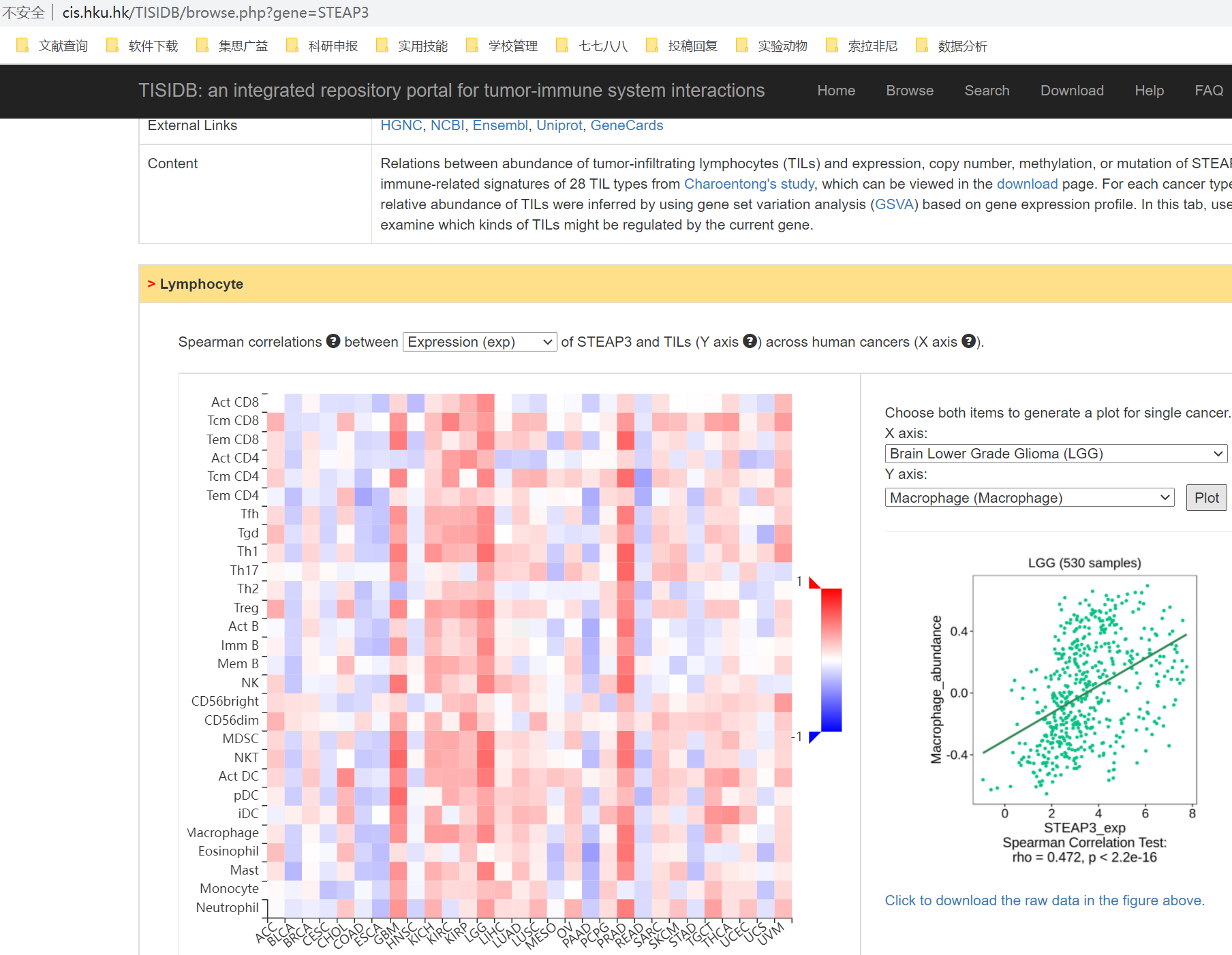


Figure 6H:


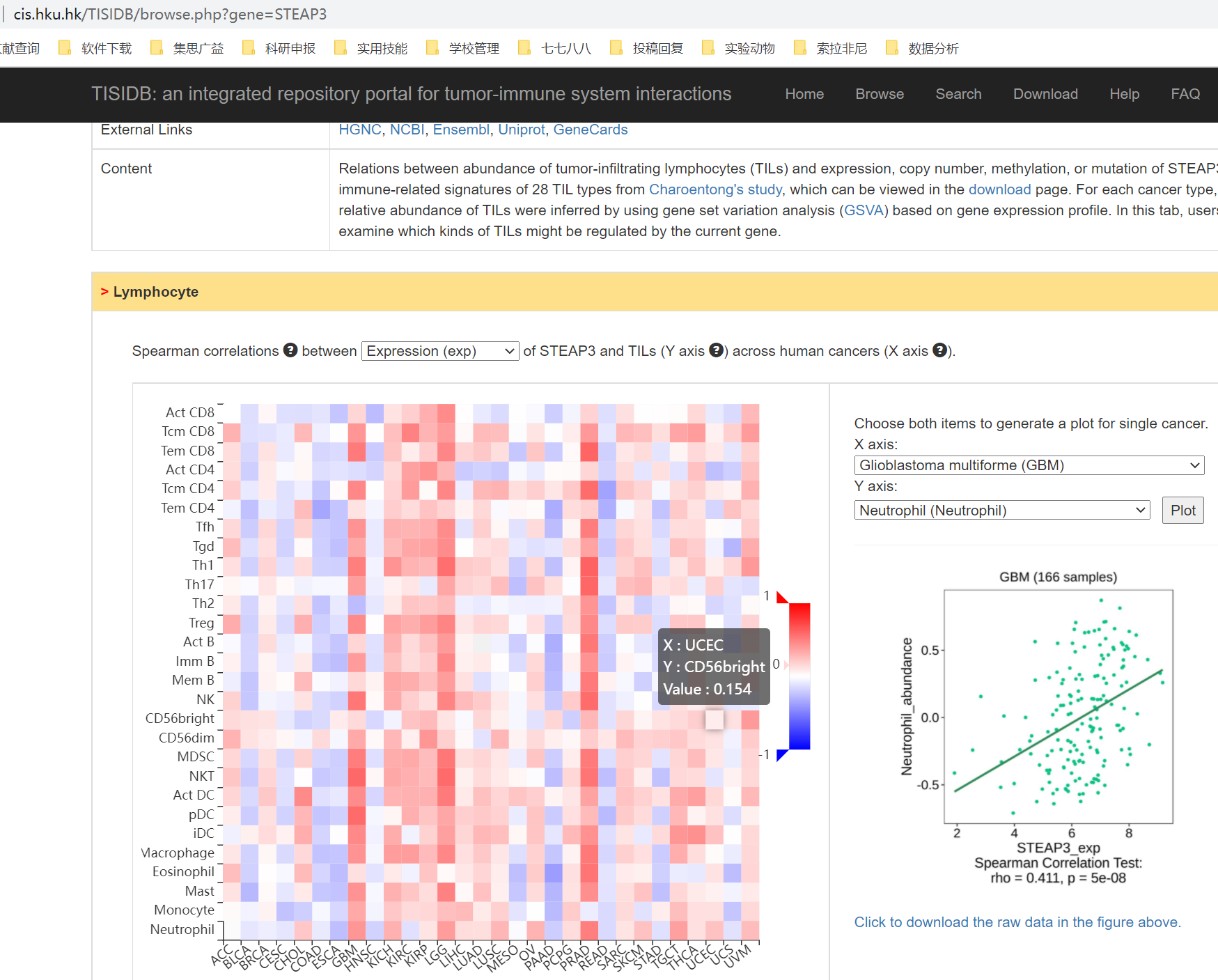


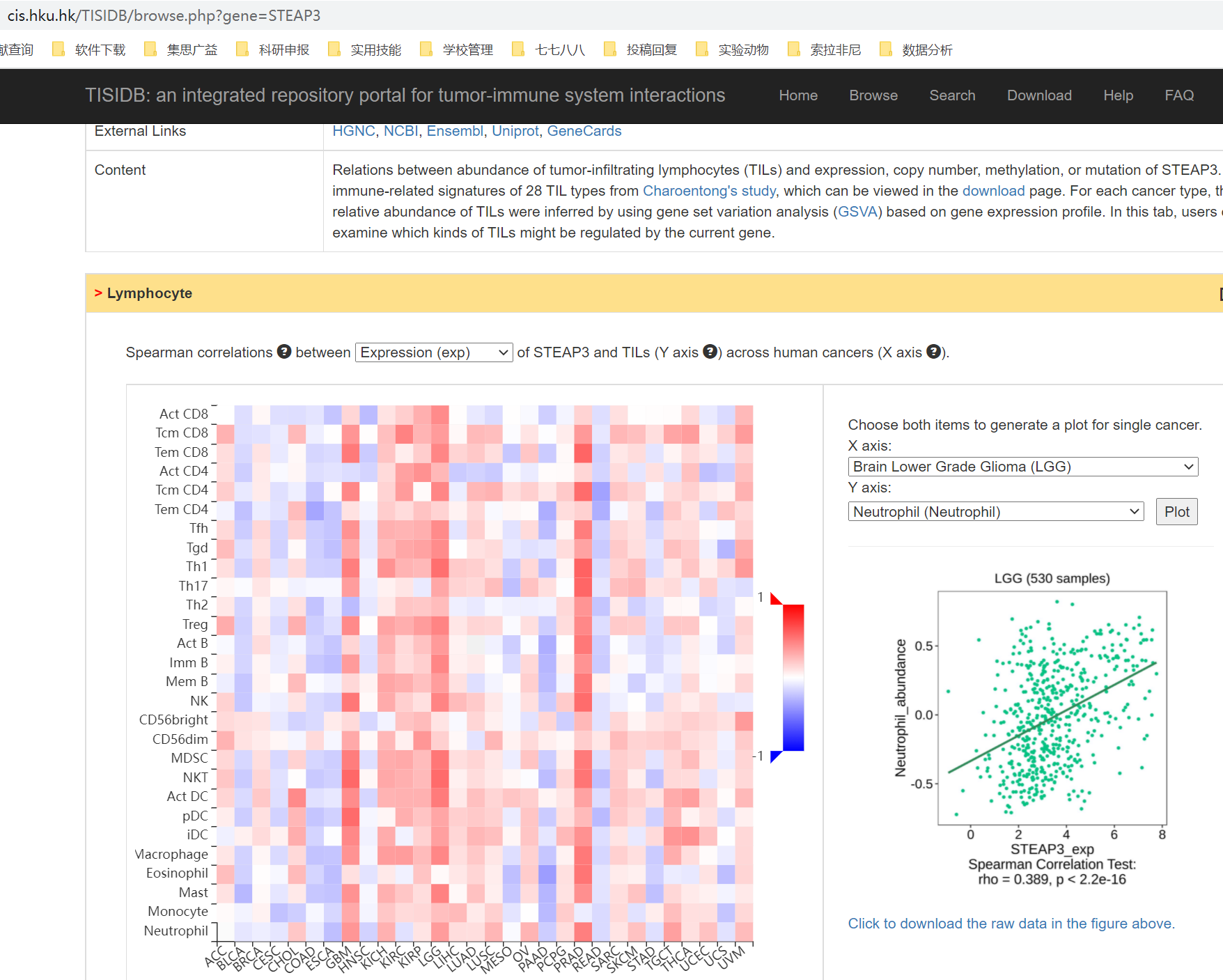

Supplement: Supplemental Information 11 — STEAP3 co-expression network and GSEA enrichment analysis. [file peerj-11-15136-s011.zip › raw data for Figure 6/Raw data for Figure 6A-H.docx]
