# Supplementary material for: High expression of six-transmembrane epithelial antigen of prostate 3 promotes the migration and invasion and predicts unfavorable prognosis in glioma
Source: PeerJ. 2023 Mar 28;11:e15136. doi: 10.7717/peerj.15136 (PMC10065001; doi:10.7717/peerj.15136)
Supplement: Supplemental Information 13 — The immunohistochemical analysis and prognostic value of STEAP3 in tumor. [file peerj-11-15136-s013.zip › raw data for Supplementary Figure S2-3/raw data for Supplementary Figure S2.docx]

Supplementary Figure S2

Tonsil normal

<https://www.proteinatlas.org/ENSG00000115107-STEAP3/tissue/tonsil#img>

HNSC

<https://www.proteinatlas.org/ENSG00000115107-STEAP3/pathology/head+and+neck+cancer#img>
