# Supplementary material for: High expression of six-transmembrane epithelial antigen of prostate 3 promotes the migration and invasion and predicts unfavorable prognosis in glioma
Source: PeerJ. 2023 Mar 28;11:e15136. doi: 10.7717/peerj.15136 (PMC10065001; doi:10.7717/peerj.15136)

Supplementary Figure S3A:

Overall survival


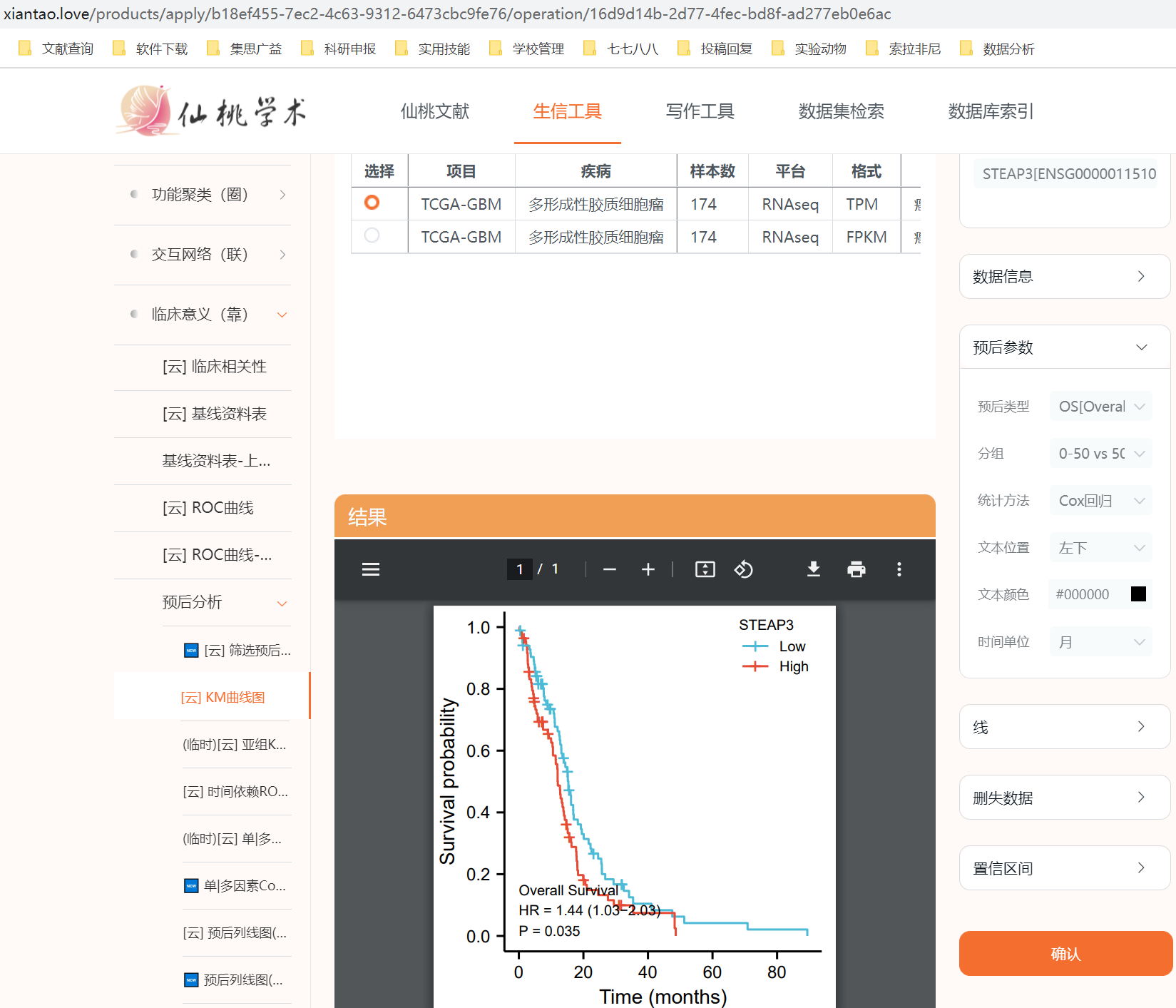


Disease specific survival


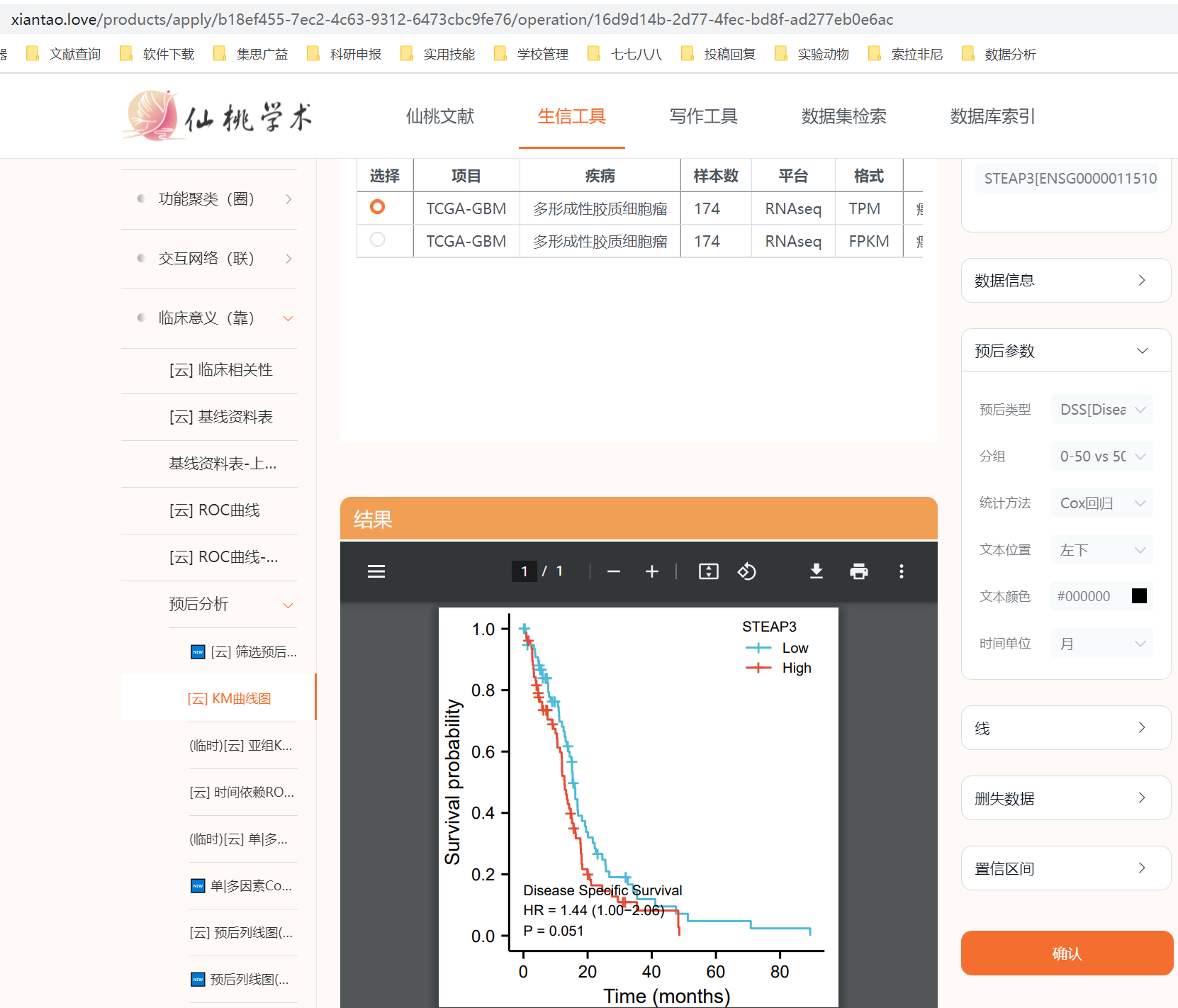


Progression free survival


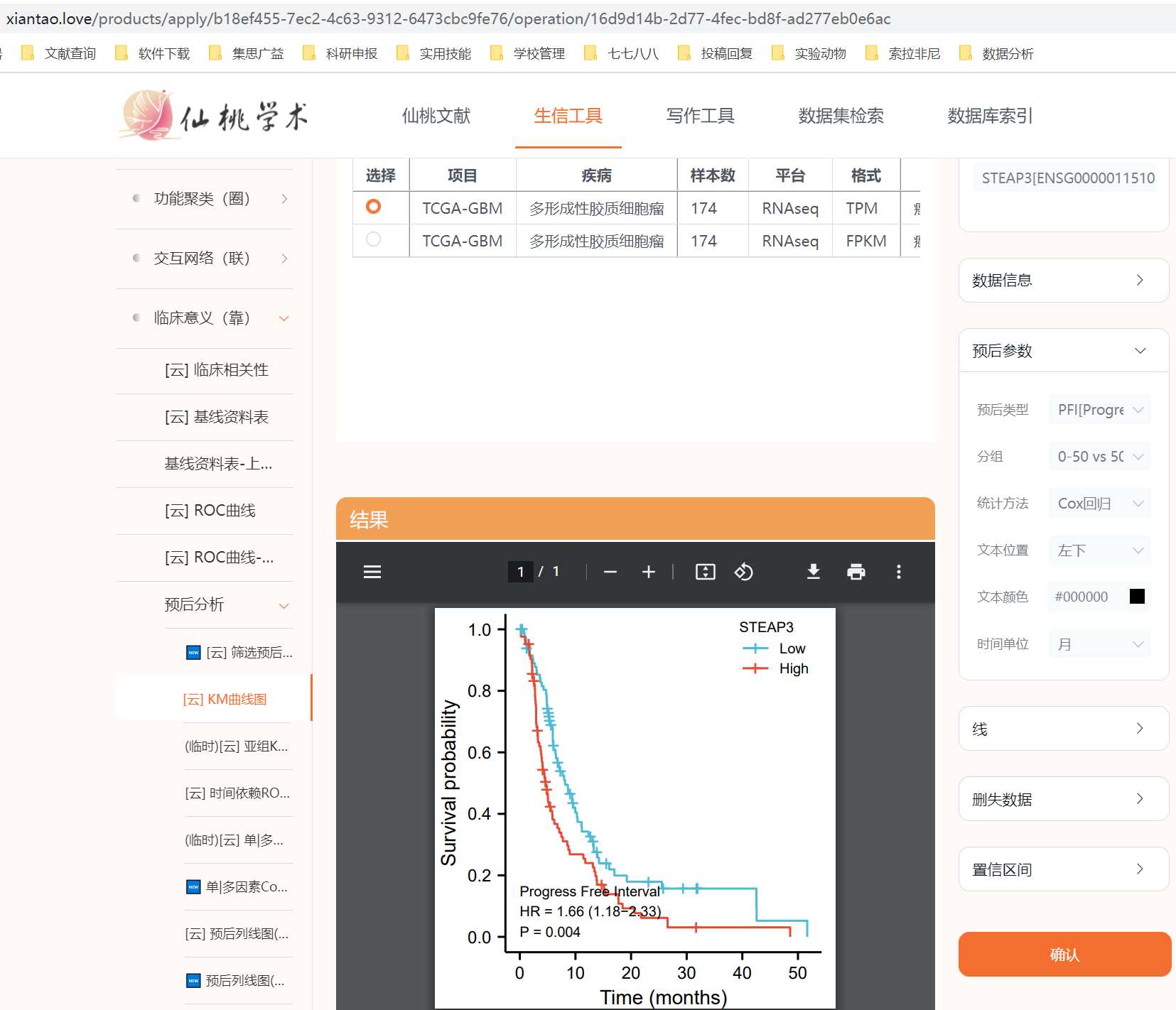


Supplementary Figure S3B:

Overall survival


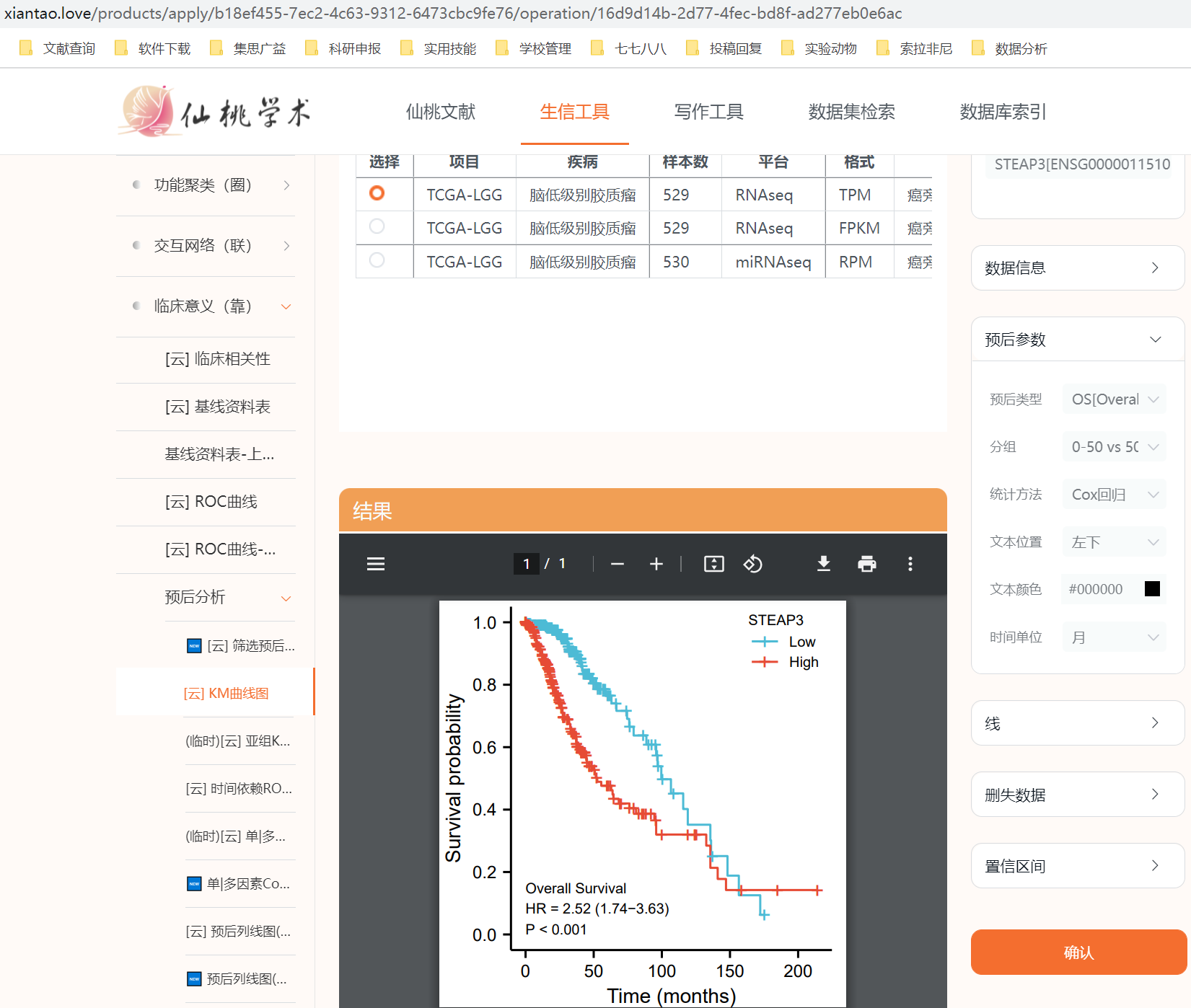


Disease specific survival


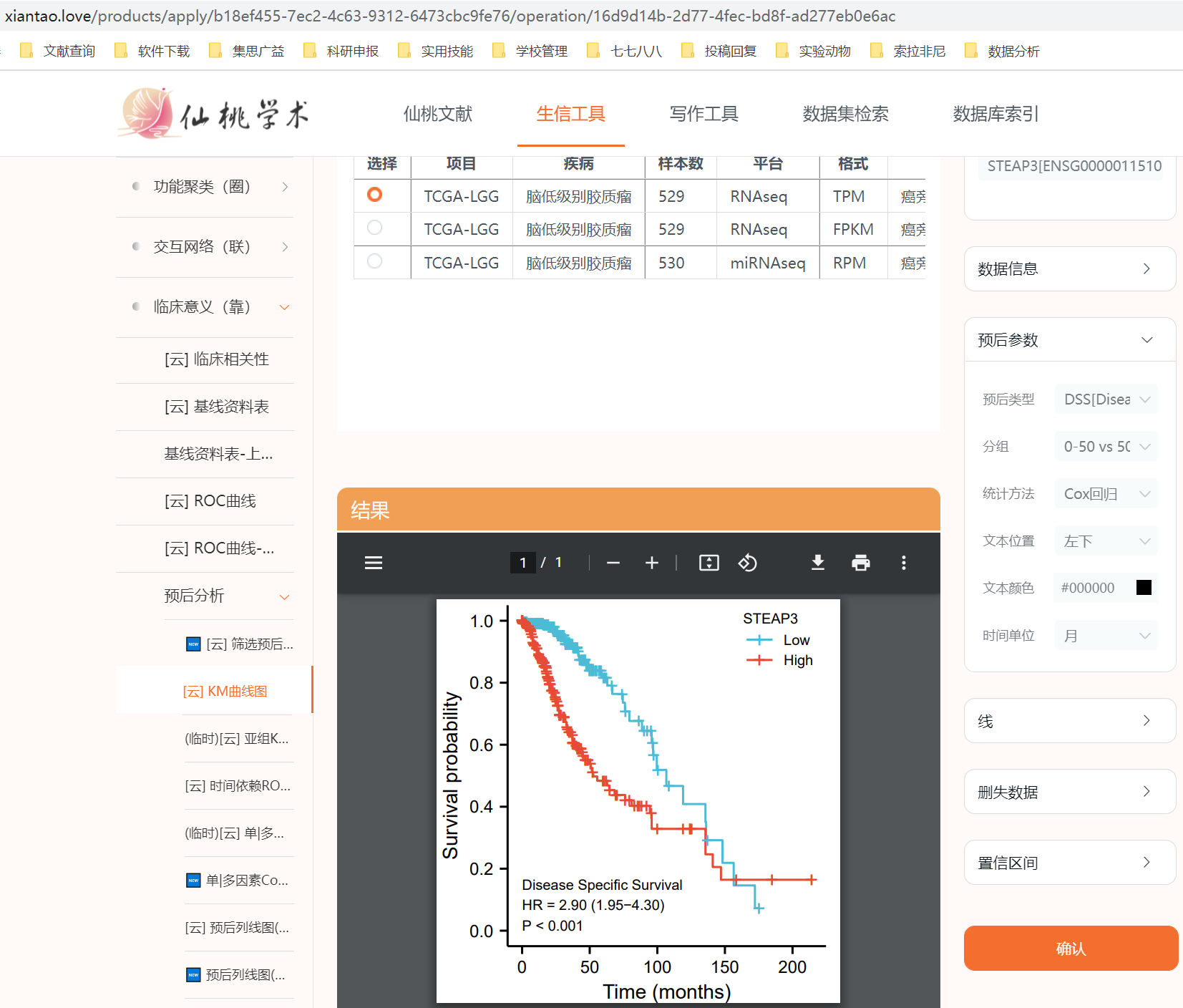


Progression free survival


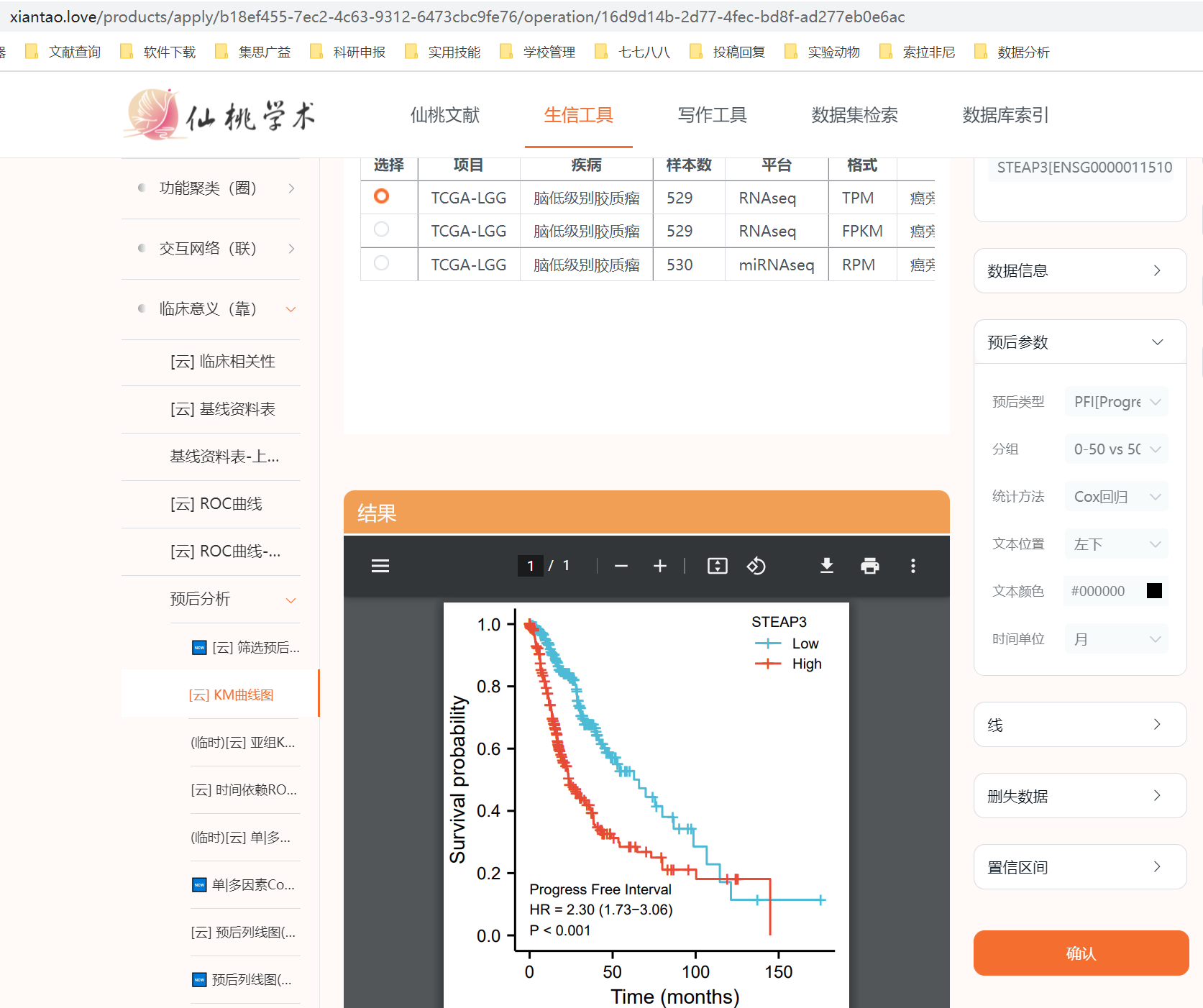

Supplement: Supplemental Information 13 — The immunohistochemical analysis and prognostic value of STEAP3 in tumor. [file peerj-11-15136-s013.zip › raw data for Supplementary Figure S2-3/raw data for Supplementary Figure S3.docx]
